# Special Issue: Transforming Nursing in South Africa

**DOI:** 10.3402/gha.v8.28205

**Published:** 2015-05-11

**Authors:** Laetitia C. Rispel

**Affiliations:** University of the Witwatersrand, Johannesburg, South Africa

Human Resources for Health (HRH) are critical to health systems development and functioning, and to patient and population health outcomes (1). Nurses in South Africa, as elsewhere, make up the largest single group of health service providers and their role in promoting health and providing essential health services is undisputed (2). South Africa has three categories of nurses: professional (registered) nurses with 4 years of training; enrolled nurses with 2 years of training; and nursing assistants or auxiliaries with 1 year of training. The majority of professional (registered) nurses are also midwives, and the terms ‘nurse’ and ‘midwife’ are used interchangeably in the Nursing Act (3).

However, the country faces a ‘nursing crisis’, characterised by shortages, declining interest in the profession, lack of a caring ethos, and an apparent disjuncture between the needs of nurses on the one hand and those of communities served on the other hand (4, 5). The context of this nursing crisis is South Africa's quadruple disease burden (6), the multiplicity of health sector reforms (7), gender stratification, and the existence of strong professional silos and hierarchies (8). Progress towards universal health coverage (UHC) in South Africa, which aims to ensure that everyone is able to access the health care services they need irrespective of their ability to pay (9), is dependent on addressing these nursing challenges.

In July 2008, the Centre for Health Policy (CHP) in the School of Public Health, University of the Witwatersrand, Johannesburg, obtained funding from The Atlantic Philanthropies (AP) for a four-year research programme to develop and strengthen the research evidence for improved nursing policy development and practice in South Africa. Known popularly as Research on the State of Nursing (RESON), the overall research programme consisted of three focus areas: nursing management and quality of care; nursing policy; and the process of casualisation in nursing, specifically nursing agencies and moonlighting, and how these have an impact on nurses and on the health system. Capacity building and policy influence and engagement were important cross-cutting focus areas.

In this Special Issue of *Global Health Action*, we present a series of papers that describe selected research findings of the various components of the RESON project. The Special Issue brings together 11 scholarly articles that explore themes that are relevant to a global audience of nurses and health policy-makers: nursing education reforms; enhancing the participation of nurses in policy-making; moonlighting among nurses; utilisation of agency nurses in hospitals and its costs to the health sector (both direct and indirect); ethics; quality of care; and the work experiences of nursing managers at primary health care clinics.

## Themes and focus of the Special Issue

### Nursing education, reforms, and health policy participation

Three papers under this theme focus on nursing education, reforms in nursing education, and nurses’ participation in policy-making.

In the first paper, Armstrong and Rispel use a social accountability framework, specifically the World Health Organization's six building blocks for transformative education (10), to explore key informants’ perspectives on nursing education in South Africa (11). Study participants acknowledged that South Africa has strategic plans on human resources for health and nursing education, training, and practice (11). There is also a well-established system of regulation and accreditation of nursing education through the South African Nursing Council (SANC) (11). However, key informants criticised the lack of national staffing norms, sub-optimal governance by both the SANC and the Department of Health, outdated curricula that are unresponsive to population and health system needs, lack of preparedness of nurse educators, and the perceived unsuitability of the majority of nursing students (11). These issues would need to be addressed in order to enhance social accountability, which is an essential component of transformative education (11).

Using a policy analysis framework, Blaauw et al. analyse nursing education reforms that culminated in a new Framework for Nursing Qualifications in 2013 (12). The revision of nursing qualifications was part of the post-apartheid transformation of nursing but was also influenced by changes in South Africa's higher education sector (12). The two most important changes are the requirement for a baccalaureate degree to qualify as a professional nurse and the abolition of the enrolled nurse with 2 years of training in favour of a staff nurse with a 3-year college diploma (12). The policy process took more than 10 years to complete and the final Regulations were promulgated in 2013. Respondents criticised slow progress, weak governance by the SANC and the Department of Health, limited planning for implementation, and the inappropriateness of the proposals for South Africa (12).

In the third paper within this theme, Ditlopo et al. (13) analyse the dynamics, strengths, and weaknesses of nurses’ participation in four national health workforce policies: the 2008 Nursing Strategy, revision of the Scope of Practice for nurses, the new Framework for Nursing Qualifications, and the Occupation-Specific Dispensation (OSD) remuneration policy. The study found that nurses’ participation in policy-making is both contested and complex (13). There was a disjuncture between nursing leadership and front-line nurses in their levels of awareness of the four policies (13). There was also limited consensus on which nursing group legitimately represented nursing issues in the policy arena (13).

### Understanding casualisation in nursing

The second theme focuses on the process of casualisation in nursing, specifically the relationship between moonlighting and intention to leave, the potential health system consequences of agency nursing and moonlighting, the direct and indirect costs of nursing agencies, and the characteristics of nursing agencies in South Africa.

In the first paper, Rispel et al., examine whether moonlighting is a determinant of South African nurses’ intention to leave their primary jobs (14). The study found that almost one third of 3,784 survey participants (30.9%) indicated that they planned to leave their jobs within the 12 months following the survey. Intention to leave was higher among the moonlighters (39.5%) compared to non-moonlighters (27.9%) (14). Moonlighting was found to be a predictor of intention to leave (14) and would need to be addressed as part of nurse retention strategies.

In the second paper, Rispel and Blaauw examine the potential health system consequences of agency nursing and moonlighting among South African nurses (15). In the cross-sectional survey of 3,784 participants, 40.7% of nurses reported moonlighting or working for an agency in the year preceding the survey, 51.5% of all participants reported feeling too tired to work, 11.5% paid less attention to nursing work on duty, while 10.9% took sick leave when not actually sick in the preceding year (15). In a multiple logistic regression analysis, the differences between moonlighters and non-moonlighters were not statistically significant after adjusting for other socio-demographic variables. The authors conclude that although moonlighting did not emerge as a statistically significant predictor, the reported health system consequences are serious and need to be addressed by health managers and policy-makers (15).

Rispel and Angelides conducted a provincial survey on nursing agency utilisation and analysed provincial health expenditure on nursing agencies from 2005 until 2010 (16) in the third paper on casualisation in nursing. The study found that 1.49 billion South African Rands (R) (US $ 212.64 million) was spent on nursing agencies in the public health sector in the 2009/10 financial year (16). In the same period, agency expenditure ranged from a low of R36.45 million ($5.20 million) in Mpumalanga Province (mixed urban–rural) to a high of R356.43 million ($50.92 million) in the Eastern Cape Province (mixed urban–rural). In that financial year, a total of 5,369 registered nurses could have been employed in lieu of nursing agency expenditure (16).

However, there are also indirect costs associated with agency nursing. Complementing the direct costing study, Rispel and Moorman (17) examine the direct and indirect costs of agency nurses, as well as the advantages and the problems associated with agency nurse utilisation in two public sector hospitals in South Africa. The study found that the indirect cost activities at both hospitals in 1 week exceeded the weekly direct costs of nursing agencies. Agency nurses assisted the selected hospitals in dealing with problems of nurse recruitment, absenteeism, shortages, and skills gaps in specialised clinical areas. The problems experienced with agency nurses included their perceived lack of commitment, unreliability, and providing sub-optimal quality of patient care (17).

Olojede and Rispel, in the final paper within the casualisation theme, explore the characteristics of nursing agencies in South Africa, and their relationship with clients in the health sector (18). Although a small sample of nursing agencies was selected, the study found that 27% of these nursing agencies provided services to homes for elderly people (18). Nursing agencies were more likely to have contracts with private sector clients (84%), than with public sector clients (16%) (18). In terms of quality checks and monitoring, 81% of agencies agreed with a statement that they checked the Nursing Council registration of nurses, 82% agreed with a statement that they requested certified copies of a nurse's qualifications, but only 21% indicated that they conducted reference checks of nurses with their past employers (18).

### Ethics, quality of care, and work experiences of nursing managers

Three diverse papers conclude the special journal issue, but all three highlight the importance of an enabling practice environment for nurses.

White et al. explore hospital nurses’ perceptions of the international Code of Ethics for nurses, their perceptions of the South African Nurses’ Pledge of Service, and their views on ethical practice (19). The majority of study participants agreed with a statement that they will promote the human rights of individuals (98%), and that they have a duty to meet the health and social needs of the public (96%) (19). More nuanced responses were obtained for some questions, with 60% agreeing with a statement that too much emphasis is placed on patients’ rights as opposed to nurses’ rights and 32% agreeing with a statement that they would take part in strike action to improve nurses’ salaries and working conditions. The dilemmas faced by nurses in upholding the Code of Ethics and the Pledge in the face of workplace constraints or poor working conditions were revealed in nurses’ responses to open-ended questions (19).

Munyewende and Rispel explore the work experiences of PHC clinic nursing managers through the use of reflective diaries (20). Although inter-related and not mutually exclusive, the main themes that emerged from the diary analysis were: health system deficiencies; human resource challenges; unsupportive management environment; leadership and governance and the emotional impact on the manager (20).

In the final paper in the Special Issue, Armstrong et al. examine whether the activities of nursing unit managers facilitate the provision of quality patient care in South African hospitals (21). The study found that nursing unit managers spent 25.8% of their time on direct patient care, 16% on hospital administration, 14% on patient administration, 3.6% on education, 13.4% on support and communication, 3.9% on managing stock and equipment, 11.5% on staff management, and 11.8% on miscellaneous activities (21). These managers also experienced numerous interruptions and distractions (21).

## Conclusion

In this Special Issue we have reported on the findings of selected research studies done as part of RESON. Using different methodologies that range from the use of reflective diaries to large cross-sectional surveys, the papers in this volume have enhanced our knowledge and understanding of nursing in South Africa in three research areas: policy analysis; casualisation, agency nursing, and moonlighting; and nursing management and practice environments.

A common strand that runs through all the papers is the importance of leadership, governance, and management from three important policy actors: national government; the Nursing Council; and the national nursing association (11–21). The appointment of the Chief Nursing Officer in 2014 is encouraging. A revised Strategic Plan on Nursing Education, Training, and Practice is in place (2). The existence of SANC and a sound regulatory framework are positive aspects, as is the presence of a strong national nursing association. Hence, there is a good foundation to provide stewardship for the implementation of the recommendations contained in this volume, which are based on empirical findings and complement those contained in the Strategic Plan (2). At the same time, the weaknesses in the policy capacity of the main institutions responsible for the leadership and governance of nursing in South Africa will need to be addressed if health sector reforms are to be realised (12).

The Special Issue underscores the importance of conducting health workforce research that contributes to health system transformation, and highlights neglected policy issues, such as the process of casualisation in nursing, and the implications for the health system. As is the case with many health workforce studies, the papers in this volume are based on cross-sectional rather than longitudinal data. There is need for investment in long-term, longitudinal studies of the health workforce to enable measurement of progress over time and enhance research contribution to evidence-informed health policy-making.

The papers also highlight the importance of involving nurses at the coal-face of service delivery and continuing professional development to enable nursing managers to lead the provision of consistent and high-quality patient care (20, 21).

Many of the issues foregrounded in this Special Issue have relevance for nurses and health policy-makers in other low- and middle-income countries. Collectively, the study findings suggest that South Africa's quest for UHC to improve population health and achieve equity and social justice cannot be achieved unless the issues facing nurses and nursing in South Africa are confronted.

*Laetitia C. Rispel* Centre for Health Policy & Medical Research Council Health Policy Research Group School of Public Health Faculty of Health Sciences University of the Witwatersrand 27 St. Andrew's Road, Parktown, 2193 Johannesburg, South Africa
