# Supplementary material for: Special Issue: Transforming Nursing in South Africa
Source: Glob Health Action. 2015 May 11;8:10.3402/gha.v8.28205. doi: 10.3402/gha.v8.28205 (PMC4430691; doi:10.3402/gha.v8.28205)
Supplement: Related texts

# Sub article: Social accountability and nursing education in South Africa

**DOI:** 10.3402/gha.v8.27879

**Published:** 2015-05-11

**Authors:** Susan J. Armstrong, Laetitia C. Rispel

**Affiliations:** 1 Centre for Health Policy & Medical Research Council Health Policy Research Group, School of Public Health, Faculty of Health Sciences, University of the Witwatersrand, Johannesburg, South Africa; 2 Department of Nursing Education, School of Therapeutic Sciences, Faculty of Health Sciences, University of the Witwatersrand, South Africa

**Keywords:** nursing education, social accountability, nurses, transformative education, South Africa

## Abstract

**Background:**

There is global emphasis on transforming health workforce education in support of universal health coverage.

**Objective:**

This paper uses a social accountability framework, specifically the World Health Organization's six building blocks for transformative education, to explore key informants’ perspectives on nursing education in South Africa.

**Methods:**

Using a snowballing sampling technique, 44 key informants were selected purposively on the basis of their expertise or knowledge of the research area. Semi-structured interviews were conducted with the key informants after informed consent had been obtained. The interviews were analysed using template analysis.

**Results:**

South Africa has strategic plans on human resources for health and nursing education, training, and practice and has a well-established system of regulation and accreditation of nursing education through the South African Nursing Council (SANC). Key informants criticised the following: the lack of national staffing norms; sub-optimal governance by both the SANC and the Department of Health; outdated curricula that are unresponsive to population and health system needs; lack of preparedness of nurse educators; and the unsuitability of the majority of nursing students. These problems are exacerbated by a perceived lack of prioritisation of nursing, resource constraints in both the nursing education institutions and the health training facilities, and general implementation inertia.

**Conclusion:**

Social accountability, which is an essential component of transformative education, necessitates that attention be paid to the issues of governance, responsive curricula, educator preparedness, and appropriate student recruitment and selection.

As the largest single group of health-care providers in any country, nurses have the potential to bridge the gap between communities and the health-care system, coordinate care for patients with increasingly complex disease profiles, and accelerate the achievement of universal health coverage (1–4). However, the Institute of Medicine in the United States has pointed out that such major health system changes will require equally profound changes in the education of nurses to prepare them for new and transformed roles and responsibilities (4).

Around the world, the discourse on the education of health professionals has intensified, focusing on the need to increase numbers and to improve the quality, relevance, and retention of graduates (3, 5–8). However, the call for the scale-up of education programmes has been accompanied by a clear enunciation of systemic challenges in health professional education (5, 8). These challenges include the following: the mismatch between professional competencies and patient and population health priorities; poor teamwork; insufficient emphasis on primary health care (PHC); maldistribution of health-care professionals; and weak stewardship and leadership to improve health system performance (5, 8).

Nonetheless, there is a convergence of ideas that these challenges could be overcome through the transformative scale-up of medical, nursing, and midwifery education (3–8). Critical aspects of such transformative scale-up include: relevance and responsiveness to community health needs; increasing the number of professional health workers and training institutions; and reforms in recruitment, teaching methods, and curricula in order to improve the quality and social accountability of graduates (8). Social accountability in this context is defined as ‘the obligation of medical schools to direct their education, research and service activities towards addressing the priority health concerns of the community, region, and /or nation they have the mandate to serve. The priority health concerns are to be identified jointly by governments, healthcare organisations, health professionals, and the public’ (9, p. 3). Social accountability is a key component of the World Health Organization's Global Toolkit for Evaluating Health Workforce Education (10). The toolkit proffers the six building blocks for transformative education as being: health workforce planning; governance, policy, and funding; national standards on accreditation, regulation, and vocational qualifications; curricula, faculty, and education; career and retention; and student selection (10).

There is a significant and diverse body of literature on nursing education, focusing *inter alia* on the following: professionalisation through the shift from a hospital-based apprenticeship model to baccalaureate nursing degrees in higher education institutions (11–22); nursing education theories (23); debates on competencies in pre-service training (17, 24–29); curriculum development for nurse educators (30–32); and student selection, experiences, and perceptions of the learning environment (33–38). In contrast to medical education where the notion of social accountability has put medical schools under the spotlight (39–43), there has been relatively little emphasis on the social accountability of nursing education institutions or their graduates. For example, there is no mention of social accountability in the 2009 global standards for the initial education of professional nurses and midwives (44). Although South Africa's 2012/13 Strategic Plan on Nursing Education, Training and Practice proposed that nursing education should be regarded as a national competence in order to enhance social accountability, this plan has not been implemented (45).

In South Africa, there are three categories of nurses: professional (registered) nurses with 4 years of training; enrolled nurses with 2 years of training, and nursing assistants or auxiliaries with 1 year of training. The majority of professional (registered) nurses are also midwives, and the terms ‘nurse’ and ‘midwife’ are used interchangeably in the Nursing Act (46). Nursing education is undergoing major changes, notably the requirement that a baccalaureate degree is a precondition for registration as a professional nurse, and the abolition of the enrolled nurse with 2 years of training in favour of a staff nurse with a 3-year college diploma (47). Nursing education takes place in a complex environment, which includes 20 out of 23 public universities, 12 public-sector nursing colleges (with numerous satellite training campuses) that are the responsibility of the nine provincial health departments, a nursing college run by the defence force, private nursing colleges run by the three major private hospital groups in South Africa, and private nursing schools that are run for profit (45, 48). This environment creates considerable fragmentation, and various layers of complexity. The public universities and public-sector nursing colleges are the only institutions that are allowed by law to offer the integrated course leading to registration as a professional nurse, which includes general, community health nursing, psychiatric nursing, and midwifery, through a 4-year degree or diploma, respectively (46). The South African Nursing Council (SANC) is the regulatory authority responsible for setting standards and accrediting nursing education institutions against those standards (46). The SANC does not require newly qualified professional (registered) nurses with 4 years of training to write a national licencing examination, but relies on the nursing education institution's own quality assurance systems to ensure an acceptable standard of education and competencies (46). However, the reality is that the quality of nursing education for registered nurses differs across the different educational institutions, and even from one nursing college to another managed by the same provincial health department (48). There are even more variations in the training of enrolled nurses (with 2 years of training) and auxiliary nurses (with 1 year of training), although these two categories of nurses are required to write a national licencing examination.

Given the global discourse on transforming health workforce education in support of universal health coverage (10), this paper uses a social accountability framework, specifically the six building blocks for transformative education, to explore key informants’ perspectives on nursing education in South Africa, in order to enhance the discourse on appropriate nursing education reforms and to make recommendations for policy implementation. The findings are part of a larger research project that examined casualisation of the nursing workforce (49, 50).

## Methods

The study was approved by the University of the Witwatersrand Human Research Ethics Committee (Medical). All participants received a study information sheet and gave informed written consent. Using a snowballing sampling technique, 44 key informants were selected purposively on the basis of their expertise or knowledge of the research area.

Semi-structured interviews were conducted with the key informants after informed consent had been obtained, using an interview guide developed specifically for the study by the research team and an advisory committee. In addition to questions on casualisation, the interview covered the following: achievements or progress by nurses or in nursing; challenges faced by nurses, both individually and by the nursing profession as a group; perceptions of changes in nursing in the 5 to 10 years preceding the interview; and recommendations for change. A member of the research team contacted each person identified as a potential key informant and requested an interview. If the person agreed to be interviewed, an appointment was set up for an interview at a place and time convenient for the informant. All interviews were conducted in English. Interviewers used probes to clarify responses and to obtain more detailed information.

Two researchers conducted the interviews between June and October 2009. Each key informant interview lasted about one hour, although the duration varied in some cases depending on the responses by informants. Interviews were recorded digitally, but interviewers also took detailed notes during the interview and wrote a synopsis of the interview. In some instances, respondents provided additional, relevant documentation to the interviewers.

The recorded interviews were transcribed verbatim. Data cleaning took 1 month and consisted of an iterative process of checking the transcribed interviews against the original recordings, correcting the text, checking the recordings again, and making final corrections. Prior to analysis, an audit of key informants was done, and each key informant was allocated a code number to ensure confidentiality of information. The coded interviews were consolidated into one file for ease of analysis.

The qualitative data were analysed using template analysis, which is a particular form of thematic content analysis (51). The WHO Global Toolkit for Evaluating Health Workforce Education was used as a template for the analysis of the key informant responses (10). The six building blocks – health workforce planning; governance policy and funding; national standards on accreditation, regulation, and vocational qualifications; curricula, faculty, and education; career and retention; and student selection – were used as *a priori* themes that were determined in advance of coding (51).

## Results

The 44 key informants were drawn from six categories: national and provincial government officials (*n=*16); executive nursing managers from the private sector or non-governmental organisations (*n=*10); nursing academics in universities or nursing colleges (*n=*8); representatives from the nursing council, nursing association, and/or health sector unions (*n=*5); and other health professionals, mainly doctors (*n=*5). Only two key informants from two health sector unions failed to respond to repeated requests for an interview.

The results on nursing education that emerged from the key informant interviews are presented according to the six building blocks for transformative health professional education (10).

### Health workforce planning

This building block measures the existence of information on the health status of the population or of individual subgroups as well as detailed information on health workforce demand and need, because these have an impact on health professional education (10). At the time of the study, there was a national Strategic Plan for Human Resources for Health (52), as well as a national Nursing Strategy (53). However, there were no national norms and standards for different categories of health workers, and several key informants commented on this gap, which has an impact on education and service delivery.

There are no staffing norms – it makes it difficult when opening new services – there needs to be broad consultation [on national norms]; it should be done in line with what is available [health workers] and link it up to the budget. (KI 14, provincial government manager, Gauteng)

I think that before we start to look at moonlighting the first thing we have to get [are] proper norms and standards … we are just working with what different people are saying and doing, but we haven't come up with proper staffing norms and standards for our own country and our own facilities … both in the private and public sectors. I am glad the Nursing Strategy is beginning to speak to that. (KI 16, private-sector nursing executive, Gauteng)

The staffing norms are not in place to say this is how we would like to staff either a primary health care centre or the medical ward or the general ward. The staffing norms are either outdated or unknown to the managers. (KI 35, human resource manager, international organisation)

### Governance, policy, and funding

This building block focuses on broad governance arrangements for health professional education (including collaboration across government ministries), policy development, and information on the funding of health workforce education (10).

A substantial number of key informants were of the opinion that the sub-optimal leadership provided by the Nursing Council as the regulator was the primary cause of many of the nursing problems experienced, as can be seen in the following excerpts:

Our Nursing Council is dysfunctional … we basically can't increase our numbers, open new centres, start new courses. You know we've tried to get permission to increase our numbers and to start satellite centres, while we wait for the new Regulations to be published, but we're just not getting there. (KI 1, private-sector nursing executive, Western Cape)

The last few [Nursing] Councils that we've had, we've really had problems with leadership. We were hoping that the new Council would be different, but when I listen to some of the things that people say, it's not different. It's about self-interest or self-serving interest rather than looking at the interest of the profession. So what is it that we doing wrong? I don't know. I mean how do we change that kind of culture? (KI 8, nurse educator)

The SANC was also criticised for the perceived delays in the implementation of the revised scopes of practice for the different nursing categories and the related training Regulations that guide nursing education institutions with regard to the preparation of these nurses. Key informants indicated that such delays stifle attempts to improve or expand nursing curricula and contribute to a state of inertia in the nursing education system, which had remained static instead of changing to meet the needs of the population or the health system. The Nursing Council was also criticised for its apparent reluctance to work with other government structures responsible for higher education.

The National Department of Health was also blamed by the key informants for much of the lack of planning and coordination as well as slow implementation, which hampers nursing education from moving forward.

I think there's a huge urgency to have leadership to take that on [an increase in nurse production] … both from the National [Department of Health] and also from the Nursing Council. And the fact that both institutions continue to be in disarray is worrying because we so need leadership and nobody else can really do that. (KI 7, human resource researcher, Western Cape)

The provincial health departments responsible for the implementation of nursing education in the public sector were also criticised for exacerbating fragmentation and contributing to a feeling of despondency among nurses:

The political people [in the provinces] don't talk to one another about health because they own the colleges. They [provinces] and higher education [Ministry] are not talking to one another. We don't get the feedback. So it's a major vacuum of uncertainty and everybody is now getting despondent. (KI 42, nursing academic, Free State)

Although policy development is important, the problems of health workforce production were exacerbated by insufficient resources in the public health sector and what some termed ‘transformation fatigue’ among nurses.

I think the biggest challenge that I have seen facing all nurses, regardless of whether you are in a rural area or the urban area – nurses in the public sector, I don't know about the private sector – is the frequency of the policy initiatives that contradict each other, and don't get seen through and don't have proper support and all of that; these lead to incredible transformation fatigue. And what I've seen happen is that nurses simply withdraw. (KI 7, human resource researcher, Western Cape)

Key informants were also of the opinion that there is lack of prioritisation of nursing, illustrated by the lack of funding for nursing strategic priorities. A senior provincial government manager commented as follows:

We keep on saying that nursing is the backbone of the health system, but when it comes to financing, it's difficult for them to finance nursing issues. For example, if we talk of training of nurse managers or the training of nurses, I have to go and knock on other people's doors and say, listen, I need to train nurses … but there is no specific budget. (KI 22, provincial government manager, Eastern Cape)

### National standards on accreditation, regulation, and vocational qualifications

This building block focuses *inter alia* on the need for accreditation, regulation, and quality control mechanisms for health workforce education in the country (10). In South Africa, the SANC is the regulatory authority for nursing education and practice and its mandate is prescribed by legislation (46). As indicated earlier, key informants commented on the sub-optimal functioning of the Nursing Council, which has an impact on the implementation of existing national standards, rather than the absence of accreditation.

The majority of key informants expressed concerns about the quality of teaching and learning, and there was some concern about the impending reforms in nursing education. In general, the medical doctors interviewed were of the opinion that the proposed reforms in nursing education have not been thought through, and one said the following:

I also do believe that training of [degree] nurses is not appropriate for what South Africa needs. I don't actually think we should be extending the university training. I think we should be extending the nursing colleges and training nurses for the primary function, that is, patient care and everything that goes around that. (KI 29, public-sector medical specialist, Gauteng)

The proposed Regulations also raised concerns among key informants about the lack of alignment between nursing training and the service or work environment. Some were of the opinion that nurses are inadequately prepared for the burden of disease that they face in hospitals or for working in a district health system.

### Curricula, faculty, and education

This building block focuses on appropriate curricula; community participation in health workforce education; the demographic profile of faculty and teaching staff; support for educators to engage in lifelong learning and continuing professional development; and community-based, interprofessional education based on principles of PHC and focusing on the social determinants of health (10).

Some of the key informants were of the opinion that nursing curricula are unresponsive to changes in disease burden and in the health system and that there is a disjuncture between government's emphasis on PHC and the training of nurses. These stated curriculum problems were exacerbated by the perception of the lack of skills among newly trained nurses, with many key informants pointing to skills gaps. These missing skills included inadequate social skills, lack of initiative, inability to apply theoretical knowledge to patient care, lack of basic nursing skills, and lack of understanding of professional practice.

Key informants also mentioned problems related to practical training, which included an insufficient number of good-quality practical training facilities, coordination of practical placements, and insufficient resources. A critical aspect mentioned was the lack of supervision and mentoring of students. One key informant said:

If I think back of our educational programmes, by the time you were a third-year nurse you would be in charge of the ward and run and manage the ward so well. We had mentors and we had people that we wanted to emulate … I don't think they [student nurses] have adequate mentors. They might be going out there to go and meet their clinical objectives but where are the mentors to [make sure] they achieve those clinical objectives? (KI 35, human resource manager, international organisation)

The key informants were critical of the quality of the nurse educators within many of the nursing education institutions, especially in the nursing colleges, who have not kept abreast of the practice environment and also lack modern teaching skills. There was a suggestion that because nurse educators have not kept abreast of these developments, they are not teaching up-to-date nursing procedures, which makes it difficult for the students to execute nursing tasks when they arrive in the hospitals. The shortages of nurse educators were seen as another significant challenge.

Really the training of nurse educators has been left behind. If we look at the standards, if we look at some of the resources in colleges, what the politicians expect … they [politicians] increase the [student] numbers by 100% but they don't provide the resources. (KI 40, nurse academic, North West)

The practical training is nearly non-existent and the supervision during training is scary and this is why they [nursing students] are not learning. They haven't got role models, they haven't got good teachers in the wards, and they are not learning how to do the job. The nurse educators are so busy trying to cope with these large groups now and they hardly ever get out into the hospitals, they themselves do not keep up to date, they are not even using up-to-date prescribed books or even South African-based prescribed books. (KI 20, provincial government manager, Gauteng)

### Career and retention

This building block focuses on the need to retain health workers particularly in underserved areas and to encourage their career development, as reflected in policies and plans and supportive measures in the workplace (10).

The majority of key informants pointed to resource constraints in nursing education institutions, particularly the lack of budgets, teaching infrastructure, student accommodation, and teaching equipment. Importantly, a similar lack of resources was experienced at the health facilities, which provide clinical training. Key informants pointed to the impact that resource constraints have on the quality of the nursing graduates. For example, in the public sector, they might not have exposure to new technology. In some instances, nursing education institutions based at universities have found creative alternatives, such as patient simulators. It was felt that the lack of resources has an impact on the retention of nurses, particularly in the public sector.

### Student selection

This building block focuses on student recruitment that is based on a combination of formal qualifications and transversal skills, reflective of underserved populations (10).

Key informants were very vocal about the problems of selection and suitability of applicants for entry into nursing. One said the following:

The other thing around [nursing] education is trying to get the right kind of nurse for this country. I think we put [too] much emphasis on the 4-year programme, whether a 4-year diploma or degree nurse – and they [nurses] get over-qualified and then they have aspirations to go all over. And what does the South African health-care system need in terms of the nurse? We really need to revisit the kind of nurse we need and what are the numbers we need to train. (KI 16, private-sector nursing executive, Gauteng)

There was a general perception that there is a problem in attracting nursing recruits who meet the selection criteria, because young people in South Africa have more career opportunities open to them. One commented as follows:

Nursing was a coveted job by young black women, so we used to get the best matriculants … so there was a time when we had quality people and they were in fact quite dedicated as well. Then I think over time there was an erosion of that, and it became a less attractive profession, poorly paid, long working hours, inflexible working hours, it lost its lustre and then young women looked for other career opportunities rather than doing nursing. (KI 29, medical specialist, Gauteng)

Key informants mentioned other factors that impact on student selection. These include the advanced age of students on commencement of training, which reduces the pool of nurses for further specialised training, and the relatively high attrition rate of students resulting in wastage of time and money in training them.

Almost one-third of the key informants were of the opinion that that the values and ethos of the current nursing students were different (and worse) compared with those held by nurses of the older generation and that the new generation was less caring towards patients and less committed to staying in nursing, as captured by the following quote:

I think the number one thing [problem] is the issue of the values in nursing, which perhaps may be different for different people but generally nursing was perceived to be a vocation for many of us – not the only choice of career. The values of caring for people and the importance of integrity and accountability were very important to us. (KI 19, retired nursing manager, Gauteng)

## Discussion

In South Africa, there is a well-established system of regulation and accreditation of nursing education through the SANC (46). This is commendable, as this is not the case in many low- and middle-income countries (3).

Using the six building blocks for transformative education and the voices of the 44 key informants, the study found sub-optimal governance on nursing education, thus confirming the findings of a policy analysis study that examined nursing education reforms in South Africa (47). The policy analysis study found slow progress, weak governance by the SANC and the Department of Health, and limited planning for implementation (47). A critical question that requires further research is why nurses do not demand greater accountability from the SANC and the Department of Health. The findings of the study underscore the importance of strategic leadership of key governance structures, such as the SANC. Such leadership will enable increased production of competent nurses, which will contribute to improved health system performance and ultimately to improved population health outcomes.

Key informants also pointed to problems with workforce planning, curricula, faculty and education, and student selection and training, which impact on nursing education. The issue of national staffing norms remains as important today as it was in 2009 when the interviews were conducted. Although South Africa has updated strategic plans on Human Resources for Health (54) as well as for Nursing Education, Training, and Practice (45), these do not contain detailed staffing norms. Staffing norms are not an end in themselves, but they provide guidance to managers and practitioners alike and assist with achieving equitable access to health-care providers, particularly in underserved urban and rural areas (55).

The comments from key informants suggest that curricula in nursing education institutions require revision so that they are more appropriate for the population and health system needs of South Africa. However, the lack of finalisation of the training Regulations and the scopes of practice was a major barrier to transformation of nursing education curricula. Although the training regulations were released at the end of 2012, the scopes of practice remain outstanding. The SANC continues to postpone the phasing-out of the legacy qualifications and, although the training of enrolled nursing auxiliaries and enrolled nurses will end in June 2015, a new date has been set for 2018 for the phasing-out of the programme leading to registration as a nurse (general, psychiatric, and community) and midwife. Hence, the implementation inertia described by some key informants continues. This inertia means that there has been little progress in implementing the 2009 global standards on nursing and midwifery education with regard to curriculum reform (44).

Nursing educator preparedness is another major issue that requires attention. Although key informants acknowledged the constraints within which nursing educators have to work, they were critical of the quality of the nurse educators. The views of key informants are borne out by the situation analysis of the 5-year Strategic Plan for Nursing Education, Training and Practice, which noted problems of insufficient numbers, heavy workloads, inadequate continuing professional development, outdated knowledge and skills, and an exodus from nursing education institutions due to the Occupation-Specific Dispensation, a public-sector financial incentive (45). Experience in other countries has shown that investment in nurse educators must accompany any major transformation in nursing education (11, 12, 31, 32). Although South Africa's Strategic Plan for Nursing Education, Training and Practice recommended the development of a national framework on nurse educators with dedicated resources (45), implementation has lagged behind, and many of the deadlines in the plan have passed already.

Key informants were of the opinion that the recruitment and selection of entrants to professional nursing accounted for some of the problems experienced in nursing. Another South African study also found that, although there are many more applicants to nursing courses than could be accommodated, they either did not meet the admission criteria, or nursing was the second or third choice (48). Nonetheless, the problems around student recruitment and selection to ensure that the best people enter the nursing profession are not unique to South Africa (21, 25–28, 37, 56).

The study findings suggest that nursing education in South Africa is grappling with getting the basics right for many of the building blocks considered essential for transformative education, with social accountability as a key feature. This paper makes an important contribution to the debate on issues that need to be addressed in the implementation of the impending nursing education reforms in South Africa, from the perspective of key opinion leaders in nursing and in the health sector. However, the findings are not generalisable, as the key informants were selected purposively. The information also represents the perceptions of key informants at a particular point in time, and hence we do not know how these perceptions have changed since the interviews were conducted in 2009. Nonetheless, it is one of the first studies using the WHO building blocks on transformative education and exploring the notion of social accountability as it relates to nursing education.

What then are the implications of the study findings for the implementation of the impending nursing education reforms in South Africa? The delays in the implementation of the proposed nursing education reforms provide a window of opportunity to incorporate the social accountability principles of responsiveness, relevance, quality, cost-effectiveness, and impact (9, 40, 42) into plans for implementation. Incorporating these principles requires stewardship and leadership from both the SANC and the Department of Health. The membership of SANC makes provision for community representatives, and the mandate of the Council is to protect the interests of the public. The SANC has the opportunity to embark on creative strategies to enhance community participation and increase public accountability.

The appointment of a Chief Nursing Officer at the beginning of 2014 is encouraging. We recommend that this incumbent draws on the skills and expertise of the nursing education structures, the national nursing association, student associations, and the many nurses who are keen to contribute to the current nursing education reform processes.

A great deal of research has been done on various factors relating to student selection, such as academic and non-academic predictors of success among student nurses (57), motives for entering nursing (35), and the role of gender (36). The selection criteria and system for student nurses into higher education should be based on the evidence already available at a global level, and efforts should be made to deal with its implementation considering the legal, sociocultural, political, and economic issues that will affect any decision taken.

Accountability for teaching and learning has to lie predominantly with the nursing education institutions themselves (43). The immediate way of institutionalising social accountability in the new system of nursing education is to ensure both that the curriculum is responsive to the needs of the community and that students are technically competent and graduate with the values and ethos of socially accountable graduates. Redman et al. addressed this issue many years ago by suggesting that a practice-oriented model of curriculum and competency assessment would address accountability requirements (37).

Programme assessment is equally important, as it provides evidence of educational effectiveness and therefore meets the demand for accountability (58). A third aspect of evaluation to improve accountability is raised by Thompson (59), who advocates widening the evaluation of individual faculty members to encompass assessment of the collective performance of the staff in a nursing education institution. This is because an individual lecturer cannot ensure that students complete the course as competent practitioners (59).

## Conclusion

Transformative education of nurses is critical in the move towards universal health coverage. Social accountability, which is an essential component of transformative education, necessitates that attention be paid to the issues of governance, curricula, nurse educator preparedness, student recruitment, and selection raised by key informants. Nursing education in South Africa is about to embark on profound changes with the move to the higher education sector. This transition provides a window of opportunity to address some of the issues that have troubled nursing education in the past, while embracing the concept of social accountability.

## References

[1] WHO (2010). Strategic directions for strengthening nursing and midwifery services 2011–2015.

[2] WHO AFRO (2007). WHO guidelines for implementing strategic directions for strengthening nursing and midwifery services in the African region 2007–2017.

[3] Global Health Workforce Alliance, WHO (2013). A universal truth: no health without a workforce.

[4] Institute of Medicine (2011). The future of nursing: leading change, advancing health.

[5] Frenk J, Chen L, Bhutta ZA, Cohen J, Crisp N, Evans T (2010). Health professionals for a new century: transforming education to strengthen health systems in an interdependent world. Lancet.

[6] Mullan F, Frehywot S, Omaswa F, Buch E, Chen C, Greyseen SR (2011). Medical schools in sub-Saharan Africa. Lancet.

[7] WHO, Global Health Workforce Alliance (2008). Scaling up, saving lives: task force for scaling up education and training for health workers.

[8] WHO (2013). Transforming and scaling up health professionals’ education and training. World Health Organization guidelines.

[9] Boelen C, Heck JE (1995). Defining and measuring the social accountability of medical schools.

[10] WHO (2014). Transforming the health workforce in support of universal health coverage: global toolkit for evaluating health workforce education.

[11] Spitzer A, Perrenoud B (2006). Reforms in nursing education across Western Europe: implementation processes and current status. J Prof Nurs.

[12] Spitzer A, Perrenoud B (2006). Reforms in nursing education across Western Europe: from agenda to practice. J Prof Nurs.

[13] Lane SH, Kohlenberg E (2010). The future of baccalaureate degrees for nurses. Nurs Forum.

[14] Wade GH (1999). Professional nurse autonomy: concept analysis and application to nursing education. J Adv Nurs.

[15] Keogh J (1997). Professionalization of nursing: development, difficulties and solutions. J Adv Nurs.

[16] Aiken L, Clarke S, Cheung R, Sloane D, Silber J (2003). Educational levels of hospital nurses and surgical patient mortality. JAMA.

[17] Blaney DR (1986). An historical review of positions in baccalaureate education in nursing as basic preparation for professional nursing practice 1960–1984. J Nurs Educ.

[18] Dlamini DM, Mashaba TG (1988). The changing policy on the basic nursing education system in South Africa and in its independent and self-governing states. Nurse Educ Today.

[19] Ehlers VJ (2002). Nursing education in the Republic of South Africa. Nurse Educ.

[20] Forbes MO, Hickey MT (2009). Curriculum reform in baccalaureate nursing education: review of the literature. Int J Nurs Educ Scholarsh.

[21] Hegarty J, Walsh E, Condon C, Sweeney J (2009). The undergraduate education of nurses: looking to the future. Int J Nurs Educ Scholarsh.

[22] Uys LR (1989). Nursing education in South Africa. Nurse Educ Today.

[23] Callery P (1990). Moral learning in nursing education: a discussion of the usefulness of cognitive-developmental and social learning theories. J Adv Nurs.

[24] Aboriginal Nurses Association of Canada, Canadian Association of Schools of Nursing, Canadian Nurses Association (2009). Cultural competence and cultural safety in nursing education.

[25] Bartlett HP, Simonite V, Westcott E, Taylor HR (2000). A comparison of the nursing competence of graduates and diplomates from UK nursing programmes. J Clin Nurs.

[26] Clinton M, Murrells T, Robinson S (2005). Assessing competency in nursing: a comparison of nurses prepared through degree and diploma programmes. J Clin Nurs.

[27] Pardue SF (1987). Decision-making skills and critical thinking ability among associate degree, diploma, baccalaureate, and master's-prepared nurses. J Nurs Educ.

[28] Takase M, Nakayoshi Y, Yamamoto M, Teraoka S, Imai T (2014). Competence development as perceived by degree and non-degree graduates in Japan: a longitudinal study. Nurse Educ Today.

[29] Manias E, Bullock S (2002). The educational preparation of undergraduate nursing students in pharmacology: clinical nurses’ perceptions and experiences of graduate nurses’ medication knowledge. Int J Nurs Stud.

[30] Rand ML, Pajarillo EJY (2015). A robust social and professional connection between master educator and doctor of nursing practice student instructor: virtual mentoring and preceptorship via distance education. Nurs Educ Today.

[31] Young L, Frost LJ, Bigl J, Clauson M, McRae C, Scarborough KS (2010). Nurse Educator Pathway Project: a competency-based intersectoral curriculum. Int J Nurs Educ Scholarsh.

[32] Tanner CA (2008). The Oregon Consortium for nursing education: a response to the nursing shortage. Policy Polit Nurs Pract.

[33] D'Souza MS, Karkada SN, Parahoo K, Venkatesaperumal R (2015). Perception of and satisfaction with the clinical learning environment among nursing students. Nurse Educ Today.

[34] O'Donnell H (2011). Expectations and voluntary attrition in nursing students. Nurse Educ Prac.

[35] McLaughlin K, Moutray M, Moore C (2010). Career motivation in nursing students and the perceived influence of significant others. J Adv Nurs.

[36] McLaughlin K, Muldoon OT, Moutray M (2010). Gender, gender roles and completion of nursing education: a longitudinal study. Nurs Educ Today.

[37] Redman RW, Lenburg CB, Hinton WP (1999). Competency assessment: methods for development and implementation in nursing education. Online J Issues Nurs.

[38] Sabin M, Taylor R, Tilley C (2010). Untangling a complex issue: an overview of initiatives to support nursing and midwifery student recruitment, selection and retention in Scottish Universities. Nurs Educ Today.

[39] (2010). Global consensus on social accountability of medical schools. http://www.healthsocialaccountability.org.

[40] Boelen C, Woollard B (2009). Social accountability and accreditation: a new frontier for educational institutions. Med Educ.

[41] Woollard RF (2006). Caring for a common future: medical schools’ social accountability. Med Educ.

[42] Boelen C, Woollard R (2011). Social accountability: the extra leap to excellence for educational institutions. Med Teach.

[43] Celletti F, Reynolds TA, Wright A, Stoertz A, Dayrit M (2011). Educating a new generation of doctors to improve the health of populations in low- and middle-income countries. PLoS Med.

[44] WHO Global standards for the initial education of professional nurses and midwives.

[45] DOH (2013). Strategic Plan for Nursing Education, Training and Practice 2012/13–2016/17.

[46] Republic of South Africa (2005). Nursing Act No. 33 of 2005.

[47] Blaauw D, Ditlopo P, Rispel LC (2014). Nursing education reform in South Africa-lessons from a policy analysis study. Glob Health Action.

[48] Breier M, Wildschut A, Mgqolozana T (2009). Nursing in a new era: the profession and education of nurses in South Africa.

[49] Rispel LC, Blaauw D, Chirwa T, de Wet K (2014). Factors influencing agency nursing and moonlighting among nurses in South Africa. Glob Health Action.

[50] Rispel LC, Chirwa T, Blaauw D (2014). Does moonlighting influence South African nurses’ intention to leave their primary jobs?. Glob Health Action.

[51] Brooks J, McCluskey S, Turley E, King N (2015). The utility of template analysis in qualitative psychology research. Qual Res Psychol.

[52] DOH (2006). National Human Resource Plan.

[53] DOH (2008). Nursing Strategy for South Africa.

[54] DOH (2011). Human Resources for Health South Africa: HRH strategy for the health sector 2012/13–2016/17.

[55] WHO (2010). Increasing access to health workers in remote and rural areas through improved retention.

[56] Rich KL, Nugent KE (2010). A United States perspective on the challenges in nursing education. Nurse Educ Today.

[57] Ali PA, Naylor PB (2010). Association between academic and non-academic variables and academic success of diploma nursing students in Pakistan. Nurse Educ Today.

[58] Oermann MH, Gaberson KB (2009). Evaluation and testing in nursing education.

[59] Thompson CM (2011). Transitioning from a faculty to an administrative role: part 1, moving from individual to collective accountability. Nurse Educ.

# Sub article: Nursing education reform in South Africa – lessons from a policy analysis study

**DOI:** 10.3402/gha.v7.26401

**Published:** 2014-12-22

**Authors:** Duane Blaauw, Prudence Ditlopo, Laetitia C. Rispel

**Affiliations:** Centre for Health Policy & Medical Research Council Health Policy Research Group, School of Public Health, Faculty of Health Sciences, University of the Witwatersrand, Johannesburg, South Africa

**Keywords:** nursing, nursing education, professionalisation, policy analysis, education reform, South Africa

## Abstract

**Background:**

Nursing education reform is identified as an important strategy for enhancing health workforce performance, and thereby improving the functioning of health systems. Globally, a predominant trend in such reform is towards greater professionalisation and university-based education. Related nursing education reform in South Africa culminated in a new Framework for Nursing Qualifications in 2013.

**Objective:**

We undertook a policy analysis study of the development of the new Nursing Qualifications Framework in South Africa.

**Methods:**

We used a policy analysis framework derived from Walt and Gilson that interrogated the context, content, actors, and processes of policy development and implementation. Following informed consent, in-depth interviews were conducted with 28 key informants from national and provincial government; the South African Nursing Council; the national nursing association; nursing academics, managers, and educators; and other nursing organisations. The interviews were complemented with a review of relevant legislation and policy documents. Documents and interview transcripts were coded thematically and analysed using Atlas-ti software.

**Results:**

The revision of nursing qualifications was part of the post-apartheid transformation of nursing, but was also influenced by changes in the education sector. The policy process took more than 10 years to complete and the final Regulations were promulgated in 2013. The two most important changes are the requirement for a baccalaureate degree to qualify as a professional nurse and abolishing the enrolled nurse with 2 years of training in favour of a staff nurse with a 3-year college diploma. Respondents criticised slow progress, weak governance by the Nursing Council and the Department of Health, limited planning for implementation, and the inappropriateness of the proposals for South Africa.

**Conclusion:**

The study found significant weaknesses in the policy capacity of the main institutions responsible for the leadership and governance of nursing in South Africa, which will need to be addressed if important nursing education reforms are to be realised.

There is global recognition of the urgent need to scale up educational programmes for the production of more health professionals in order to address patient and population health priorities and improve health system performance (1–4). Around the world, nurses are the largest category of health care providers (5), and play a vital role in health sector reform initiatives (6). Given the centrality of nurses in the health care system, changes in the production, Scope of Practice, and education of the nurse workforce are essential strategies for effecting improvements in the functioning and impact of health care systems (1).

Globally, the predominant trend in nursing education over the last century has been towards greater professionalisation through the lengthening of training periods, and the shift from a hospital-based apprenticeship model to professional education in institutions of higher learning (7, 8). Nevertheless, many countries still allow multiple pathways to registration as a professional nurse, generally with 3 or 4 years of higher education, and obtaining either a nursing college diploma or a university degree (9).

In recent years, an important development in the professionalisation of nursing education has been the call for a baccalaureate degree as the minimum requirement for entry to the profession (10). Proponents have argued that a university degree is required to cope with the increasing complexity of contemporary nursing practice resulting from a combination of factors including changes in patient and disease profiles, advances in medical and information technology, the shift to evidence-based practice, the need for life-long professional development, the challenges of working in health care teams, and the demands of ongoing health system reforms (3, 11–13). However, the shift to a baccalaureate degree as entry to nursing practice is also influenced by the desire to enhance the professional status of nurses, attract high-quality students, escape medical domination, and allow for more autonomous nursing practice (14, 15).

In South Africa, increasing professionalisation and a shift to university education have been important features of the reform of nursing education (16, 17). The first nursing school was established in 1877, following the standard hospital apprenticeship model of the time, and despite the efforts of its founder, it was placed under the jurisdiction of the Medical Council, instead of the Department of Education (18). The establishment of the South African Nursing Council (SANC) in 1944 wrested control of nursing education from the medical profession (19). However, a 3-year diploma at a hospital-based nursing college remained the only pathway to qualifying as a registered nurse (20). The first university nursing degree programmes in the country were introduced in 1956 but uptake remained relatively small (21). A more significant policy shift occurred in 1986 when all nursing colleges were required to become affiliated with university-based nursing departments, which placed them officially within the higher education system (16). At the same time, a new comprehensive 4-year curriculum (including general nursing, midwifery, community nursing, and psychiatric nursing) was introduced for the training of professional nurses in South Africa, which could be completed through a nursing college diploma or a university degree (22).

Since South Africa's democracy in 1994, there has been a renewed focus on nursing education as part of the post-apartheid transformation of both the health and higher education sectors (23). The nursing education policy reforms have included the rationalisation of nurse training institutions, changing the Scope of Practice of nurses, and revising nursing qualifications (22–25). The revision of nursing qualifications has been driven by changes within the profession and the imperative to align nursing qualifications with the new National Qualifications Framework (NQF) – a comprehensive system for the classification and articulation of qualifications in the country (26). A key recommendation of the new nursing qualifications proposals is that registration as a professional nurse will require completion of a baccalaureate degree in nursing, rather than a nursing college diploma.

In light of South Africa's health care reforms to achieve universal health coverage (27) and the importance of nursing education in the preparation of nurses for their roles in leading and implementing these reforms, this study analysed the context, content, actors, and process of the development of the new Framework for Nursing Qualifications in South Africa.

## Methods

### Conceptual framework

Contemporary policy analysis is based on the premise that policy-making is inherently political in nature and seeks to provide a systematic method for analysing policy processes and the factors that influence them (28). Although a number of frameworks and tools have been developed for health policy analysis (28–31), this study used an analytical framework derived from Walt and Gilson (30) that interrogates the context, process, content, and actors of policy development and implementation. Context refers to the broader situational and structural factors influencing the reform. The process analysis investigates the way in which policies are identified, formulated, and implemented; the timing of events; and the strategies used at each stage. The analysis of content focuses on the nature and details of the policy proposals. The study of actors is concerned with the key stakeholders involved in developing and implementing the reforms, as well as their differing roles, values, interests, and influence (30).

### Policy of focus

Although there have been a number of reforms in nursing education in South Africa since 1994 (23), this study analysed the context, process, content, and actors involved in the development of the new Nursing Qualifications Framework in South Africa. Essentially, this policy reform was concerned with defining the different categories of nurses in South Africa, specifying the minimum qualifications required for each category of nurse, and describing the articulation between them.

The study reported in this paper was part of a larger project to examine the dynamics, strengths, and weaknesses of nursing policy-making in South Africa (32). It constitutes one of four policies of focus identified at a broad consultative workshop held with key nursing stakeholders (33).

### Study sites

The focus of this study was primarily on national policy processes. Although actors at the national level may drive nursing policy development, the responsibility for implementation is with provincial managers and the ultimate impact of policies is on front-line nurses. Therefore, the project collected data from all three levels. Sub-national data collection focused on four provinces: Gauteng, Eastern Cape, Free State, and Western Cape.

### Data collection and study participants

The overall study design was an in-depth, qualitative, case study of the new Nursing Qualifications Framework in South Africa. Case study methodology allows for a detailed exploration of the complex dynamics and relationships within a real-life context (34). The researchers also sought to maximise participation and policy engagement with policy actors as part of the research process.

The research team reviewed relevant legislation, regulations, government and nursing council documents, and inputs made by nursing education stakeholder groups in order to understand the context and content of the new Nursing Qualifications Framework.

The research team conducted interviews with 28 purposively selected key informants, drawn from six categories: national and provincial government officials (*n*=14), nursing managers from the private sector and non-governmental organisations (*n*=5), nursing academics in colleges and universities (*n*=6), and representatives from the SANC and other nursing organisations (*n*=3).

Semi-structured face-to-face interviews were conducted in English after obtaining written informed consent. If the key informant (KI) agreed, the interviews were recorded to improve accuracy and to support more detailed analysis. The interviewer also kept detailed notes of each interview. The issues covered with each KI included: aims of the policy; policy design and content; contextual factors influencing policy development; important milestones in the policy process; the roles, interests, and influence of different actors; motivation for the strategies adopted; factors influencing the policy process; and key informants’ interpretation of the success or failure of the policy initiative.

### Data analysis

Documents, interview transcripts, and written notes were analysed using standard approaches to qualitative data analysis (35, 36). The analytical themes were derived from the specific research objectives, the conceptual framework of the study, and the data. The Atlas-ti software programme was used to support qualitative data analysis. The analysis of process included the careful documentation of key milestones, content development, and strategic shifts. The actor analysis included a formal stakeholder analysis (31).

### Ethical considerations

National and international ethical standards were followed throughout the research. The University of the Witwatersrand Human Research Ethics Committee (Medical), and the relevant provincial health authorities granted permission to conduct the study.

Respondents were provided with a study information sheet explaining the purpose of the study and the terms of their consent. Those who agreed to be interviewed were asked to sign the study consent form. Separate consent was also obtained for any recording of interviews. Personal identifiers were removed from all transcripts and results.

## Results

The context, process, content, and actors relevant to the development of the new Nursing Qualifications Framework are described briefly, followed by respondents’ main criticisms of the policy process.

### Context

Table 1 presents a timeline of key events in the development of the new Nursing Qualifications Framework, as well as significant contextual developments in the broader nursing and education sectors.

**Table 1:** Timeline of key events 1994–2013: new Nursing Qualifications Framework (see text for abbreviations).

Year	General Health/Nursing Sector	Nursing Qualification Framework	Education Sector
1978	Nursing Act No. 50		
1986		4 year comprehensive course introduced (R425)	
1995			SAQA Act No. 58 establishes NQF with 8 bands
1997	White Paper for the Transformation of the Health System		White Paper for the Transformation of Higher Education Higher Education Act No. 101
1998			Further Education and Training Act No. 98
1999	Rationalisation (closing) of nursing colleges National Summit on Nursing, Kopanong		
2000	Pick report on Human Resources for Health		
2001		Nursing SGB established	General and Further Education and Training Quality Assurance Act No. 58
2003	National Health Act No. 61	Nursing SGB releases draft NQF qualifications for public comment	
2004		Nursing SGB finalises qualifications for registration on NQF	
2005	Nursing Act No. 33		
2006	New DDG appointed for Human Resources in NDOH Human Resources Strategic Framework		Further Education and Training Colleges Act No. 16
2007	Sections of Nursing Act promulgated but awaiting required Regulations	SANC revises qualifications for registration on NQF	Review of SAQA and NQF completed New Higher Education Qualifications Framework (HEQF) with 10 bands to be implemented by 1 January 2009
2008	Nursing Strategy		National Qualifications Framework Act No. 67
2009	New Minister of Health, Dr Motsoaledi, appointed	SANC sets deadline of 30 June 2010 for phasing out of legacy qualifications	
2010	New Director-General, Ms Matsoso, appointed	Nursing education stakeholders develop proposals for HEQF qualifications Legacy qualifications extended to 30 June 2012 NDOH commissions independent audit of all training institutions but report not released	
2011	Nursing Summit and Compact, April Revised Human Resources for Health Plan, October Ministerial Task Team on Nursing Education and Training established Acting Chief Nurse appointed in NDoH. Official post advertised	Qualifications framework debated at Nursing Summit Legacy qualifications extended to 30 June 2013 Draft Regulations released for public comment on 14 Dec 2011	
2012		Legacy qualifications extended to 30 June 2015	
2013		SANC task team finalised new qualifications to be submitted to SAQA 8 March: New qualification Regulations gazetted Strategic Plan for Nursing Education, Training and Practice released New programmes to be submitted for accreditation by CHE and SANC	

**Fig. 1:** Changes in the Nursing Qualifications Framework.

KIs argued that the revision of nursing qualifications was an important part of the on-going transformation of nursing in South Africa, and not only a response to changes in the higher education sector (Table 1).

The development of the new qualifications is about updating, modernising the practice of nursing. [KI 2, National Department of Health]

For the profession we need a professional nurse that's a graduate nurse that is in line with the rest of the world and the new qualifications would give us that. [KI 12, Nursing Academic]

The post-apartheid transformation of nursing legislation culminated in the passing of a new Nursing Act in 2005. Although the Act was promulgated in 2007, many provisions of the Act required further specification in Regulations. KIs pointed out that an important contextual factor was the development of draft Regulations outlining the Scopes of Practice of different categories of nurses and the qualifications required for each category. Nursing stakeholders were dissatisfied with the Scope of Practice Regulations and qualifications inherited from the previous Nursing Act of 1978 and had strongly advocated for an overhaul of these provisions. KIs explained that the main motivations for education reform included: insufficient distinction in the roles of different nursing categories; the need to address changing disease patterns and health system priorities; and the need to reflect the more independent practice of contemporary nurses. Others saw it as an opportunity to review the 4-year comprehensive nursing course introduced in 1986 which paradoxically had been criticised both for excessive training in all four areas of care, and for inadequate preparation of graduates for competent practice in all of these areas, particularly midwifery.

We have got a challenge of high maternal and child mortality. When you ask people what could be the cause of that, they will then begin to look at this 4-year programme and ask whether it produces competent midwives or not? [KI 18, Nursing Association]

The post-apartheid era also brought broader challenges for nursing that needed to be considered in the revision of the nursing qualifications. These included increased service demands, easier international migration, staff shortages, decline in the image and status of the profession, difficulties in attracting good recruits, an ageing workforce, and generally low staff morale (22, 24).

Another critical contextual factor was the development of a new NQF by the Department of Education. Prior to 1995, the SANC was the main regulator of nursing training and qualifications but the introduction of the South African Qualifications Authority (SAQA) and the new NQF meant that nursing qualifications now had to comply with broader Department of Education policy.

A 2007 review of SAQA and the NQF resulted in a significant revision of the NQF to develop the new Higher Education Qualifications Framework (HEQF). The revised NQF consists of ten levels, instead of the previous eight, with each level providing a broad indication of the types of learning outcomes and assessment criteria that are appropriate to a qualification at that level (26). KIs explained that the new nursing qualifications had to keep up with this changing national context.

### Process

The timeline in Table 1 shows that the new Nursing Qualifications Framework policy process has taken more than 10 years to complete. The related work on the Scope of Practice Regulations has taken even longer since the initiative began in 1997 and was not yet finalised by 2013.

The reform of nursing education and training can be divided into two main phases.

The first phase of the reform process occurred between 2001 and 2009 and was primarily concerned with aligning existing nursing qualifications with the NQF. These were the so-called legacy qualifications inherited in 1994 for the training of enrolled nursing auxiliaries (1 year training), enrolled nurses (2 years of training), and diploma- or degree-qualified professional nurses with 4 years of training. SAQA had defined specific processes for this that involved the establishment of a Nursing Standards Generating Body (SGB). The Nursing SGB produced various draft qualifications between 2002 and 2004 but the process stalled because of lack of resources. A further difficulty was the change from the NQF to the HEQF, which meant that the work done so far had to be revised. The SANC took over finalising the registration of the legacy qualifications with SAQA, eventually completing the task in 2009.

The second phase of reform from 2008 to 2013 dealt with the development of a completely new Nursing Qualifications Framework, also aligned with the HEQF which was meant to be led by SANC. Timeframes were not adhered to, and despite an initial deadline of 30 June 2010 being set for the phasing out of the legacy qualifications, the process has continually been extended. The new deadline, at the time of writing, is the 30 June 2015.

Although the involvement of nurse educator groups resulted in some increased momentum, respondents argued that progress has been sporadic and the process laboured. In April 2011, a national Nursing Summit was held where the following statement regarding the new Nursing Qualifications Framework was made (37):

We call upon SANC in collaboration with the National Department of Health, CHE [Council for Higher Education] and SAQA to fast-track the processing and implementation of the new Nursing Qualifications’ Framework and appropriate transitional arrangements.

Following the summit, the Minister of Health appointed a Ministerial Task Team on Nursing Education and Training which finally provided the impetus to complete this phase of the policy process. Regulations for the new nursing academic qualifications were released for public comment in December 2011 and finally promulgated in March 2013. The Minister of Health also released the new Strategic Plan for Nursing Education, Training, and Practice in March 2013 (25). However, both SAQA and the Council for Higher Education (CHE) have to approve the new qualifications and teaching programmes prior to their implementation in 2015.

### Content

Figure 1 provides a graphic representation comparing the pre-1994 qualifications and the final new Qualifications Framework as outlined in the 2013 Regulations. Since 1985 the main nursing qualifications recognised in South Africa were as follows (22, 23):

1. Enrolled nursing auxiliary: 1-year certificate at a training hospital;
2. Enrolled nurse: 2-year certificate at nursing college;
3. Professional nurse: 4-year diploma at nursing college or 4-year baccalaureate degree at university.

**Fig. 2:** Mapping of key actors involved in the Nursing Qualifications Framework policy process.

A bridging programme also made it possible for enrolled nurses to complete a 2-year diploma at a nursing college and qualify as a registered general nurse. Specialised nurses completed either a postgraduate diploma or master's degree, and doctoral nursing programmes were available at universities.

The new Qualifications Framework includes two fundamental changes (Fig. 1). First, registration as a professional nurse will now require completion of a 4-year baccalaureate degree at a university. This is in keeping with trends in high-income countries, and satisfied some nursing educators concerned about the declining professional status of nurses in South Africa. The bridging course has also been withdrawn so there is now only one pathway to becoming a professional nurse instead of three. Second, the enrolled nurse has been replaced by a staff nurse with a 3-year college diploma at HEQF level 6. This was mainly because a 2-year diploma is not permissible on the revised HEQF. But respondents also argued that a reconfigured 3-year staff nurse qualification would be a significant step towards meeting South Africa's basic nursing needs.

I understand that the needs of the country are about an expanded scope of practice for an enrolled nurse. [KI 26, Nursing Academic]

The Scope of Practice of the new staff nurse has been broadened so that they will be able to work more independently and provide basic nursing care to uncomplicated patients in all settings.

### Actors

The key actors involved in the nursing qualifications policy process are represented in Fig. 2.

Overall governance and leadership of the nursing sector is shared between the SANC and the Human Resources Division of the National Department of Health (NDoH). Other important actors from the health sector included nursing educators, nursing practitioners, nursing organisations and the national nursing association, and health sector unions. Nursing educators include individual training institutions, as well as educator groupings such as the Forum of University Nursing Deans in South Africa (FUNDISA), the Nurse Educators Association (NEA), the College Principals and Academic Staff (CPAS) forum, and the Private Health Education Providers of South Africa (PHEPSA) (Fig. 2) representing university, nursing college, and private educators. A significant development highlighted by this policy case study is the increasing role of actors from the education sector, including the Department of Higher Education and Training, SAQA, and the CHE (Fig. 2), in nursing education and training.

Various actors have played a central role at different stages of the process. Phase 1 of the process was initially driven by the Nursing SGB, a sub-structure of SAQA, intended to ensure broader representation in the development of national qualifications.

In 2001, there was the Nursing SGB which played a great role in developing the Nursing Standards. The very new qualifications that we are talking about now, they were given birth to by the Nursing SGB. [KI 16, Nursing Educator.]

Not surprisingly, the SGB came into conflict with SANC, which had previously been the only actor responsible for the regulation and development of nursing qualifications. Ultimately, the SANC reasserted its role and the Nursing SGB was abandoned.

Nursing educators dominated the phase 2 process. The so-called Nursing Education Stakeholders (NES), a group representing all nurse educator organisations, developed the initial proposals and advocated and lobbied extensively for the new Nursing Qualifications Framework. A workshop held by the NES in February 2010 defined the subsequent development of the debate and they played a prominent role in the presentations and discussions on the Qualifications Framework at the Nursing Summit in April 2011. Despite initial differences within the NES group, particularly from PHEPSA and CPAS, the NEA-FUNDISA proposals came to be accepted and disseminated.

One of the success stories that FUNDISA has played a critical part in is coming up with a Nursing Qualification Framework that SANC has now adopted. [KI 26, Nursing Academic]

The finalisation of the Regulations was subsequently taken forward by the Ministerial Task Team on Nursing Education and Training. The influence of the NDoH, particularly the Human Resource Division, has been uneven. At an earlier stage some officials appeared uncomfortable with the shift to greater professionalisation through the baccalaureate degree.

Our Minister at one point said, ‘the way that South Africa is going with its academic qualifications, very soon we will be saying that Agricultural Extension Officers must train at university, and the country will be without food because how can someone who comes from the university know how to farm, how to till the soil.’ That is the way it is. [KI 39, National Department of Health]

Nevertheless, the Minister of Health signed the final Regulations, including the requirement of a baccalaureate degree for professional nurses.

### Stakeholder criticisms of the policy process

Table 2 summarises key informants’ main criticisms of the nursing qualifications policy process.

**Table 2:** Key criticisms of new nursing qualifications policy process

Context issues	• Context determined by education sector rather than nursing. • Process unable to keep up with changing context.

Process issues	• Policy progress took too long. • Limited planning for actual implementation.
Content issues	• Nursing colleges have to be registered as higher education institutions. • Limited evidence base for proposed revisions. • Uncertainty whether proposals match health needs of the country. • Debates about direct-entry midwifery.
Actor issues	• Weak leadership and governance by SANC and NDoH. • Domination by nurse educators with limited involvement of employers and front-line nurses. • Limited real engagement with NDoH.

With regard to contextual factors, SANC and nursing stakeholders have grappled with the introduction of the Department of Education into the regulation of nursing education.

I mean the Council has always had a Qualifications Framework, but it belonged to the Council and now it had to start merging with the education system of the country. [KI 9, Nursing Educator]

Some informants also highlighted the difficulties of dealing with the ongoing changes in the education sector.

We were busy writing those nursing qualifications of the previous Council and then they got them complete and ready to implement and then they realised the NQF had changed. [KI 25, Nursing Manager]

Not surprisingly, in terms of process, a major concern has been the very slow pace of policy development, particularly in relation to producing the enabling Regulations required by the new Nursing Act.

Although the ground work has been done, the implementation is completely at a standstill. There is absolutely no implementation in any aspect of the Nursing Act or any of those draft Regulations that are there. [KI 24, Former SANC Member]

Some respondents were also concerned about the limited planning for the implementation of the new qualifications, which will have a significant impact on nursing education, workforce planning, and existing nurses. Education institutions still need to develop and implement new curricula and training programmes, for example. A more fundamental problem is that it is envisaged that the staff nurse diploma will be provided by nursing colleges, but HEQF level 6 is in the Higher Education band and most public and private nursing colleges are not currently registered as higher educational institutions – and the process for changing this is neither quick nor simple.

A few interviewees questioned the evidence-base for the content of the policy recommendations:

So for me I am not very sure if enough research was done in terms of making a point that the new qualifications are actually tailor-made to suit the current demands of the country. [KI 14, Manager, Free State Department of Health]

This echoed concerns about whether the current educational proposals meet South Africa's health needs. Some respondents were of the opinion that the tendency towards professionalisation reflected a preoccupation with international comparability, status, and earning potential rather than local health priorities.

The case study revealed a number of problems with key policy actors. The policy process stalled between SANC and the NDoH, but it was unclear which organisation was primarily responsible for the delay. SANC blamed the NDoH:

And why they [Regulations] were not published, I think there were challenges in the Department of Health. [KI 24, Former SANC Member]

At the same time, the NDoH blamed the SANC:

It's a matter of just passing them I think it has been more the Council problems that have stood in the way of those Regulations. [KI 1, National Department of Health]

Other interviewees highlighted weaknesses of both of these key actors in relation to governance of the nursing sector. They were blamed for the poor coordination between them and their failure to provide leadership to complete the policy process:

I really think that it seems as if one body waits for the other instead of there being somebody that drives the process and then takes it through to completion. [KI 23, Nursing Manager]

With regard to SANC, problems with the appointment of a registrar, internal divisions, weak administration, changes in the SANC board, and the subordinate role of the new Council in relation to the Minister were blamed for the slow progress:

It's a dysfunctional body I have to say. The sad thing is that it has been going on for years; it's not just this Council. When I was on the Council both terms were relatively dysfunctional. [KI 7, Manager, Gauteng Department of Health]

The NDoH also came in for criticism due to their limited capacity in driving the policy reform processes, and the fact that there was no dedicated unit or senior manager responsible for nursing issues. Some respondents felt that the NDoH had not been sufficiently involved in the planning of the new nursing qualifications.

Finally, other actors complained about being side-lined in the qualification framework process, and argued that there had been inadequate consultation on an issue that would have significant long-term consequences for all nurses in South Africa:

In that discussion the unions and the practitioners have not been involved. It's really been agreed by the Nursing Council and universities. [KI 19, Nursing Association]

## Discussion

This is one of the first policy analysis studies in South Africa concentrating exclusively on policy development and implementation in the nursing sector. This case study of the development of a new Nursing Qualifications Framework indicates significant weaknesses in the political and technical capacity of the main institutions that are supposed to provide leadership and governance for policy-making in the nursing sector in South Africa.

The final Nursing Qualifications Framework developed for South Africa, in proposing both a baccalaureate degree for professional nurses and new general staff nurses, is an interesting compromise that follows the global trend towards more professionalisation, but also attempts to address nursing shortages and the significant deficit in basic nursing care in the country.

A few high-income countries, including Belgium, Italy, Spain, Norway, Canada, the United Kingdom, Australia, and New Zealand, have recently made the baccalaureate degree a requirement for registration (8, 38–40). Similar policies are being considered in other high-income countries. In the United States, for example, the American Nursing Association has officially supported baccalaureate entry to practice since 1965 (41, 42), and the recent influential Institute of Medicine monograph on the future of nursing recommended that baccalaureate nurses should make up 80% of the total nursing workforce by 2020 (3). However, discourses regarding nursing education reform in low- and middle-income countries are different. The emphasis is more on educational changes required to address the global shortage, maldistribution, retention, skills mix, Scope of Practice, and performance of nurses so as to strengthen health systems, scale up priority health interventions and achieve universal coverage (43–46). For example, the recent World Health Organization (WHO) report on global standards for the initial education of professional nurses and midwives makes reference to the global shift towards university-based nursing education but identifies this as a goal for the future (47). Nevertheless, baccalaureate entry to practice reforms have also been implemented in a few middle-income countries, such as the Philippines and Brazil, and are being considered in others, including Mexico, India, and Jordan (9, 48).

The influence of nursing educators in the South African policy process contributed to acceptance of the baccalaureate degree requirement for professional nurses, despite initial concerns from NDoH officials that this was not appropriate or cost-effective for South Africa (23). Although it is frequently stated that university-trained nurses are more capable of dealing with the demands of contemporary nursing, and a number of countries have made this a requirement for practice, the empirical evidence comparing the competence of degree- and diploma-trained professional nurses is more mixed (49–53). In addition, some commentators have questioned whether the nursing degree requirements recently introduced in Canada and Australia are delivering everything they promised (39, 40).

There are some limitations of this policy analysis case study. Although many of the key stakeholders involved in the policy process were interviewed, it was sometimes difficult to cover all of the issues within the time constraints of a single interview. Another difficulty was KI recall bias and the reliance on a retrospective report of events, some of which happened many years previously. Where possible, we corroborated accounts between different respondents and compared the interviews with the documentary evidence. A further limitation is that the analysis focuses mainly on the policy development phase – policy implementation has barely begun and is likely to present additional challenges. Nonetheless, the policy analysis approach of this case study, and its focus on broader systems of governance and leadership within nursing, are unusual in the nursing literature. Most of the available international literature linking nursing and policy-making is concerned with the absence of nurses in broader health policy processes (54–58). There is also a fairly large body of work on the professional regulation of nurses (59, 60), but this literature has a mostly technical focus and has seldom drawn explicitly on approaches from the political and policy sciences.

Reports outlining the most urgent priorities for nursing education reform are frequent (3, 61–63). However, more analysis is required of the leadership, institutional capacity, and policy processes required to implement such reforms in different countries. The nursing education literature has also focused mostly on the technical aspects of nursing education reform – changes in qualifications, competencies, curricula, and pedagogy, for example – but commented little on the politics and processes of nursing education reform. This paper identifies considerable failings in the processes of nursing policy development in South Africa. The analysis points to inadequate nursing policy-making expertise and leadership within SANC and the NDoH, as well as poor coordination between them, that is likely to undermine future policy development and implementation in nursing education. Certain actors, such as nursing educators and unions, appear to have some policy competence, but are unable to overcome the weaknesses within the SANC and the NDoH, who bear ultimate responsibility for nursing policy development and implementation.

A few other authors have commented on the capacities and processes required to support reform in nursing education, at the macro and meso levels. For example, the WHO guidelines on transformative scale-up of health professional education emphasise the importance of national leadership, governance, and planning of health education reform (4). Spitzer and Perrenoud (64) analysed the organisational capacities required to support nursing education reforms in Western Europe aimed at transferring nursing education into universities. Lastly, recent studies have highlighted the institutional weaknesses of many nursing and midwifery regulatory bodies in central, east, and southern Africa and their failure to enact the important educational and regulatory reforms needed to cope with new health system priorities (65, 66). Clearly, more research on this topic is necessary.

It is encouraging that nursing stakeholders eventually finalised a new Framework for Nursing Qualifications in South Africa and produced the Regulations required by the Nursing Act of 2005. Also, the Strategic Plan for Nursing Education, Training, and Practice released by the NDoH in March 2013 (25) thoroughly summarises the current challenges facing nurses in South Africa, provides a relatively comprehensive reform strategy, and details an implementation plan for achieving it (25). However, one weakness of the new strategic plan is that it fails to interrogate the limited success of previous policy initiatives in the nursing sector, including the previous Nursing Strategy of 2008 (24). Policy analysis, such as that presented in this paper, can provide useful insights to strengthen future policy development and implementation. The analysis presented in this paper suggests that the new plan is unlikely to be successful without significant interventions to improve the policy and implementation capacity of SANC and the human resource division of the NDoH. Although South Africa's first Chief Nursing Officer was appointed in January 2014, implementation of the plan is already falling behind its own strategic objectives and planned timelines. Furthermore, the new strategic plan process has been a missed opportunity to tackle the systemic and structural weaknesses in the policy machinery required to drive nursing reform in South Africa.

## Conclusion

The performance and health outcomes of the South African health system are disappointing given the country's level of economic development. There is renewed commitment and energy to addressing the problem and a number of health system reforms are currently underway (27, 67). However, a successful turnaround depends on improving the performance of nurses, the largest category of health providers. This, in turn, depends on the effective implementation of proposed changes in the education, training, and practice of nurses. This study suggests that there is sufficient expertise in the country to analyse the problems and make recommendations for educational reform. However, the study also shows that there is a fundamental and longstanding crisis in the institutional governance and leadership of the nursing sector in the country. The policy capacity of key institutions requires urgent strengthening if these important nursing education reforms are to be realised.

## References

[1] Frenk J, Chen L, Bhutta ZA, Cohen J, Crisp N, Evans T (2010). Health professionals for a new century: transforming education to strengthen health systems in an interdependent world. Lancet.

[2] Kinfu Y, Dal Poz MR, Mercer H, Evans DB (2009). The health worker shortage in Africa: are enough physicians and nurses being trained?. Bull World Health.

[3] Institute of Medicine (2011). The future of nursing: leading change, advancing health.

[4] WHO (2013). Transforming and scaling up health professionals’ education and training. World Health Organization guidelines.

[5] WHO (2006). World Health Report 2006. Working together for health.

[6] Global Health Workforce Alliance, WHO (2013). A universal truth: no health without a workforce.

[7] Hegarty J, Walsh E, Condon C, Sweeney J (2009). The undergraduate education of nurses: looking to the future. Int J Nurs Educ Scholarsh.

[8] Spitzer A, Perrenoud B (2006). Reforms in nursing education across Western Europe: implementation processes and current status. J Prof Nurs.

[9] Nichols BL, Davis CR, Richardson DR, Institute of Medicine (2010). International models of nursing. The future of nursing: leading change, advancing health.

[10] Forbes MO, Hickey MT (2009). Curriculum reform in baccalaureate nursing education: review of the literature. Int J Nurs Educ Scholarsh.

[11] WHO (1988). Nurses education in the Eastern Mediterranean region. Guidelines on future directions.

[12] WHO (2001). Nurses and midwives for health. WHO European strategy for nursing and midwifery education.

[13] Lane SH, Kohlenberg E (2010). The future of baccalaureate degrees for nurses. Nurs Forum.

[14] Keogh J (1997). Professionalization of nursing: development, difficulties and solutions. J Adv Nurs.

[15] Wade GH (1999). Professional nurse autonomy: concept analysis and application to nursing education. J Adv Nurs.

[16] Searle C (1983). New dimensions – nursing education in the post-secondary education system in the Republic of South Africa. Curationis.

[17] Rispel L, Schneider H (1991). Professionalization of South African nursing: who benefits?. Int J Health Serv.

[18] Dlamini DM, Mashaba TG (1988). The changing policy on the basic nursing education system in South Africa and in its independent and self-governing states. Nurse Educ Today.

[19] Marks S (1994). Divided sisterhood: race, class and gender in the South African nursing profession.

[20] Uys LR (1989). Nursing education in South Africa. Nurse Educ Today.

[21] Ehlers VJ (2002). Nursing education in the Republic of South Africa. Nurse Educ.

[22] Breier M, Wildschut A, Mgqolozana T (2009). Nursing in a new era. The profession and education of nurses in South Africa.

[23] Mekwa J, Ntuli A, Crisp N, Clarke E, Barron P (2000). Transformation in nursing education: chapter 13. South African health review 2000.

[24] Department of Health (2008). Nursing Strategy for South Africa 2008.

[25] Department of Health (2013). Strategic Plan for Nursing Education, Training and Practice. 2012/13–2016/17.

[26] South African Qualifications Authority (2012). Level descriptors for the South African National Qualifications Framework.

[27] Department of Health (2011). National Health Insurance in South Africa: policy paper. Government Notice: 657 of 12th August 2011, Gazette Number 34523.

[28] Reich M, Berman P (1995). The politics of health sector reform in developing countries: three cases of pharmaceutical policy. Health sector reform in developing countries: making development sustainable.

[29] Walt G (1994). Health policy: an introduction to process and power.

[30] Walt G, Gilson L (1994). Reforming the health sector in developing countries: the central role of policy analysis. Health Policy Plan.

[31] Varvasovszky Z, Brugha R (2000). Stakeholder analysis. Health Policy Plan.

[32] Ditlopo P, Blaauw D, Penn-Kekana L, Rispel LC (2014). Contestations and complexities of nurses’ participation in policy-making in South Africa. Glob Health Action.

[33] Rispel L (2008). Research on the State of Nursing (RESON). Exploring nursing policies, practice and management in South Africa.

[34] Yin RK (1994). Case study research: design and methods.

[35] Neuman W, Neuman W (1994). Analyzing qualitative data. Social research methods: qualitative and quantitative approaches.

[36] Miles M, Huberman A (1994). Qualitative data analysis: an expanded resource book.

[37] Department of Health (2011). Nursing compact: national nursing summit.

[38] Ali PA, Watson R (2011). The case for graduate entry to the United Kingdom nursing register. Int Nurs Rev.

[39] Pijl-Zieber EM, Grypma S, Barton S (2014). Baccalaureate nursing education: has it delivered? A retrospective critique. Nursing Leadersh (Toronto, Ont).

[40] El Haddad M, Moxham L, Broadbent M (2013). Graduate registered nurse practice readiness in the Australian context: an issue worthy of discussion. Collegian.

[41] Blaney DR (1986). An historical review of positions in baccalaureate education in nursing as basic preparation for professional nursing practice 1960–1984. J Nurs Educ.

[42] Rich KL, Nugent KE (2010). A United States perspective on the challenges in nursing education. Nurse Educ Today.

[43] WHO (2010). Strategic directions for strengthening nursing and midwifery services 2011–2015.

[44] International Council of Nurses (2006). The global nursing shortage: priority areas for intervention.

[45] Bell SA, Rominski S, Bam V, Donkor E, Lori J (2013). Analysis of nursing education in Ghana: priorities for scaling-up the nursing workforce. Nurs Health Sci.

[46] Pariyo GW, Kiwanuka SN, Rutebemberwa E, Okui O, Ssengooba F (2009). Effects of changes in the pre-licensure education of health workers on health-worker supply. Cochrane Database Syst Rev.

[47] WHO (2009). Global standards for the initial education of professional nurses and midwives.

[48] Zahran Z (2012). Nurse education in Jordan: history and development. Int Nurs Rev.

[49] Pardue SF (1987). Decision-making skills and critical thinking ability among associate degree, diploma, baccalaureate, and master's-prepared nurses. J Nurs Educ.

[50] Takase M, Nakayoshi Y, Yamamoto M, Teraoka S, Imai T (2014). Competence development as perceived by degree and non-degree graduates in Japan: a longitudinal study. Nurse Educ Today.

[51] Johnson JH (1988). Differences in the performances of baccalaureate, associate degree, and diploma nurses: a meta-analysis. Res Nurs Health.

[52] Bartlett HP, Simonite V, Westcott E, Taylor HR (2000). A comparison of the nursing competence of graduates and diplomats from UK nursing programmes. J Clin Nurs.

[53] Clinton M, Murrells T, Robinson S (2005). Assessing competency in nursing: a comparison of nurses prepared through degree and diploma programmes. J Clin Nurs.

[54] Fawcett J, Russell G (2001). A conceptual model of nursing and health policy. Policy Polit Nurs Pract.

[55] Gebbie KM, Wakefield M, Kerfoot K (2000). Nursing and health policy. J Nurs Scholarsh.

[56] 
Hewison A (2008). Policy analysis and policy implementation: can nursing manage without them?. J Nurs Manag.

[57] Leavitt J (2009). Leaders in health policy: a critical role for nursing. Nurs Outlook.

[58] Scott C, West E (2001). Nursing in the public sphere: health policy research in a changing world. J Adv Nurs.

[59] Fullbrook S (2008). Professional regulation in nursing. Part 1: an introduction. Br J Nurs.

[60] Kotze WJ (1995). The South African Nursing Council: 50 years of professional self-regulation. Curationis.

[61] Ellenbecker CH (2010). Preparing the nursing workforce of the future. Policy Polit Nurs Pract.

[62] Auerbach DI, Staiger DO, Muench U, Buerhaus PI (2013). The nursing workforce in an era of health care reform. New Engl J Med.

[63] Waters CD, Rochester SF, McMillan MA (2012). Drivers for renewal and reform of contemporary nursing curricula: a blueprint for change. Contemp Nurse.

[64] Spitzer A, Perrenoud B (2006). Reforms in nursing education across Western Europe: from agenda to practice. J Prof Nurs.

[65] McCarthy C, Riley P (2012). The African health profession regulatory collaborative for nurses and midwives. Hum Resour Health.

[66] McCarthy C, Voss J, Salmon M, Gross J, Kelley M, Riley P (2013). Nursing and midwifery regulatory reform in east, central, and southern Africa: a survey of key stakeholders. Hum Resour Health.

[67] Department of Health (2010). Negotiated service delivery Agreement.

# Sub article: Contestations and complexities of nurses’ participation in policy-making in South Africa

**DOI:** 10.3402/gha.v7.25327

**Published:** 2014-12-22

**Authors:** Prudence Ditlopo, Duane Blaauw, Loveday Penn-Kekana, Laetitia C. Rispel

**Affiliations:** Centre for Health Policy & Medical Research Council Health Policy Research Group, School of Public Health, Faculty of Health Sciences, University of the Witwatersrand, Johannesburg, South Africa

**Keywords:** nurses, participation, policy-making, contestations, South Africa

## Abstract

**Background:**

There has been increased emphasis globally on nurses’ involvement in health policy and systems development. However, there has been limited scholarly attention on nurses’ participation in policy-making in South Africa.

**Objective:**

This paper analyses the dynamics, strengths, and weaknesses of nurses’ participation in four national health workforce policies: the 2008 Nursing Strategy, revision of the Scope of Practice for nurses, the new Framework for Nursing Qualifications, and the Occupation-Specific Dispensation (OSD) remuneration policy.

**Methods:**

Using a policy analysis framework, we conducted in-depth interviews with 28 key informants and 73 front-line nurses in four South African provinces. Thematic content analysis was done using the Atlas.ti software.

**Results:**

The study found that nurses’ participation in policy-making is both contested and complex. The contestation relates to the extent and nature of nurses’ participation in nursing policies. There was a disjuncture between nursing leadership and front-line nurses in their levels of awareness of the four policies. The latter group was generally unaware of these policies with the exception of the OSD remuneration policy as it affected them directly. There was also limited consensus on which nursing group legitimately represented nursing issues in the policy arena. Shifting power relationships influenced who participated, how the participation happened, and the degree to which nurses’ views and inputs were considered and incorporated.

**Conclusion:**

The South African health system presents major opportunities for nurses to influence and direct policies that affect them. This will require a combination of proactive leadership, health policy capacity and skills development among nurses, and strong support from the national nursing association.

Notwithstanding the extraordinary and influential roles of many nurse leaders in the development of patient care and population health policies (1–3), several studies have highlighted the need for increased nurses’ involvement and participation in policy processes (4–12). ‘Participation’ in this context refers to actions and procedures designed to inform, consult, and involve the ‘community of nurses’, including nurse leaders and front-line nurses, to allow them to make inputs into those decisions that affect them (13). The International Council of Nurses (ICN) has stressed the importance of nurses’ contribution to health services planning and their participation in policy development (14). Considerable progress has been made in advancing nurses’ presence, role, and influence in health policy development (1, 15), illustrated by an increasing number of nurses elected as political office bearers and/or appointed to national and international boards (1).

In high-income countries such as Australia, Canada, the United Kingdom, and the United States of America, professional nursing associations are important platforms for individual nurses and the nursing profession to exercise power and influence policy (16–18). A recent review found that the common policy issues addressed by nursing associations include broader population health issues as well as professional or practice issues such as the scope of nursing practice, prescribing rights, education requirements, and workplace issues such as nursing shortages (19). There are examples from low-and middle-income countries (LMICs) where nurses have worked either individually or collectively through professional organisations to advocate for enabling health policies (20). In Rwanda, the chief nurse and the national nursing association mobilised support for legislation to improve the quality of nursing education and professional standards (20). In Paraguay, the nursing association capitalised on the visit of the chief executive officer (CEO) of the ICN to highlight poor staffing levels in health facilities in the country and to propose solutions to address them (20). In Kenya, the Nursing Council was a critical stakeholder in the development of the national electronic database on the nursing workforce (21).

Despite these encouraging developments, Leavitt (10) has argued that nurses are largely absent in health policy reforms compared to other professional and health interest groups. Their sub-optimal participation and limited role in health policy decisions is more acute in LMICs (10, 22, 23), where recent studies have underscored the need for increased nurses’ involvement in broader political and health policy debates (6, 8, 12), specifically with regard to HIV and AIDS policies (4, 11, 22). These LMIC participation studies have used different research designs, methods, and participants, including: a descriptive, mixed method study of hospital-based professional nurses and nurse leaders in Thailand (9); a qualitative case study with front-line nurses, nurse managers, and decision-makers in Kenya (4); in-depth interviews with policy-makers and a small survey with registered nurses in Botswana (22); a quantitative, descriptive study with registered nurses in Nigeria (6); and a Delphi-survey with nurse leaders from three East African countries (12). These studies have found that nurses have high levels of knowledge about national health policy development (9) on HIV and AIDS policies (22), but their role and participation in these policies were limited (4, 6, 9, 11, 12, 22). In those instances where nurses were involved, participation was limited to the policy implementation phase, rather than the full policy cycle from development to monitoring and evaluation (9, 12).

Phaladze (22) found that policy-makers were of the opinion that nurses did not have the expertise to participate in policy decisions, supporting the finding from other studies that the lack of policy and political skills is a hindrance for nurses’ policy participation (4, 9). Other barriers to nurses’ participation included: limited skills in public relations affecting their ability to explain and promote nursing (9); competing priorities (4, 23); insufficient time (4, 24); lack of resources (11, 24); insufficient involvement in policy formulation committees (9); and sub-optimal communication (11).

In South Africa, the Ministry of Health has emphasised the critical role of nurses in the implementation and success of health sector reforms towards universal health coverage (25). However, with the exception of a study that focused on the role of nurses in AIDS policy development (11), we could not find studies that explore nurses’ participation in broader health workforce policies. In light of limited empirical evidence and the centrality of the health workforce, specifically nurses, to health sector reforms (26, 27), this paper analyses the dynamics, strengths, and weaknesses of nurses’ participation in four national policies: the 2008 Nursing Strategy, revision of the Scope of Practice for nurses, the new Framework for Nursing Qualifications, and the Occupation-Specific Dispensation (OSD) financial incentive policy.

## Methods

The study was approved by the University of the Witwatersrand Human Research Ethics Committee (Medical) and the Provincial Health Research Ethics Committees in the four participating provinces (Eastern Cape, Free State, Gauteng and Western Cape). Hospital managers also provided permission to access their facilities. All participants received a study information sheet and gave informed written consent.

### Study design

A multiple descriptive case study design, informed by the Walt and Gilson policy analysis framework (28), was used. The framework focuses on four related factors critical to understanding public policy-making: actors, policy content, contextual factors, and process (28).

### Policies of interest

The study focused on four national policies: the 2008 Nursing Strategy, the Scope of Practice for nurses, the Framework for Nursing Qualifications, and the OSD.

The 2008 Nursing Strategy aimed to address the national nursing crisis by proposing action in six key strategic areas: nursing practice, nursing education and training, nursing leadership, nursing regulation, social positioning of nursing, and resources for nursing (29). The revised Scope of Practice for nurses is a legal document that outlines the role, responsibilities, and functions of different categories of nurses in the health system, whereas the new Framework for Nursing Qualifications is concerned with aligning nursing qualifications with the National Qualifications Framework. The OSD is a financial incentive strategy intended to attract, motivate, and retain health professionals in the public sector (30).

The selection of these four policies was influenced by two factors: prioritisation by key nursing stakeholders at a consultative workshop held in 2008 (31); and the policies represented different stages of policy development. For instance, the revisions of the Scope of Practice and the Qualifications Framework were at the development stage, the Nursing Strategy was at the initial phases of implementation and the OSD was fully implemented. These policies also differed in focus, the main policy drivers and actors involved, the degree of contestation about content, and the nature of the participatory processes.

### Study sites and setting

The study was conducted between 2009 and 2011 in four provinces in South Africa: Gauteng, Eastern Cape, Free State, and the Western Cape. The selected provinces were already part of a broader multi-year programme of research on nursing, initially chosen to allow for geographical comparisons (urban–rural) and possible variations in the interpretation and implementation of the selected policies. In each province, we selected one hospital randomly from each of the clusters of academic, regional, district, and specialised hospitals. The final sample consisted of 16 hospitals, four in each province.

### Study participants and data collection

Using a snowballing sampling technique, 28 key informants were selected purposively on the basis of their knowledge, involvement, or influence with the policy-making processes of the policies of interest. The key informants comprised the following groups of stakeholders: national government (*n*=6), provincial government (*n*=8), private sector (*n*=1), nursing educators/academics (*n*=7), statutory body (*n*=1), nursing association (*n*=4), and international non-profit global health organisation (*n*=1). The selected key informants were interviewed using a pilot-tested, semi-structured interview guide focusing on: the extent and nature of nurses’ participation and involvement in policy-making (both in general and in relation to the specific policies); the roles, interests, and influence of different nursing actors on these policies; and recommendations for improving nurses’ participation and involvement.

Semi-structured interviews were also conducted with 73 front-line nurses in the 16 selected hospitals, consisting of operational managers (*n*=15), professional nurses (*n*=15), enrolled nurses (*n*=13), enrolled nursing assistants (*n*=16), and shop stewards (*n*=14). The questions focused on the respondents’ awareness and knowledge of the policies under investigation, perceptions about the extent and nature of front-line nurses’ participation and involvement in nursing policy-making, and recommendations for improving nurses’ participation and involvement.

### Data analysis

A thematic content analysis of transcripts was conducted (32) using the Atlas.ti software. Once transcripts were loaded into the software, a line-by-line coding of each transcript was done using both inductive and deductive approaches to identify recurring themes. Thereafter, axial coding was conducted to identify connections and linkages in codes based on the conceptual framework (28). These themes and codes were used to organise the results in this paper. Three researchers (PD, DB, LPK), who received formal training in Atlas.ti, independently read and coded at least 12 transcripts and discussed discrepancies until agreement on the codes was reached, thereby ensuring coding consistency. To ensure the trustworthiness of the data, continuous peer debriefing and checking of researchers’ interpretations against the raw data was done. Where necessary, codes were renamed and redefined.

## Results

Three broad themes emerged from the analysis: the extent of nurses’ participation in nursing policies, the nature of participation, and contestations and complexities of participation.

### The extent of nurses’ participation in nursing policy processes

There was recognition and appreciation amongst several key informants that democracy created numerous opportunities for nurses’ participation in policy development. This was in contrast to the imposition of policies under apartheid. This increased ‘policy space’ occurred within the context of South Africa's rights-based Constitution. One key informant (KI) noted that:

Since 1994, there has been a lot more discussion and a lot more participation [of nurses]. Pre-1994, we were just sort of told ‘here is a policy’. (KI 7, Gauteng Department of Health)

When asked about nurses’ participation in the development of the four policies of interest, all key informants pointed to the wide range of policy actors involved to a greater or lesser degree in the development of these policies. However, there were contradictory views on participation, particularly in the case of the 2008 Nursing Strategy. Key informants from the National and Provincial Departments of Health were of the opinion that the process was nurse-led and that nursing leaders and representatives from all sections of the nursing profession had been involved, as can be seen from the excerpt below:

[The] Nursing Strategy is driven by nurses themselves because it started nationally when nurse leaders were called in to start with the Strategy. (KI 11, Eastern Cape Provincial Department of Health)

This was in contrast to respondents from outside the National and Provincial Departments who were of the opinion that the process and development of the Nursing Strategy were led by staff in the National Department who were not nurses. One said:

Very few nurses were involved in the development of the Nursing Strategy. Nurses were not at the policy table. (KI 26, Nursing Academic)

There was also a strong sense amongst front-line nurses and several key informants that front-line nurses were excluded from participation in broader health workforce policies. Front-line nurses believed they were excluded from policy processes because of the failure of policy-makers to recognise the importance of their clinical knowledge and expertise in informing policies. Some of these nurses commented that:

I don't understand how they [policy-makers] make decisions about nurses without involving the nurses. (Shop steward 37, Regional Hospital, Eastern Cape Province)

There are things that are decided by National [the National Department of Health] but when you are in the ward you see things practically and when you compare it to the policies, these [policies] are unrealistic. (Professional nurse 24, Regional Hospital, Gauteng Province)

Although respondents acknowledged that the participation of front-line nurses is important, some informants were of the view that it is impractical to involve all the nurses considering that they are the largest group of health professionals.

I think that in policy development, it would be impossible to get all the 120 or 140,000 nurses into one room and say ‘do you agree with this policy that has been proposed’. (KI 39, National Department of Health)

The study found that the nature of the policy determined the level of awareness of front-line nurses. Figure 1 shows nurses’ levels of awareness of the four policies analysed in this study.

**Fig. 1:** Front-line nurses awareness of nursing policies. ENA: enrolled nursing assistant; EN: enrolled nurse; PN: professional nurse; Opn Mx: operational manager; Shop Stwd: shop steward.

One hundred percent of enrolled nurses, professional nurses, operational managers, and shop stewards, and 88% of enrolled nursing assistants, reported that they have heard about the OSD policy. This was in contrast to the other three policies, where the level of awareness on policies was much lower. Operational managers and shop stewards were more likely to know about policies than other categories of nurses. The level of awareness among operational managers ranged from a low of 29% for the new Qualifications Framework to 71% for the new Scope of Practice for nurses. In the case of shop stewards, the level of awareness ranged from a low of 33% for the new Qualifications Framework to 67% for the Nursing Strategy. Figure 1 also shows that the level of awareness on the three policies among the different categories of general nurses was very low. One of the reasons mentioned was the lack of feedback by managers who attended the meetings where these policies were discussed.

### The nature of nurses’ participation

The majority of study participants were of the opinion that there was insufficient involvement of nurses in policy development. One key informant, reflecting on the OSD policy processes, commented that even though there was consultation with nursing leaders, their views were not incorporated into the policy, thus leading to unintended consequences during policy implementation.

It was a very top down approach such that those of us who were academics from the universities, I remember we actually left that meeting feeling like ‘what's the point’? There has always been a doctor behind it and telling nurses in a very top down way of saying ‘this is what the DoH [Department of Health] has decided and you shall do that!’ (KI 17, Nursing Academic)

A number of key informants were also of the view that nurse leaders are reactive rather than proactive when it comes to nursing policy processes. With regard to the Scope of Practice and Qualifications Framework policies, one key informant commented that nurse leaders often participate in policy-making when decisions have already been concluded:

When I think of nurse leaders, I think we are reactive because policies are being made in different areas, be it health, be it education, and we always seem like we come at a point when it is done and dusted; that's where we always find ourselves most of the time. We are happy to respond to what people say about us rather than challenge them. (KI 8, Gauteng Department of Health)

Another key informant was of the view that even though some nurses hold senior positions in provincial government and have the potential to influence policy, these nurses are not assertive enough to ensure that changes that could affect the nursing profession are achieved:

The challenge in South Africa about nursing is that, while we have nurses that are holding key positions, those nurses sometimes don't even have ‘teeth’ to change whatever they want to change. (KI 3, Nursing Association)

The key informants’ interviews thus identified two main categories for describing the nature of nurses’ participation in the policy processes. Individual participation happened when participants felt that their invitation to participate and be involved was on an individual basis and not intended to represent any specific interest group in nursing. Collective or representational participation happened when participants viewed their invitation to participate and be involved as representing a specific nursing interest group, such as the national nursing association, educators, or academia. Hence, participants’ views on the level and nature of participation differed depending on the policy of interest. For example, with regard to the Scope of Practice, the Qualifications Framework, and the OSD, the key actors were of the opinion that they either represented a particular nursing structure or interest group. With regard to the development of the Nursing Strategy, some informants were consulted in an individual capacity:

I made comments as an individual …. I participated as a consultant (KI 32, Nursing Academic)

I was involved and worked in a province but I wasn't representing a province – just sitting as an individual. (KI 7, Gauteng Department of Health)

Hence these informants appeared to view individual participation in the Nursing Strategy as a relatively minor contribution as it did not represent the views of the majority of nurses. According to key informants, the general approach to involving nurses in some of these policies tended to be formal and included activities such as serving on committees or boards, providing inputs into draft policy documents, chairing committees and/or trade union representation in collective bargaining processes. Because of the small pool of nurse leaders to draw from, the tendency of policy-makers to focus on multiple nursing policy issues in a short space of time required those involved to shift their attention constantly from one policy to another. They indicated that the OSD was introduced in 2007 but was implemented in 2008, whereas the Nursing Strategy was introduced in 2008. Around the same time period, debates around the Scope of Practice and the Qualifications Framework were also happening. One informant commented:

And I was involved in the Nursing Strategy after that, I was swallowed up in the OSD stories. Most of my time went into the OSD. (KI 8, Gauteng Department of Health)

With regard to the non-participation of front-line nurses, some informants were of the view that in a few instances where platforms were created to involve these nurses – such as inviting them to workshops – they seldom participated because they were intimidated by the presence of their managers. Overall, some of the reasons provided by key informants for the perceived limited participation of the broader nursing community in nursing policy processes include: the dominance of physicians and ‘others’ such as hospital CEOs and human resource managers, the under-representation of nurses in leadership positions, the predominantly female nature of the profession, and lack of training in policy and networking skills.

The other issue that I really see as a problem is that nurses as a whole are not trained in policy, they don't understand what policy is about and they don't understand the policy cycle. If you don't understand the policy cycle, then you will not know when there is a window of opportunity to influence policy. (KI 26, Nursing Academic)

We need to learn to be politically clever. Nurses don't have the skills of networking like businessmen do at the golf course. (KI 3, Nursing Association)

In general, a number of key informants commented that nurses themselves are to blame for their limited involvement and participation in policy-making processes because they tend to adopt a passive role in the policy-making process. This is mainly indicated through statements such as ‘nurses are not given an opportunity’ to participate in nursing policy processes or that ‘policies are developed without us’. Interestingly, some informants were optimistic that if nurses changed their way of thinking, they could turn the situation around and achieve maximum participation in health policy processes. Some informants commented that:

Unless the profession claims back what is ours, people shouldn't have this thing of, ‘you know we have not been consulted when the policy was being made?’ If you really stand your ground as the leader in your profession, and taking leadership in that regard, you will be involved in policy development. (KI 33, International Non-profit Health Organisation)

On the one hand, we are saying we are the backbone of the health care service, but we don't behave like we are the backbone. I think every nurse leader should make it her business to get involved in policy development. She should be staying close to debates and know exactly what is going on. (KI 25, Nursing Manager)

### Contestations and complexities of consultation and participation

The majority of key informants complained about the lack of cohesion or lack of collective action amongst different nursing stakeholders, which include the national nursing association, professional interest groups, university nursing academics, nursing college educators, nursing managers, the nursing council, and private sector nurses. These internal divisions discouraged collaboration among different nursing groups and made it difficult for nurses to have a unified voice, thus having a negative impact on how nurses were viewed by external actors, especially by key policy-makers. One informant explained:

We wanted to see the Minister of Health, but the Minister indicated that ‘Until the nurses have got one voice, I don't want to see them because sometimes there will be DENOSA [Democratic Nursing Organisation of South Africa] whose approach is more trade union kind of, there will be a group from the college, a group from the clinical side, a group from all those private sector people and you are all talking different things. Can you nurses just make up your mind and come as one voice to see me?’ (KI 32, Nursing Academic)

There were also apparent tensions expressed with regard to the position of nursing within the organisational structure of the National and Provincial Departments of Health. Depending on the province, the Nursing Division could report to Corporate Services, Hospital Services, or Human Resources for Health Directorates. A consistent view was that this hierarchy and lack of seniority of the designated nurses made it even more difficult to influence policy:

Most of the provinces, Nursing Directorates are reporting to the Hospital Service Managers who have a different mandate altogether because their interests is about hospitals, it's not about nurses only. (KI 3, Nursing Union)

In addition, some key informants also discussed the contestations around representation and the perceived legitimacy of some nursing groups. This was influenced by status and power relations within the profession. For example, there was a strong perception amongst some key informants that nurse academics or private sector nurses, had more prestige than those from nursing colleges or public health services. Therefore, there was a sense amongst these informants that prioritisation of nursing issues depended on how influential a certain group was and the agenda being pursued by a particular group. This tension was most prominent in relation to the Nursing Qualifications Framework.

Who is the Nursing Leadership because when I go there, they don't see me as representing a nursing voice? They would say ‘No, you are an academic’. (KI 32, Nursing Academic)

Several key informants expressed concerns about officials in the Human Resource Directorate of the National Department of Health who were leading the development of these four national policies, but who had limited or no understanding of the needs and expectations of nurses and the complexity of the nursing profession.

We keep on allowing other people to make decisions for us, people who don't understand how we work, people who don't understand how we are trained or how we think sometimes. (KI 27, Nurse Manager)

## Discussion

This study found that South Africa's democracy created opportunities and increased nurses’ participation in health policy development. Different nursing stakeholders – the national nursing association, professional interest groups, university nursing academics, nursing educators, nursing managers, the nursing council, and private sector nurses – were involved to a greater or lesser degree in the development of the four national policies of interest.

However, the study found that nurses’ participation in policy-making is both complex and contested. There was a disjuncture between nursing leadership and front-line nurses in their levels of awareness of the four policies analysed. Front-line nurses were generally unaware of the 2008 Nursing Strategy, the revised Scope of Practice for nurses, and the new Framework for Nursing Qualifications. The exception was their awareness of the OSD remuneration policy as it affected them directly. However, even within this group of front-line nurses, there were different levels of awareness with operational managers and union shop stewards more likely to know about the four policies of interest, compared to other categories of nurses. A minority of front-line nurses were aware of the new Framework for Nursing Qualifications which will change nursing education radically when implemented. These findings were in contrast to studies in Thailand (9) and Botswana (22) where nurses had high levels of knowledge about the health policies under investigation. The reason could be because these studies focused on nursing leaders, rather than on front-line nurses.

The majority of key informants and front-line nurses were of the opinion that nurses’ participation in nursing policy development was sub-optimal, thus supporting the findings of other studies of limited nurses involvement in health policy development (4, 6, 9, 11, 12, 22). Our study revealed various contestations regarding the extent and nature of nurses’ participation in nursing policies. Although there was consensus of the importance of front-line nurses, and expressed discomfort about their exclusion from policy participation, study respondents also acknowledged the practical difficulties of involving thousands of front-line nurses in broader health policy development, and overcoming the barriers to their active participation in forums which include their managers. This lack of active participation of front-line nurses could be explained by their position in the health hierarchy, where junior nurses are expected to follow orders, rather than question their seniors within nursing (33, 34).

Shifting power relationships influenced who participated (individual or stakeholder group), with contestations regarding the legitimacy of the different nursing stakeholder groups. This is not surprising as Buse et al. (35) have pointed out that organisations or groups may not all speak with one voice because they are made up of many different people whose values and beliefs may differ. These authors also argued that the decision-making process in the policy arena depends on the policy issue, its significance, the political system within which the policy is being made, the power of the various actors, and reconciliation of the different views of the interest groups (35). Therefore, contestation during policy-making should rather be understood as reflecting the reality that the nursing profession is not uniform and that policy-making is a struggle between groups with competing interests.

The degree to which nurses’ views and inputs were considered and incorporated was also contested, with the OSD policy given as an example of the unintended consequences that occurred during policy implementation, because these perspectives or insights from nurses were ignored. These consequences included demoralisation of front-line nurses; and adverse relationships between managers and nurses, and among different categories of nurses (36, p. 141).

The sub-optimal involvement of nurses in health policy development was exacerbated by both internal and external barriers. Internal barriers to nurses’ participation or involvement in broader health policies included the perceived reactive (as opposed to a more proactive) approach of nursing leadership; their relative lack of assertiveness and notions of victim mentality, even when they held senior provincial government positions; the small number of nurses with policy and/or advocacy skills; and the lack of cohesion or lack of collective action amongst different nursing stakeholders. A major external barrier was the position of nursing within the health hierarchy and organisational structures in South Africa. For example, at the time when this study was conducted, there was no chief nursing officer (CNO) or nursing directorate in the National Department of Health, the human resource division was headed by a medical doctor, and there were no nurses dealing with any of the four policies that primarily affected nurses. Buse et al. have noted that doctors were often more influential in public health policy either as civil servants or as health ministers (35), while Shariff and Potgieter (12) also found that nurses were mostly invisible and that the health policy agenda in Kenya, Uganda, and Tanzania was dictated by other health professionals, notably doctors. Other studies have also found that the relative position and power of midwives (37) lack of supportive organisational structures (38), inadequate political and policy development skills (9), competing priorities (4, 23), insufficient time (4, 24), lack of resources (11, 24), insufficient involvement in policy formulation committees (9), and sub-optimal communication (11) combined to produce a complex set of factors that mitigate against maximum participation of the nursing profession in health policy development. In South Africa, barriers to nurses’ participation in turn are shaped by dynamics of race, class, and gender (33, 34). Nonetheless, it would be erroneous to conclude that South African nurses are powerless or without agency. Rather, as Webber has pointed out, ‘nurses need to be recognised as active players whose involvements are among those structuring, reinforcing, or resisting their current realities’ (39, p. 9).

This paper makes an important contribution to both the national and international literature on health policy analysis. However, the findings may not be generalisable as the study was limited to four South African provinces and key informants were selected purposively. The data gathered also represent the perceptions of key informants and front-line nurses at a point in time. Nonetheless, the study provides rich insights into the dynamics, strengths, and weaknesses of nurses’ participation in four national health workforce policies.

Since the study was conducted, the first CNO in democratic South Africa has been appointed. The appointment of the CNO is a positive development, as the experience in other countries has shown the impact of such an appointment on cohesion and collective action by the nursing profession (1, 14). In light of the study findings, we recommend three strategies to increase nurses’ participation and involvement in health policy development. First, the CNO should provide leadership and serve as the ‘glue’ that brings together different nursing stakeholders to discuss and implement the recommendations contained in the Strategic Plan for Nursing Education, Training, and Practice (25). Second, the national nursing association should develop its own policy machinery to ensure that it has the capacity and skills to analyse, comment on, or lead the development of health policies. Third, the training of all nurses should include modules on health systems and policy development processes, leadership, and advocacy skills. Last, all work settings should explore simple, low-cost mechanisms to provide feedback on health sector or policy developments and to give nurses a voice to make inputs, in line with the recommendation at the 2013 Third Global Forum on Human Resources for Health (27).

## Conclusion

The importance of nurses to the success of health sector reforms in South Africa is unquestionable. There is evidence of the benefits to the health care system, patients, and the nursing profession when nurses are involved in health policy development (38, 40, 41). Nurses’ participation in the development of policies and strategies also enhances their job satisfaction and retention in the health sector (42). The South African health system presents major opportunities for nurses to influence and direct policies that affect them. This will require a combination of proactive leadership, health policy capacity and skills development among nurses, and strong support from the national nursing association.

## References

[1] Ferguson SL (2001). An activist looks at nursing's role in health policy development. J Obstet Gynecol Neonatal Nurs.

[2] Mechanic D, Rienhard SC (2002). Contributions of nurses to health policy. Nurs Health Pol Rev.

[3] Selander L, Crane P (2012). The voice of Florence Nightingale on advocacy. Online J Issues Nurs.

[4] Akunja E, Kaseje DCO, Obago I, Ochieng BM (2012). Involvement of hub nurses in HIV policy development: Case study of Nyanza Province, Kenya. Res Humanit Soc Sci.

[5] Archer SE (1983). A nurse administrator's political participation. West J Nurs Res.

[6] Asuquo EF, Etowa J, John M, Ndiok A, Sampson-Akpan P, Edet O (2013). Assessing nurses’ capacity for health research and policy engagement in Nigeria. J Appl Med Sci.

[7] Des Jardin KE (2001). Political involvement in nursing – politics, ethics, and strategic action. AORN J.

[8] Ehlers VJ (2000). Nursing and politics: a South African perspective. Int Nurs Rev.

[9] Kunaviktikul W, Nantsupawat R, Sngounsirithan U, Akkadechanunt T, Chitpakdee B, Wichaikhum O (2010). Knowledge and involvement of nurses regarding health policy development in Thailand. Nurs Health Sci.

[10] Leavitt JK (2009). Leaders in health policy: a critical role for nursing. Nurs Outlook.

[11] Richter MS, Mill J, Muller CE, Kahwa E, Etowa J, Dawkins P (2012). Nurses’ engagement in AIDS policy development. Int Nur Rev.

[12] Shariff N, Potgieter E (2012). Extent of East-African nurse leaders’ participation in health policy development. Nurs Res Pract.

[13] Smith LG Impact assessment and sustainable resource management.

[14] ICN Participation of nurses in health services decision-making and policy development. http://www.icn.ch/images/stories/documents/publications/position_statements/D04_Participation_Decision_Making_Policy_Development.pdf.

[15] Myers L (2007). Health policy analyst: an advanced practice registered subspecialty. Perioperat Nurs Clin.

[16] Haylock PJ, Mason DJ, Leavitt JK, Chaffee MW (2002). You and your professional nursing organization.

[17] Vandenhouten CL, Malakar CL, Kubsch S, Block DE, Gallagher-Lepak S (2011). Political participation of registered nurses. Pol Polit Nurs Pract.

[18] Matthews J (2012). Role of professional organizations in advocating for the nursing profession. Online J Issues Nurs.

[19] MacDonald J, Edwards N, Davies B, Marck P (2012). Priority setting and policy advocacy by nursing association: a scoping review and implications using a socio-ecological whole system lens. Health Policy.

[20] Benton D (2012). Advocating globally to shape policy and strengthen nursing's influence. Online J Issues Nurs.

[21] Riley PL, Vindigni SM, Arudo J, Waudo AN, Kamenju A, Ngoya J (2007). Developing a nursing database system in Kenya. Health Serve Res.

[22] Phaladze NA (2003). The role of nurses in the Human Immunodeficiency Virus/Acquired Immune Deficiency Syndrome policy process in Botswana. Int Nur Rev.

[23] Prescott PA, Dennis KE (1985). Power and powerlessness in hospital nursing departments. J Prof Nur.

[24] Cramer ME (2002). Factors influencing organized political participation in nursing. Policy Polit Nurs Pract.

[25] DOH (2013). Strategic Plan for Nursing Education, Training and Practice 2012/13–2016/17.

[26] Rispel LC, Barron P (2012). Valuing human resources: key to the success of a National Health Insurance System. Dev South Afr.

[27] Global Health Workforce Alliance, WHO (2013). A universal truth: no health without a workforce.

[28] Walt G, Gilson L (1994). Reforming the health sector in developing countries: the central role of policy analysis. Health Policy Plan.

[29] DOH (2008). Nursing Strategy for South Africa.

[30] Department of Public Service and Administration (2007). Implementation of the occupational specific dispensations (OSDs) for the occupations professional nurse, staff nurse and nursing assistants in the public service-File 18/2/P.

[31] Rispel L (2008). Research on the State of Nursing (RESON): exploring nursing policies, practice and management in South Africa-Workshop Report.

[32] Green J, Thorogood N (2004). Qualitative methods for health research.

[33] Marks S (1994). Divided sisterhood: race, class and gender in the South African nursing profession.

[34] Rispel L, Schneider H (1991). Professionalization of South African nursing: who benefits?. Int J Health Serv.

[35] Buse K, Mays N, Walt G (2012). Making health policy.

[36] Motsosi K, Rispel LC (2012). Nurses' perceptions of the implementation of occupational specific dispensation at two district hospitals in Gauteng. Afr J Nurs Midwifery.

[37] Matthews A, Scott PA (2008). Perspectives on midwifery power: an exploration of the findings of the inquiry into peripartum hysterectomy at our Lady of Lourdes Hospital, Drogheda, Ireland. Nurs Inquiry.

[38] Shariff N (2014). Factors that act as facilitators and barriers to nurse leaders’ participation in health policy development. BMC Nurs.

[39] Webber J (1999). Where are the nurses? An ethnographic study of nurses – seeking an explanation into their failure to redress chronic disparities in the late 1990's.

[40] Spenceley SM, Reutter L, Allen MN (2006). The road less traveled: nursing advocacy at the policy level. Policy Polit Nurs Pract.

[41] Gebbie KM, Wakefield M, Kerfoot K (2000). Nursing and health policy. J Nurs Scholarsh.

[42] Leveck ML, Jones CB (1996). The nursing practice environment, staff retention, and quality of care. Res Nurs Health.

# Sub article: Does moonlighting influence South African nurses’ intention to leave their primary jobs?

**DOI:** 10.3402/gha.v7.25754

**Published:** 2014-12-22

**Authors:** Laetitia C. Rispel, Tobias Chirwa, Duane Blaauw

**Affiliations:** 1 Centre for Health Policy & Medical Research Council Health Policy Research Group, School of Public Health, Faculty of Health Sciences, University of the Witwatersrand, Johannesburg, South Africa; 2 Division of Epidemiology and Biostatistics, School of Public Health, Faculty of Health Sciences, University of the Witwatersrand, Johannesburg, South Africa

**Keywords:** intention to leave, turnover, moonlighting, nurses, South Africa

## Abstract

**Background:**

Staff retention and turnover have risen in prominence in the global discourse on the health workforce. Moonlighting, having a second job in addition to a primary job, has not featured in debates on turnover.

**Objective:**

This paper examines whether moonlighting is a determinant of South African nurses’ intention to leave their primary jobs.

**Methods:**

During 2010, a one-stage cluster random sample of 80 hospitals was selected in four South African provinces. On the survey day, all nurses working in critical care, theatre, emergency, maternity, and general medical and surgical wards completed a self-administered questionnaire, after giving informed consent. In addition to demographic information and information on moonlighting, the questionnaire obtained information on the participants’ intention to leave their primary jobs in the 12 months following the survey. A weighted analysis of the survey data was done using STATA^®^ 13.

**Results:**

Survey participants (*n*=3,784) were predominantly middle-aged with a mean age of 41.5 (SD±10.4) years. Almost one-third of survey participants (30.9%) indicated that they planned to leave their jobs within 12 months. Intention to leave was higher among the moonlighters (39.5%) compared to non-moonlighters (27.9%; *p*<0.001). In a multiple logistic regression, predictors of intention to leave were moonlighting in the preceding year, nursing category, sector of primary employment, period working at the primary job, and number of children. The odds of intention to leave was 1.40 (95% CI: 1.16–1.69) times higher for moonlighters than for non-moonlighters. The odds ratio of intention to leave was 0.53 (95% CI: 0.42–0.66) for nursing assistants compared to professional nurses and 2.09 (95% CI: 1.49–2.94) for nurses working for a commercial nursing agency compared to those working in the public sector.

**Conclusion:**

Moonlighting is a predictor of intention to leave. Both individual and organisational strategies are needed to manage moonlighting and to enhance retention among South African nurses.

In recent years, staff retention, or the extent to which health care providers remain in the health system, has risen in prominence in the global discourse on the health workforce, because of its potential to improve their availability and accessibility (1–3). The related concept of staff turnover, or the rate at which an employer loses and gains employees (4, p. 1181), has generated numerous theoretical models that provide perspectives from the fields of economics, psychology, and organisational development (5–11). Notwithstanding inconsistencies in definitions and measurement, and the lack of differentiation between voluntary (employee-initiated) and involuntary (employer-initiated) turnover (9, 12, 13), the focus of these models has mostly been on voluntary turnover (5–11).

In the health system, high turnover of skilled health professionals has both economic and non-economic consequences (2, 11, 13, 14). These include the direct and indirect costs of recruitment and staff replacement, staff shortages, increased workloads of and demands on existing staff, and the potential risks of not being able to provide safe and quality care to patients (11, 13, 14). Nurse turnover has generated an extensive body of literature over several decades (4, 11, 13–25). Much of this literature on nurse turnover focuses on high-income countries, although there is increasing attention on turnover and its predictors in low- and middle-income countries (19, 26–29). Given the numerical dominance of nurses in many countries, high turnover among nurses has the potential to affect health care provision to patients and communities (3, 11, 13, 30, 31) and the morale, performance, and productivity of the remaining nurses (13, 32).

The reasons for high nurse turnover remain complex, and may vary over time (4). Nonetheless, existing evidence suggests that the causes of high turnover include various combinations of individual, organisational, and economic factors (4, 11–13, 15). At the individual level, the literature abounds with studies that demonstrate an inverse relationship between job satisfaction in nursing and turnover (4, 5, 7, 13, 15–19, 24, 25, 30, 33, 34). An equally consistent finding is that turnover intention (or intention to leave) is the strongest antecedent of actual turnover and is also an intermediate variable between job satisfaction and turnover (7–9). However, scholars have pointed out that the notion of job satisfaction itself is multifaceted and mediated by context, the work environment, demographic characteristics, personality type, individual motives, and experiences (4, 9, 13, 17, 22, 23, 30, 35–40). At the organisational level, studies have found that the factors that influence nurse turnover include workload, work schedules, workplace stress, leadership and management styles, training and promotional opportunities and a disjuncture between nurse expectations and the reality of the workplace (7, 21, 27, 30, 36, 41–45). In terms of economic factors, evidence suggests that remuneration and financial rewards influence staff turnover (4, 46), but financial incentives on their own are insufficient as a retention strategy (46).

Notwithstanding the voluminous literature, there are knowledge gaps in the studies on nurse turnover. These gaps include limited empirical information on the cost of nurse turnover to a health facility or the system as a whole and the impact of turnover on patient outcomes (13). Organisational development scholars have suggested that other forms of withdrawal such as absenteeism, passive job behaviour, or moonlighting (holding a second job in addition to a primary full-time job) could precede actual turnover (8, 9, 47, 48) and deserve more attention. We could not find any empirical studies that examine the relationship between moonlighting and nurse turnover intention or actual turnover.

In South Africa, the shortages and high turnover of nurses (49) impede the implementation of major health system reforms. Moonlighting is permitted in the South African public sector under specified conditions, which includes obtaining formal permission, though not all nurses do so (50). Nonetheless, moonlighting is widespread in South Africa. A 2010 survey found that 28.0% of nurses had done moonlighting in the year preceding the survey (51). Using data from the same survey, this paper examines factors associated with intention to leave and evaluates whether moonlighting is a predictor of nurses’ intention to leave their primary jobs. The findings of the study are part of a larger research project to examine casualisation in the nursing profession.

## Methods

During 2010, a one-stage cluster random sample of 80 hospitals was selected from the four South African provinces of the Eastern Cape (predominantly rural, but with a few large cities), Free State (mixed urban and rural), Gauteng (urban), and the Western Cape (predominantly urban). The Human Research Ethics Committee (Medical) of the University of the Witwatersrand in Johannesburg provided ethics approval for the study. The relevant public and private health care authorities also provided the necessary study approvals. All participants provided written, informed consent.

In each of the four provinces, the sampling frame consisted of all public and private hospitals, stratified by type of hospital for public hospitals; and by ownership and hospital bed numbers for private hospitals. A random sample of public and private sector hospitals was then selected from each stratum proportional to the total number of hospitals in that stratum.

On the 24-hour survey day, all nurses working in critical care, theatre, emergency, maternity, and general medical and surgical wards completed a self-administered questionnaire after giving informed consent. In addition to demographic characteristics and information on moonlighting, the questionnaire obtained data on the participants’ intention to leave their primary jobs in the 12 months following the survey. Further details of the survey methodology are provided in the previous article (51).

In the study moonlighting was defined as additional paid work – whether of a nursing or non-nursing nature – done by nurses in a private health facility, another government health facility, an insurance company, private health laboratory, or in the same health care facility while holding a primary, paid nursing job, but excluding overtime (51). Intention to leave was measured through one question: ‘In the next 12 months, do you plan to leave your current primary job?’

Data were weighted to reflect the population distribution of nurses between the public and private health sectors, and the four study provinces, and analysed using STATA^®^ 13. We also adjusted for the clustering and stratification introduced by the sampling design. Frequency tabulations were done to describe the socio-demographic characteristics of the respondents. Cross-tabulations were done to investigate associations of each of the factors, including moonlighting, with the intention to leave employment in the 12 months following the survey, our main outcome of interest. Bivariate logistic regression models were fitted and only factors found to be statistically significantly associated with intention to leave at a conservative 20% level were considered in the final model-building process using a multiple logistic regression model. All other statistical tests were carried out at 5% significance level.

## Results

### Participant characteristics

The unweighted demographic and background characteristics of the 3,784 nurses recruited in the four study provinces are shown in Table 1. The majority of survey participants were female (92.7%), and employed in provincial government (52.8%). The participants were predominantly middle-aged, with a mean age of 41.5 (SD±10.4) years. A few respondents omitted to complete some of the questions which accounts for the minor variations in denominators.

**Table 1:** Demographic and employment characteristics of survey participants

Characteristic	Total (*n*=3,784)
Mean age (standard deviation)	41.5 (10.4)
Age group (years)	
< 25	150 (4.1%)
25–34	888 (24.1%)
35–44	1,112 (30.2%)
45–54	1,115 (30.3%)
55+	414 (11.2%)
Sex	
Female	3,489 (92.7%)
Male	276 (7.3%)
Marital status	
Married	1,693 (45.0%)
Living together	130 (3.5%)
Single	1,328 (35.3%)
Divorced/separated	410 (10.9%)
Widowed	201 (5.3%)
Children	
Median number of children (range)	2 (1–14)
Median age of youngest child	12
Nursing category	
Professional nurse	1,910 (51.5%)
Enrolled nurse	818 (22.1%)
Auxiliary nurse	982 (26.5%)
Median years qualified as a nurse (mean)	15 (15.9)
Primary job (sector)	
Provincial government	1,955 (52.8%)
Private sector	1,400 (37.8%)
Nursing agency	346 (9.4%)
Median years at primary job (mean; range)	7 (10.3; 1–47)
Unit of work	
Paediatric critical care	183 (5.1%)
Adult critical care	421 (11.8%)
High care	33 (0.9%)
Theatre	668 (18.8%)
Emergency	392 (11.0%)
Maternity	574 (16.1%)
General wards	1,140 (32.0%)
Psychiatry	117 (3.3%)
Outpatient department	30 (0.8%)

### Factors influencing nurses’ intention to leave

In the study, 1,086 participants (30.9%) indicated that they planned to leave their primary jobs in the 12 months following the survey. Of these, 15.4% indicated that they planned to go overseas, 36.7% to move to another public sector job, and 13.5% to another private sector job. The remainder were made up of smaller proportions planning to work in nursing agencies or non-governmental organisations, or who planned to study, retire, or stay at home in the following year.

The study participants’ intention to leave their primary jobs varied by age, province, years at the primary job, nursing category, number of children, and moonlighting status (Table 2). 37.7% of nurses aged 25–34 years indicated their intention to leave, followed by 32.6% of nurses aged 35–44 years. Intention to leave was higher among nurses with no children (37.8%), compared to those with one child (30.4%), two children (30.9%), or four or more children (24.4%). In Gauteng Province, 37.8% of nurses indicated their intention to leave, compared to the Free State (28.1%), Western Cape (25.9%), or Eastern Cape (25.5%). Intention to leave was also higher among nurses working for a commercial nursing agency (44.1%) or the private health sector (36.9%), compared to the provincial government (28.2%). Those nurses with less than 1 year (22.3%) or 15–19 years of service (29.4%) were less likely to intend leaving their primary job (*p*<0.001) compared to those with between 5 and 9 years of service (39.2%).

**Table 2:** Bivariate analysis of factors influencing nurses’ intention to leave their jobs within 12 months

Variable	*n*	Intention to leave (%)	*P*
Total	3,513	1,086 (30.9%)	
Moonlighting in the past 12 months			<0.001
No	2,477	690 (27.9%)	
Yes	965	381 (39.5%)	
Province			<0.001
Gauteng	1,461	552 (37.8%)	
Eastern Cape	935	239 (25.5%)	
Western Cape	795	206 (25.9%)	
Free State	322	91 (28.1%)	
Age group (in years)			<0.001
< 25	126	34 (26.8%)	
25–34	864	326 (37.7%)	
35–44	981	320 (32.6%)	
45–54	1,026	265 (25.8%)	
55 +	410	106 (25.8%)	
Sex			0.181
Male	272	96 (35.3%)	
Female	3,234	988 (30.6%)	
Marital status			0.447
Married/living together	1,592	495 (31.1%)	
Single	1,406	447 (31.8%)	
Divorced/widowed	503	142 (28.2%)	
Number of children			0.005
None	593	224 (37.8%)	
One	794	241 (30.4%)	
Two	1,084	335 (30.9%)	
Three	669	194 (29.0%)	
Four or more	363	88 (24.4%)	
Sector			<0.001
Public	2,561	722 (28.2%)	
Private	671	248 (36.9%)	
Agency	216	95 (44.1%)	
Nursing category			<0.001
Professional nurse	1,682	609 (36.2%)	
Enrolled nurse	699	227 (32.5%)	
Nursing assistant	1,132	251 (22.1%)	
Years working at primary job			<0.001
Less than 1	379	84 (22.3%)	
1–4	911	321 (35.2%)	
5–9	652	255 (39.2%)	
10–14	323	100 (31.1%)	
15–19	298	88 (29.4%)	
20 or more	873	219 (25.1%)	

Table 2 also shows that intention to leave varied by nursing category (*p*<0.001). It was highest among professional or registered nurses with 4 years of training (36.2%), than among enrolled nurses with 2 years of training (32.5%) or among nursing assistants with 1 year of training (22.1%). Lastly, the proportion of participants with intention to leave in the 12 months following the survey was higher among the moonlighters compared to non-moonlighters (39.5% vs. 27.9%; *p*<0.001). Among those planning to go overseas, 18% (82) of moonlighters, compared to 13.8% (89) of non-moonlighters planned to go overseas, but this difference was not statistically significant (*p*=0.06).

### Predictors of intention to leave

In the multiple logistic regression analysis, predictors of intention to leave were: moonlighting in the preceding year, nursing category, sector of primary employment, period working at the primary job, and number of children (Table 3).

**Table 3:** Final multiple logistic regression model results for factors associated with nurses’ intention to leave their primary jobs within 12 months

Variable	Odds ratio	[95% CI]	*P*
Moonlighting in the previous 12 months			
No	–		–
Yes	1.40	[1.16–1.69]	<0.001
Province			
Gauteng	–	–	–
Eastern Cape	0.70	[0.54–0.91]	0.008
Western Cape	0.60	[0.49–0.75]	<0.001
Free State	0.71	[0.56–0.89]	0.004
Sector			
Provincial government	–	–	–
Private sector	1.11	[0.91–1.35]	0.293
Nursing agency	2.09	[1.49–2.94]	<0.001
Years working at primary job			
<1	–	–	–
1–4	2.21	[1.59–3.07]	<0.001
5–9	2.55	[1.80–3.61]	<0.001
10–14	1.69	[1.13–2.53]	0.011
15–19	1.70	[1.11–2.61]	0.016
20 or more	1.61	[1.12–2.31]	0.010
Nursing category			
Professional nurse	–	–	–
Enrolled nurse	0.79	[0.62–1.01]	0.058
Nursing assistant	0.53	[0.42–0.66]	<0.001
Number of children			
None	–	–	–
One	0.71	[0.54–0.95]	0.019
Two	0.71	[0.55–0.92]	0.010
Three	0.70	[0.52–0.95]	0.022
Four or more	0.59	[0.40–0.87]	0.008
Constant	0.44	[0.31–0.64]	<0.001

The weighted crude (unadjusted) odds for intention to leave the primary job in the 12 months following the survey were 1.69 (95% CI: 1.41–2.02) times higher among the moonlighters compared to the non-moonlighters. This was still significant (OR=1.40, 95% CI: 1.16–1.69) after adjusting for other factors such as nursing category, sector of primary job and years working in the primary job, sector and province of primary employment.

The adjusted analysis shows that individuals working for a commercial nursing agency (OR=2.09, 95% CI: 1.49–2.94) were more likely to express intention to leave, compared to those working in the provincial government. Enrolled nurses (OR=0.79, 95% CI: 0.62–1.01) or nursing assistants (OR=0.53, 95% CI: 0.42–0.66) were less likely to report intention to leave compared to professional nurses. The odds of individuals who have worked for 1–4 years to report intention to leave their primary job in the 12 months following the survey were 2.21 (92% CI: 1.59–3.07) times higher compared to those who have worked for less than 1 year. The intentions peak among those with 5–9 years’ experience (OR=2.55, 95% CI: 1.80–3.61). In later years of working experience, although the odds of participants reporting intention (ORs: 1.61–1.70) to leave were higher, the results show a fall in such intentions in relation to the reference group. The adjusted results also show a decline in nurses’ intentions to leave their primary jobs with increasing number of children. The odds ratios range from 0.71 (95% CI: 0.54–0.95) among those with one child to 0.59 (95% CI: 0.40–0.87) among those with four or more children compared to those with no children.

## Discussion

We found that almost one-third (30.9%) of respondents indicated their intention to leave their primary employment in the 12 months following the survey. This figure was lower than the turnover intent of nurses found in other studies done in South Africa in recent years (19, 26). A 2005 study to examine the relationship between job satisfaction, turnover intent, and demographic variables among primary health care nurses in a rural South African area found that 51.1% of these nurses considered leaving within 2 years following the study (19). Another study that compared the job satisfaction and intention to leave among different categories of health workers in Tanzania, Malawi, and South Africa found that 41.4% of South African health workers indicated that they were actively seeking other jobs, compared to 26.5% in Malawi, and 18.1% in Tanzania (26). However, these studies are not directly comparable as they comprised of different study populations, with different approaches to the measurement of intention to leave.

There are wide variations in study findings on turnover intent. The finding of 30.9% turnover intent in our study is similar to that found in Belgian hospitals done as part of a multi-country study – 30% of Belgian registered nurses indicated that they planned to leave their jobs (52). The multi-country hospital study, done in Europe and the United States of America (USA), examined patient safety, satisfaction, quality of hospital care, and nurse outcomes and found that intention to leave ranged from a low of 14% in the USA to a high of 49% in Greece and Finland (52). A 2007 study in Senegal among public sector midwives found that 58.9% of midwives reported their intention to leave within a year (28). This is in contrast to a study in Chinese hospitals that found that only 5% of nurses indicated their intention to leave (29). These differences in turnover intent might be explained by different contexts, study populations, and measurement methods.

In the multiple logistic regression analysis, the predictors of intention to leave were: category of nurse, primary employment in a commercial nursing agency, working for between 1 and 10 years at the primary job, and moonlighting in the preceding year (Table 3).

Enrolled nurses or nursing assistants were less likely to report intention to leave compared to professional nurses (Table 3). This may reflect the higher qualifications and skills of professional (registered) nurses, with greater potential for international movement and transferability of experience and skills. We could not find many studies that examined the relationship between nursing category, and intention to leave, as the bulk of the literature focuses on registered (professional) nurses (4, 13, 15, 42). A United Kingdom study that focused primarily on appropriate retention strategies found that enrolled nurses had greater job satisfaction than registered nurses and lower intention to quit, which may have been the result of ‘lower expectations in terms of pay and promotion due to their constrained promotion prospects’ (30, p. 689).

Surprisingly, nurses working for a commercial nursing agency were more likely to indicate their intention to leave, compared to those working in the public health sector. This may be related to the timing of the study, as there was a major public sector financial incentive policy implemented 2 years prior to the survey, which assisted in attracting large numbers of nurses back to the public health sector (53, 54).

The study found that those nurses working for between 1 and 10 years at the primary job were more likely to indicate their intention to leave. A 2005 study to examine the relationship between job satisfaction, turnover intent, and demographic variables among primary health care nurses in a rural area of South Africa found that turnover intent was significantly and inversely correlated with the number of years of nursing (19). However, the literature suggests that the relationship between length of service (tenure) and turnover is complex, because of possible confounding factors such as context, work environment, experience, and age (4, 13, 17, 25, 29, 42, 43, 45).

This is one of the first studies to examine the relationship between moonlighting and nurses’ intention to leave. We found a significant association between moonlighting and nurses’ intention to leave their primary jobs (39.5% among the moonlighters compared to 28% among non-moonlighters). The adjusted odds for intention to leave the primary job in the 12 months following the survey were 1.40 times higher among moonlighting nurses compared to the non-moonlighters. Organisational development researchers have suggested that moonlighting provides workers with an alternative source of income, training, and benefits, thus influencing turnover (47). This theory was supported by the 2010 moonlighting survey in South Africa that found multiple and varied motivations for moonlighting, including financial reasons and non-financial reasons such as taking care of patients, the opportunity to learn new nursing skills, and collegial relationships (51). However, moonlighting could also change staff perceptions, decisions, and behaviours, which may impact on turnover at their primary jobs either positively (in the decision to stay because of alternative benefits provided by the secondary job) or negatively (in accelerating actual turnover) (47). Our study suggests that moonlighting may accelerate nurse turnover intent. Although other studies have demonstrated that nurse absenteeism predicts turnover (11, 48, 55), we could not find similar or comparable studies on moonlighting and intention to leave in other low- or middle-income country settings, or even in high-income countries. Hence, more research is needed to determine whether and how moonlighting contributes to high nurse turnover intent, and ultimately to turnover.

The limitations of the study include: the cross-sectional study design which can only capture nurses remaining in their jobs; the possible social desirability bias resulting in lower disclosures of dissatisfaction, moonlighting, or intention to leave; and the fact that nurses who had not formally obtained permission for moonlighting may have been reluctant to admit to it. These limitations are discussed in more detail in the previous article (51). Although intention to leave is a very strong predictor of actual turnover, the expressed intentions of these nurses may not result in actual turnover. For example, the midwives study in Senegal found that although 58.9% reported their intention to leave within a year, the annual turnover rate was found to be only 9% due to limited job alternatives (28). Notwithstanding these limitations, our study is one of the first to examine the relationship between moonlighting and intention to leave. The study also enhances our understanding of the under-explored concept of moonlighting among South African nurses, who may be using moonlighting as a way of finding out whether changing jobs is possible and facilitating their decision-making to leave.

Our study findings have implications for health workforce policies and management, and for quality of care. As indicated above, high nurse turnover has the potential to affect health care provision to patients (3, 11, 13, 30, 31) and the morale, performance, and productivity of the remaining nurses (13, 32).

South Africa's five-year plan on human resources for health emphasises the importance of staff retention, both as a strategic imperative and as an outcome (49). Similarly, the Strategic Plan for Nursing Education, Training, and Practice highlights the importance of nurse retention (56). Moonlighting and high nurse turnover have to be addressed in tandem, as these issues have significant implications for quality of care in health facilities. The importance of safe, quality care to patients in South Africa is emphasised by the national core standards (57).

Although both staff retention and mechanisms to address quality of care are highlighted in various policy documents (49, 56, 57), a key challenge in South Africa has been in translating laudable plans and strategies into action (58, 59). Much more concrete action is needed to create positive practice environments, as there is evidence that these improve nurse retention and quality of patient care (13, 60, 61). In practical terms, strategies to create positive practice environments include: nurse participation in organisational matters; nursing practice which is flexible, meaningful and effective; leadership and support by nurse managers; adequate staffing and resources; and collaborative doctor-nurse relationships (61, p. 88).

A first step in the management of moonlighting is to recognise that it is widespread (51) and motivated by both financial and non-financial reasons. Hence, there is need for dialogue and debate on moonlighting in the South African health system, and the ethical and accountability issues that arise from nurses engaging in a second job, while employed full-time. The non-financial reasons for moonlighting such as recognition and appreciation for exemplary nursing services link to the strategies needed to create positive practice environments (60, 61). Key strategies such as participatory workplace forums and enhanced teamwork and collegial relationships do not require significant additional money or resources.

In the medium term, a uniform national monitoring and evaluation system should be developed, which includes indicators such as nurses’ absenteeism rates and trends, and total number of hours worked by each nurse, through improved employer personnel or nursing council information systems.

## Conclusion

Both the public and private health sectors in South Africa have a statutory duty to provide the best possible health care to patients within available resources and to achieve a balance between the rights and duties of health care providers (62). The study has found an association between moonlighting and intention to leave, which would need to be confirmed by other studies. The study points to the need for improved management of moonlighting and implementation of strategies for nurse retention in the South African health system.

## References

[1] WHO (2010). Increasing access to health workers in remote and rural areas through improved retention.

[2] Global Health Workforce Alliance, WHO (2013). A universal truth: no health without a workforce.

[3] Campbell J, Buchan J, Cometto G, David B, Dussault G, Fogstad H (2013). Human resources for health and universal health coverage: fostering equity and effective coverage. Bull World Health Organ.

[4] Currie EJ, Carr Hill RA (2012). What are the reasons for high turnover in nursing? A discussion of presumed causal factors and remedies. Int J Nurs Stud.

[5] Lambert EG, Hogan NL, Barton SM (2001). The impact of job satisfaction on turnover intent: a test of a structural measurement model using a national sample of workers. Soc Sci J.

[6] Bluedorn A (1982). A unified model of turnover from organizations. Hum Relat.

[7] Price JL, Mueller CW (1981). A causal model of turnover for nurses. Acad Manag J.

[8] Mobley W (1977). Intermediate linkages in the relationship between job satisfaction and employee turnover. J Appl Psychol.

[9] Tett R, Meyer J (1993). Job satisfaction, organizational commitment, turnover intention, and turnover: path analyses based on meta-analytic findings. Pers Psychol.

[10] Porter LW, Steers RM, Mowday RT (1974). Organizational commitment, job satisfaction, and turnover among psychiatric technicians. J Appl Psychol.

[11] Daouk-Oyry L, Anouze A-L, Otaki F, Dumit NY, Osman I (2014). The JOINT model of nurse absenteeism and turnover: a systematic review. Int J Nurs Stud.

[12] Tai TWC, Bame SI, Robinson CD (1998). Review of nursing turnover research, 1977–1996. Soc Sci Med.

[13] Hayes LJ, O'Brien-Pallasa L, Duffield C, Shamian J, Buchan J, Hughes F (2006). Nurse turnover: a literature review. Int J Nurs Stud.

[14] Jones C, Gates M (2007). The costs and benefits of nurse turnover: a business case for nurse retention. Online J Issues Nurs.

[15] Gauci Borda R, Norman IJ (1997). Factors influencing turnover and absence of nurses: a research review. Int J Nurs Stud.

[16] Chen H-C, Chu C-I, Wang Y-L, Lin L-C (2008). Turnover factors revisited: a longitudinal study of Taiwan-based staff nurses. Int J Nurs Stud.

[17] Coomber B, Barriball KL (2007). Impact of job satisfaction components on intent to leave and turnover for hospital-based nurses: a review of the research literature. Int J Nurs Stud.

[18] Currivan DB (1999). The causal order of job satisfaction and organizational commitment in models of employee turnover. Hum Resour Manag Rev.

[19] Delobelle P, Rawlinson JL, Ntuli S, Malatsi I, Decock R, Depoorter A-M (2011). Job satisfaction and turnover intent of primary healthcare nurses in rural South Africa: a questionnaire survey. J Adv Nurs.

[20] Gormley DK (2011). Are we on the same page? Staff nurse and manager perceptions of work environment, quality of care and anticipated nurse turnover. J Nurs Manag.

[21] Raup GH (2008). The impact of ED nurse manager leadership style on staff nurse turnover and patient satisfaction in academic health center hospitals. J Emerg Nurs.

[22] De Milt DG, Fitzpatrick JJ, Sister Rita M (2011). Nurse practitioners’ job satisfaction and intent to leave current positions, the nursing profession, and the nurse practitioner role as a direct care provider. J Am Acad Nurse Pract.

[23] Heinen MM, van Achterberg T, Schwendimann R, Zander B, Matthews A, Kozka M (2013). Nurses’ intention to leave their profession: a cross sectional observational study in 10 European countries. Int J Nurs Stud.

[24] Larrabee JH, Janney MA, Ostrow CL, Withrow ML, Hobbs GR, Burant C (2003). Predicting registered nurse job satisfaction and intent to leave. J Nurs Adm.

[25] McCarthy G, Tyrrell MP, Lehane E (2007). Intention to ‘leave’ or ‘stay’ in nursing. J Nurs Manag.

[26] Blaauw D, Ditlopo P, Maseko F, Chirwa M, Mwisongo A, Bidwell P (2013). Comparing the job satisfaction and intention to leave of different categories of health workers in Tanzania, Malawi, and South Africa. Glob Health Action.

[27] Nassar ME, Abdou HA, Mohmoud NA (2011). Relationship between management styles and nurses’ retention at private hospitals. Alexandria J Med.

[28] Rouleau D, Fournier P, Philibert A, Mbengue B, Dumont A (2012). The effects of midwives’ job satisfaction on burnout, intention to quit and turnover: a longitudinal study in Senegal. Hum Resour Health.

[29] Zhang L-F, You L-M, Liu K, Zheng J, Fang J-B, Lu M-M (2014). The association of Chinese hospital work environment with nurse burnout, job satisfaction, and intention to leave. Nurs Outlook.

[30] Shields MA, Ward M (2001). Improving nurse retention in the National Health Service in England: the impact of job satisfaction on intentions to quit. J Health Econ.

[31] Cometto G, Witter S (2013). Tackling health workforce challenges to universal health coverage: setting targets and measuring progress. Bull World Health Organ.

[32] Cavanagh SJ, Coffin DA (1992). Staff turnover among hospital nurses. J Adv Nurs.

[33] Decker FH, Harris-Kojetin LD, Bercovitz A (2009). Intrinsic job satisfaction, overall satisfaction, and intention to leave the job among nursing assistants in nursing homes. Gerontologist.

[34] Ramoo V, Abdullah KL, Piaw CY (2013). The relationship between job satisfaction and intention to leave current employment among registered nurses in a teaching hospital. J Clin Nurs.

[35] Bowling NA (2007). Is the job satisfaction–job performance relationship spurious? A meta-analytic examination. J Voc Behavior.

[36] Zeytinoglu IU, Denton M, Davies S, Baumann A, Blythe J, Boos L (2006). Retaining nurses in their employing hospitals and in the profession: effects of job preference, unpaid overtime, importance of earnings and stress. Health Pol.

[37] Camerino D, Conway PM, Van der Heijden BI, Estryn-Behar M, Consonni D, Gould D (2006). Low-perceived work ability, ageing and intention to leave nursing: a comparison among 10 European countries. J Adv Nurs.

[38] Fitzpatrick JJ, Campo TM, Graham G, Lavandero R (2010). Certification, empowerment, and intent to leave current position and the profession among critical care nurses. Am J Crit Care.

[39] Flinkman M, Laine M, Leino-Kilpia H, Hasselhorn H-M, Salantera S (2008). Explaining young registered Finnish nurses’ intention to leave the profession: a questionnaire survey. Int J Nurs Stud.

[40] Munyewende P, Rispel LC, Chirwa T (2014). Positive practice environments influence job satisfaction of primary health care clinic nursing managers in two South African provinces. Hum Resour Health.

[41] Duffield C, Roche M, O'Brien-Pallas L, Catling-Paull C, King M (2009). Staff satisfaction and retention and the role of the nursing unit manager. Collegian.

[42] Rambur B, Palumbo MV, McIntosh B, Mongeon J (2003). Statewide analysis of RNs’ intention to leave their position. Nurs Outlook.

[43] Tzeng H-M (2002). The influence of nurses’ working motivation and job satisfaction on intention to quit: an empirical investigation in Taiwan. Int J Nurs Stud.

[44] Van Bogaert P, Clarke S, Roelant E, Meulemans H, Van de Heyning P (2010). Impacts of unit-level nurse practice environment and burnout on nurse-reported outcomes: a multi-level modelling approach. J Clin Nurs.

[45] Takase M, Yamashita N, Oba K (2008). Nurses’ leaving intentions: antecedents and mediating factors. J Adv Nurs.

[46] Willis-Shattuck M, Bidwell P, Thomas S, Wyness L, Blaauw D, Ditlopo P (2008). Motivation and retention of health workers in developing countries: a systematic review. BMC Health Serv Res.

[47] Betts SC (2006). The decision to moonlight or quit: incorporating multiple jobholding into a model of turnover. J Organ Cult Comm Conflict.

[48] Davey MM, Cummings G, Newburn-Cook CV, Lo EA (2009). Predictors of nurse absenteeism in hospitals: a systematic review. J Nurs Manag.

[49] DOH (2011). Human Resources for Health South Africa: HRH strategy for the health sector: 2012/13–2016/17.

[50] Public Service Commission (PSC) (2007). Remunerative work outside the public service: an investigation undertaken in the Gauteng Provincial Health Sector.

[51] Rispel LC, Blaauw D, Chirwa T, de Wet K (2014). Factors influencing agency nursing and moonlighting among nurses in South Africa. Glob Health Action.

[52] Aiken LH, Sermeus W, Van den Heede K, Sloane DM, Busse R, McKee M (2012). Patient safety, satisfaction, and quality of hospital care: cross sectional surveys of nurses and patients in 12 countries in Europe and the United States. BMJ.

[53] Department of Public Service and Administration (2007). Implementation of the occupational specific dispensations (OSDs) for the occupations professional nurse, staff nurse and nursing assistants in the public service-File 18/2/P.

[54] Motsosi K, Rispel LC (2012). Nurses perceptions of the implementation of occupational specific dispensation at two district hospitals in Gauteng. Afr J Nurs Midwifery.

[55] Cohen A, Golan R (2007). Predicting absenteeism and turnover intentions by past absenteeism and work attitudes: an empirical examination of female employees in long term nursing care facilities. Career Dev Int.

[56] DOH (2013). Strategic Plan for Nursing Education, Training and Practice 2012/13–2016/17.

[57] DOH (2010). National core standards for health establishments in South Africa.

[58] Rispel L, Moorman J, Daniel J, Naidoo P, Pillay D, Southall R (2013). Health policy reforms and policy implementation in South Africa: a paradox?. New South African review 3: the second phase-tragedy or farce?.

[59] Rispel LC, Barron P (2012). Valuing human resources: key to the success of a National Health Insurance System. Dev South Afr.

[60] ICN (2010). Delivering quality, serving communities: nurses leading chronic care.

[61] Twigg D, McCullough K (2014). Nurse retention: a review of strategies to create and enhance positive practice environments in clinical settings. Int J Nurs Stud.

[62] Republic of South Africa (2005). National Health Act No. 61 of 2003.

# Sub article: The health system consequences of agency nursing and moonlighting in South Africa

**DOI:** 10.3402/gha.v8.26683

**Published:** 2015-05-11

**Authors:** Laetitia C. Rispel, Duane Blaauw

**Affiliations:** Centre for Health Policy & Medical Research Council Health Policy Research Group, School of Public Health, Faculty of Health Sciences, University of the Witwatersrand, Johannesburg, South Africa

**Keywords:** agency nursing, moonlighting, nurses, health system, quality of care, South Africa

## Abstract

**Background:**

Worldwide, there is an increased reliance on casual staff in the health sector. Recent policy attention in South Africa has focused on the interrelated challenges of agency nursing and moonlighting in the health sector.

**Objective:**

This paper examines the potential health system consequences of agency nursing and moonlighting among South African nurses.

**Methods:**

During 2010, a cluster random sample of 80 hospitals was selected in four South African provinces. On the survey day, all nurses providing clinical care completed a self-administered questionnaire after giving informed consent. The questionnaire obtained information on socio-demographics, involvement in agency nursing and moonlighting, and self-reported indicators of potential health system consequences of agency nursing and moonlighting. A weighted analysis was done using STATA^®^ 13.

**Results:**

In the survey, 40.7% of nurses reported moonlighting or working for an agency in the preceding year. Of all participants, 51.5% reported feeling too tired to work, 11.5% paid less attention to nursing work on duty, and 10.9% took sick leave when not actually sick in the preceding year. Among the moonlighters, 11.9% had taken vacation leave to do agency work or moonlighting, and 9.8% reported conflicting schedules between their primary and secondary jobs. In the bivariate analysis, moonlighting nurses were significantly more likely than non-moonlighters to take sick leave when not sick (*p*=0.011) and to pay less attention to nursing work on duty (*p*=0.035). However, in a multiple logistic regression analysis, the differences between moonlighters and non-moonlighters did not remain statistically significant after adjusting for other socio-demographic variables.

**Conclusion:**

Although moonlighting did not emerge as a statistically significant predictor, the reported health system consequences are serious. A combination of strong nursing leadership, effective management, and consultation with and buy-in from front-line nurses is needed to counteract the potential negative health system consequences of agency nursing and moonlighting.

Achieving universal health coverage to enable everyone to access the health services they need irrespective of ability to pay (1) and ensuring an adequately skilled, productive, and well-motivated health workforce (2) cannot be realised without addressing the global nursing crisis (3–5). This crisis is characterised by widespread shortages, an ageing workforce, excessive workloads, high turnover, skills gaps, and sub-optimal performance (3). The argument for addressing the nursing crisis is supported by well-documented evidence that the number, competencies, and effectiveness of nurses are critical in determining the quality of care and patient outcomes (6–16) and in improving the performance of weak health systems (4).

One aspect that has received inadequate attention in the description of the nursing crisis, by both the Global Health Workforce Alliance and the World Health Organization (WHO), is the casualisation of the nursing workforce and its implications for the nursing profession and for health system performance (2–4). The term ‘casualisation’ refers to the employment of workers on short-term contracts, without the rights and benefits associated with the standard contract of employment, namely full-time, permanent, continuing jobs (17). Although there are different types of casual or contingent work arrangements, the most visible form of casual work is through temporary staffing agencies (18) and moonlighting, defined as having a second job in addition to primary full-time employment. This policy gap exists despite increasing scholarly focus on the individual and organisational consequences associated with the greater reliance on casual or contingent staff in the workplace (17–26).

Research on casual work arrangements has focused *inter alia* on the commitment, roles, job satisfaction, conflict, perceived organisational support, organisational citizenship behaviours, health and well-being of contingent workers, and their performance in the workplace (18, 27). A review of research on casual or ‘precarious’ employment found an association between such employment and a deterioration in occupational health and safety in terms of injury rate and hazard exposures (25). Research on the performance of casual employees has yielded contradictory results, with their performance influenced by job satisfaction and commitment, type and scope of task allocation, and access to training (18).

A review of moonlighting among doctors in the health sector suggested that there are many negative health system consequences of this form of casualisation (28), including increased access barriers for patients, de-legitimisation of public sector health service delivery, reduction of trust between user and provider, lower quality of the care in the public sector, and accelerated migration to the private sector (28). In the case of agency nurses, there is evidence that casual or temporary staffing contributes to poor quality of patient care (29–32). In the United States, studies have found statistically significant associations between the employment of agency nurses and health care deficiencies in nursing homes (29), hospital medication errors (31), and the risk of bloodstream infections among patients with central venous catheters in intensive care units (32). In the United Kingdom, one study found that temporary staffing could undermine the quality of patient care (33), although another 5-year study of general and specialist wards found no differences in quality scores between temporary and permanent nursing staff (34).

Although several authors have highlighted the importance of understanding casualisation in low- and middle-income countries, particularly in the health sector (17, 28, 35, 36), much of the existing literature is concerned with high-income countries (18, 19, 22, 37–39). In South Africa, recent health policy attention has focused on the twin challenges of agency nursing and moonlighting (40, 41). A 2010 cross-sectional study found that agency nursing and moonlighting – two manifestations of casualisation in nursing – were common (42). The occurrence of moonlighting among nurses in the 12 months preceding the survey was 28.0% and of agency nursing was 37.8% (42). In light of the importance of nurses to improving the performance of the South African health system, this paper examines the potential health system consequences of agency nursing and moonlighting among South African nurses, using data from the same survey. The findings of the study are part of a larger research project to examine casualisation in the nursing profession.

## Methods

During 2010, a one-stage cluster random sample of 80 hospitals was selected from the four South African provinces of the Eastern Cape (mixed urban-rural). Free State (mixed urban-rural), Gauteng (urban), and the Western Cape (predominantly urban). The Human Research Ethics Committee (Medical) of the University of the Witwatersrand in Johannesburg provided ethics approval for the study. The relevant public and private health care authorities also provided the necessary study approvals. All participants provided written, informed consent.

In each of the four provinces, the sampling frame consisted of all public and private hospitals, stratified by type of hospital for public hospitals and by ownership and hospital bed numbers for private hospitals. A random sample of public and private sector hospitals was then selected from each stratum proportional to the total number of hospitals in that stratum.

In the study, moonlighting was defined as additional paid work – whether of a nursing or non-nursing nature – done by nurses in a private health facility, another government health facility, an insurance company, private health laboratory, or in the same health care facility while holding a primary, paid nursing job, but excluding overtime (42). Agency nursing was defined as any accredited nurse providing temporary cover in a hospital and paid for by a commercial nursing agency (42).

On the 24-hour survey day, all nurses working in critical care, theatre, emergency, maternity, and general medical and surgical wards completed a self-administered questionnaire after giving informed consent. The questionnaire obtained information on demographic characteristics, agency nursing and moonlighting, and participants’ experiences of health system incidents in the 12 months preceding the survey. These incidents are proxy indicators of health system consequences of agency nursing and moonlighting, identified in the international literature and in the formative research conducted prior to the survey. All participants were asked to indicate whether they had felt too tired to work while on duty; paid less attention to nursing work while on duty; taken sick leave when not sick; stayed away from work without authority to do so; or been involved in a medico-legal incident, for example administration of the wrong medication or a patient death (type 1 indicators). In addition, those participants who indicated that they had done moonlighting or agency nursing in the 12 months preceding the survey were asked to indicate whether they had argued with doctors or other nurses, experienced conflicting schedules between their primary and secondary jobs, taken sick leave to do agency work or moonlighting, taken vacation leave to do agency work or moonlighting, stayed away from work without authority to do agency work or moonlighting, or treated patients differently (e.g. shouted at patients) in their primary compared to their secondary job (type 2 indicators). Further details of the survey methodology are provided in a previous article (42).

Data were weighted to reflect the population distribution of nurses between the public and private health sectors and the four study provinces; and analysed using STATA^®^ 13. We also adjusted for the clustering and stratification introduced by the sampling design. Frequency tabulations were done to describe the socio-demographic characteristics of the respondents. Cross-tabulations were done to investigate associations of each of the factors, including agency nursing and moonlighting, with the type 1 and type 2 proxy indicators of health system consequences in the 12 months preceding the survey, our main outcomes of interest. Bivariate logistic regression models were fitted and only factors found to be statistically significantly associated with the health system consequences at a conservative 20% level were considered further in the final model-building process using multiple logistic regression. All other statistical tests were considered significant at the 5% level.

We also used multiple correspondence analysis (MCA) to derive two indices of health system consequences (for type 1 and type 2 indicators). MCA is a data reduction method similar to principal component analysis but more appropriate for categorical data (43, 44). The MCA index was normalised to a mean of zero and a standard deviation of 1, hence positive scores indicate more adverse health system consequences. Bivariate differences in the consequence indices were tested by *t*-tests and ANOVA.

## Results

### Participant characteristics

The majority of survey participants were female (92.7%) and employed in provincial government (52.8%). The participants were predominantly middle-aged, with a mean age of 41.5 (SD: 10.4) years. The unweighted demographic and background characteristics of the 3,784 nurses recruited in the four study provinces are shown elsewhere in this special journal issue (45). Importantly, 40.7% (95% CI: 35.3–46.4) of nurses indicated that they had moonlighted or worked for an agency in the 12 months prior to the survey.

### Occurrence of health system consequences

In the study, 51.5% of all participants said that they felt too tired to work while on duty, 11.5% paid less attention to nursing work while on duty, 10.9% had taken sick leave when not actually sick, 5.6% had stayed away from work without authority, and 2.9% reported being involved in a medico-legal incident (Table 1). More than half (55.8%) of nurses were involved in any of these incidents used as proxy indicators of health system consequences. The mean of the MCA composite index combining these variables was 0.761, indicating that on average there were more undesirable consequences.

**Table 1:** Health system consequences of moonlighting and agency work

			Felt too tired to work while on duty		Paid less attention to nursing work while on duty		Took sick leave when not actually sick		Stayed away from work without authority		Involved in a medico-legal incident		Any of these		Composite Consequence Index 1 (MCA*)	
									
Variable		*n*	%	*p*	%	*p*	%	*p*	%	*p*	%	*p*	%	*p*	Mean	*p*
Total		3708	51.5		11.5		10.9		5.6		2.9		55.8		0.761	
Moonlighting or agency	No	2143	52.2	0.442	10.3	**0.035**	9.7	**0.011**	4.9	0.090	2.6	0.169	56.5	0.463	−0.018	0.138
nursing	Yes	1473	50.5		13.2		12.9		6.5		3.4		55.0		0.032	
Province	Gauteng	1638	55.6	**0.005**	13.6	**0.049**	13.2	**0.018**	6.9	**0.012**	3.1	0.299	59.5	**0.007**	0.197	**<0.001**
	Eastern Cape	945	54.6		10.6		10.4		6.0		2.4		59.4		0.001	
	Western Cape	800	44.2		9.2		9.3		3.3		2.7		48.1		−0.102	
	Free State	325	40.5		9.4		5.5		4.3		4.4		45.6		−0.135	
Sex	Male	289	53.4	0.621	14.4	0.135	14.8	0.090	6.7	0.416	3.5	0.579	58.6	0.447	0.230	<**0.001**
	Female	3412	51.3		11.2		10.6		5.5		2.9		55.5		−0.013	
Age group	<25 years	139	54.5	<**0.001**	14.3	**0.017**	14.8	<**0.001**	7.2	<**0.001**	4.0	0.290	60.4	<**0.001**	0.183	<**0.001**
	25–34 years	926	59.4		15.1		16.6		9.5		4.0		63.9		0.235	
	35–44 years	1024	52.4		11.0		11.0		5.2		2.9		56.6		0.026	
	45–54 years	1060	50.0		10.4		8.9		4.4		2.3		54.4		−0.094	
	55+ years	430	35.8		8.3		3.5		1.7		1.9		40.3		−0.315	
Marital status	Married/living together	1667	51.9	**0.011**	11.5	0.513	11.0	0.231	4.8	<**0.001**	3.2	0.509	56.5	**0.004**	−0.021	**0.007**
	Single	1495	53.3		10.9		11.6		7.4		2.4		57.7		0.071	
	Divorced/widowed	530	44.7		13.1		8.2		3.2		3.3		47.9		−0.070	
Any children?	No	619	56.6	0.084	15.6	**0.012**	13.2	0.099	4.9	0.457	4.1	0.129	62.3	**0.016**	0.120	**0.002**
	Yes	3089	50.5		10.7		10.5		5.8		2.7		54.5		−0.019	
Sector	Public	2692	54.3	<**0.001**	11.0	**0.012**	11.5	**0.011**	5.8	**0.002**	2.4	**0.041**	58.3	<**0.001**	0.078	<**0.001**
	Private	681	46.5		15.1		7.1		2.9		4.2		51.9		−0.075	
	Agency	259	35.3		8.7		12.7		9.1		3.6		39.7		−0.113	
Nursing category	Professional nurse	1759	57.4	<**0.001**	15.2	<**0.001**	12.3	0.134	5.5	0.526	3.9	**0.010**	61.5	<**0.001**	0.110	<**0.001**
	Enrolled nurse	740	52.1		9.1		10.6		6.6		2.2		56.3		−0.038	
	Nursing assistant	1209	42.3		7.4		9.1		5.2		2.0		47.0		−0.162	
Unit	General wards	1160	51.3	0.482	12.1	0.134	11.1	0.962	6.1	0.258	3.1	0.632	56.1	0.305	−0.007	0.355
	Maternity	599	52.5		8.9		11.2		4.2		2.8		55.7		−0.044	
	ICU	536	50.5		14.7		11.8		4.1		3.2		56.4		0.062	
	Theatre	570	55.2		11.6		10.1		7.5		1.9		59.7		0.039	
	Other	834	49.1		10.5		10.6		5.7		3.1		52.3		−0.012	
Years working at	Less than 1 year	404	47.2	0.257	10.7	0.146	8.4	**0.005**	4.8	0.389	2.3	**0.005**	50.6	0.310	−0.047	<**0.001**
primary job	1–4 years	982	53.2		10.3		11.6		6.9		4.0		57.4		0.054	
	5–9 years	675	55.3		14.5		15.8		6.3		2.1		59.2		0.097	
	10–14 years	339	54.9		13.1		13.4		4.5		4.6		58.5		0.037	
	15–19 years	312	53.0		13.5		8.4		3.8		4.6		57.9		−0.029	
	20 or more years	904	49.0		10.2		7.6		4.9		1.3		54.3		−0.106	

Type 1 indicators in all nurses; Statistically significant relationships in bold.

* Multiple correspondence analysis.

Table 1 compares the occurrence of health system consequences between nurses who had moonlighted or worked for an agency in the preceding year and those who had not. Moonlighting nurses were significantly more likely to take sick leave when not sick (12.5% vs. 9.7%; *p*=0.011) and to pay less attention to nursing work while on duty (13.2% vs. 10.3%, *p*=0.035), but there were no significant differences between the two groups for any of the other type 1 indicators. The MCA composite index was higher for moonlighting nurses but again the difference was not statistically significant. Table 1 also shows the bivariate analysis of other socio-demographic factors associated with these outcomes. Significant differences were noted for different individual outcomes and for the composite index between different provinces, age groups, sectors of work, and nursing categories.

Type 2 indicators were only collected among those participants that had done moonlighting or agency nursing in the year preceding the survey (Table 2). In this group, 19.6% reported that they argued with their colleagues, 9.8% reported conflicting schedules between their primary and secondary jobs, but only 2.3% reported that they treated patients differently in the primary compared to the secondary job. In addition, 11.9% reported that they had taken vacation leave and 2.8% had taken sick leave to do agency work or moonlighting, but only 1.6% indicated that they stayed away from work without authority to moonlight or work for an agency in the preceding year. One-third of moonlighters (33.7%) reported any of these type 2 indicators, and the average score of the MCA composite index derived from these variables was positive at 0.422. Again the bivariate analysis suggested differences for certain of these outcomes between provinces, age groups, sector of work, and nursing category (Table 2).

**Table 2:** Health system consequences of moonlighting and agency work

			Argued with doctors or other nurses		Conflicting schedules between primary and secondary jobs		Took vacation leave to do agency work or moonlighting		Took sick leave to do agency work or moonlighting		Stayed away without authority to do agency work or moonlighting		Treated patients differently in primary vs. secondary job		Any of these		Composite Consequence Index 2 (MCA)	
										
Variable		*n*	%	*p*	%	*p*	%	*p*	%	*p*	%	*p*	%	*p*	%	*p*	Mean	*p*
Total		1473	19.6		9.8		11.9		2.8		1.6		2.3		33.7		0.422	
Province	Gauteng	897	23.2	<**0.001**	12.1	<**0.001**	13.9	**0.011**	3.2	0.204	2.4	0.475	3.4	**0.002**	38.5	<**0.001**	0.218	<**0.001**
	Eastern Cape	122	24.1		6.4		11.2		2.8		0.0		0.2		34.3		−0.102	
	Western Cape	334	11.4		4.3		6.5		2.7		0.0		0.6		20.7		−0.205	
	Free State	120	12.1		11.9		13.9		0.6		1.8		1.7		34.7		−0.047	
Sex	Male	107	25.3	**0.042**	13.2	0.188	16.8	0.140	1.6	0.372	2.1	0.579	1.2	0.459	39.9	0.211	0.219	**0.015**
	Female	1362	19.1		9.6		11.6		2.9		1.6		2.4		33.2		−0.014	
Age group	<25 years	51	15.0	0.651	8.8	0.397	2.4	0.070	3.5	0.521	3.4	0.500	3.5	0.718	22.9	0.123	0.036	**0.035**
	25–34 years	402	21.6		10.7		11.2		3.8		2.0		3.3		36.4		0.039	
	35–44 years	463	20.1		11.9		15.4		2.7		1.3		2.2		37.2		0.075	
	45–54 years	377	18.1		7.2		11.3		2.7		0.9		1.5		29.6		−0.080	
	55+ years	114	16.2		9.9		8.6		0.0		0.0		3.5		28.7		−0.181	
Marital status	Married/living together	648	19.1	0.971	9.7	0.523	12.3	0.336	3.0	0.661	1.7	0.978	1.8	0.247	32.9	0.541	−0.011	0.832
	Single	613	19.8		9.1		10.8		2.9		1.6		2.3		33.2		0.002	
	Divorced/widowed	199	19.7		11.7		14.9		1.5		1.5		4.4		37.7		0.033	
Any children?	No	232	22.2	0.305	9.6	0.886	10.9	0.623	2.2	0.592	1.2	0.671	2.0	0.751	33.9	0.920	−0.023	0.657
	Yes	1241	19.0		9.9		12.1		2.9		1.7		2.4		33.6		0.007	
Sector	Public	823	20.1	0.134	9.9	0.916	12.7	**0.001**	3.1	0.191	1.2	**0.004**	3.4	0.151	34.8	**0.019**	0.116	**0.001**
	Private	413	21.1		9.3		15.1		1.7		0.7		0.9		35.8		−0.091	
	Agency	203	12.6		9.8		2.2		3.7		6.0		1.5		22.6		0.017	
Nursing category	Professional nurse	735	22.7	0.068	9.5	0.543	17.9	<**0.001**	2.5	0.114	1.2	0.547	2.7	0.284	38.7	**0.003**	0.049	0.109
	Enrolled nurse	315	15.2		11.5		8.3		4.5		1.9		1.0		27.9		−0.024	
	Nursing assistant	423	17.0		9.1		3.7		2.0		2.2		2.8		28.7		−0.081	
Unit	General wards	386	17.7	0.239	10.2	0.167	6.3	<**0.001**	3.3	0.605	1.8	0.290	1.6	0.296	29.3	**0.009**	−0.043	**0.014**
	Maternity	274	18.0		6.5		6.9		1.4		0.0		2.1		25.6		−0.139	
	ICU	372	17.9		11.1		20.9		3.7		2.1		3.3		39.3		0.129	
	Theatre	207	26.3		14.8		12.1		2.0		1.2		3.7		43.0		0.031	
	Other	234	21.3		6.7		12.9		3.1		2.8		1.1		33.5		0.001	
Years working at	Less than 1 year	180	12.6	0.186	10.3	0.428	8.6	**0.047**	5.0	0.154	2.0	0.360	2.6	0.240	27.6	0.157	−0.011	0.878
primary job	1–4 years	448	21.8		7.8		8.7		3.4		2.9		3.0		32.3		0.028	
	5–9 years	290	18.7		12.4		13.7		2.2		0.9		0.9		36.8		−0.023	
	10–14 years	170	21.9		12.4		17.7		4.6		1.2		1.7		39.1		0.070	
	15–19 years	125	24.7		9.1		18.1		0.0		0.0		1.1		41.5		−0.038	
	20 or more years	205	19.0		7.7		13.4		0.7		0.7		4.8		32.6		−0.021	

Type 2 indicators in moonlighting nurses only; Statistically significant relationships in bold.

### Predictors of negative health system consequences among all participants

Table 3 shows the results of the multiple regression analysis used to investigate the impact of moonlighting on the type 1 health system consequences, while adjusting for other socio-demographic factors. Among moonlighters, the odds of “staying away from work without authority” were 1.31 (95% CI: 0.86–1.98) times higher than for non-moonlighters, and the odds of “taking sick leave when not actually sick” were 1.36 (95% CI: 0.95–1.94) higher, but these differences were only statistically significant at the 10% level in the multiple regression. Moonlighters were 1.19 (95% CI: 0.88–1.62) times more likely to report “paying less attention to nursing work while on duty,” which was also not statistically significant. The odds of “being involved in a medico-legal incident” and “feeling too tired at work” were similar in the two groups. Overall, experiencing any of these incidents was equally likely in the two groups (OR: 0.99; 95% CI: 0.80–1.20).

**Table 3:** Multiple logistic regression of predictors of health system consequences

		Felt too tired to work while on duty	Paid less attention to nursing work while on duty	Took sick leave when not actually sick	Stayed away from work without authority	Involved in a medico-legal incident	Any of these
							
Variable		OR (95% CI)	OR (95% CI)	OR (95% CI)	OR (95% CI)	OR (95% CI)	OR (95% CI)
Moonlighting or	No	–	–	–	–	–	–
agency nursing in	Yes	0.97	1.19	1.36	1.31	1.01	0.99
the past 12 months		(0.79–1.19)	(0.88–1.62)	(0.95–1.94)	(0.86–1.98)	(0.63–1.62)	(0.81–1.20)
Province	Gauteng	–	–	–	–		–
	Eastern Cape	0.95	0.87	1.02	1.03		1.02
		(0.60–1.51)	(0.59–1.28)	(0.72–1.45)	(0.65–1.63)		(0.64–1.62)
	Western Cape	0.65**	0.67**	0.84	0.54***		0.64**
		(0.49–0.85)	(0.52–0.85)	(0.53–1.36)	(0.40–0.75)		(0.49–0.83)
	Free State	0.58***	0.71*	0.44***	0.74		0.60***
		(0.42–0.78)	(0.51–0.98)	(0.29–0.68)	(0.49–1.11)		(0.45–0.80)
Sex	Male		–	–			
	Female		0.78	0.76			
			(0.50–1.21)	(0.47–1.23)			
Age group	<25 years	–	–	–	–		–
	25–34 years	1.32	1.10	0.88	1.30		1.27
		(0.77–2.27)	(0.63–1.91)	(0.46–1.65)	(0.66–2.55)		(0.83–1.95)
	35–44 years	0.94	0.62	0.45*	0.69		0.89
		(0.55–1.61)	(0.34–1.13)	(0.24–0.87)	(0.32–1.48)		(0.57–1.39)
	45–54 years	0.83	0.60	0.37*	0.71		0.81
		(0.47–1.49)	(0.35–1.02)	(0.18–0.79)	(0.31–1.60)		(0.51–1.29)
	55+ years	0.47*	0.45*	0.15***	0.19*		0.46**
		(0.24–0.91)	(0.22–0.93)	(0.06–0.38)	(0.04–0.97)		(0.27–0.80)
Marital status	Married/living together	–			–		–
	Single	0.95			1.21		0.93
		(0.79–1.13)			(0.83–1.78)		(0.78–1.11)
	Divorced/widowed	0.99			0.84		0.94
		(0.77–1.26)			(0.48–1.48)		(0.74–1.20)
Any children?	No	–	–	–		–	–
	Yes	0.86	0.79	0.94		0.65	0.78
		(0.63–1.16)	(0.57–1.11)	(0.64–1.38)		(0.35–1.19)	(0.58–1.03)
Sector	Public	–	–	–	–	–	–
	Private	0.68**	1.30*	0.49**	0.50*	1.32	0.73*
		(0.53–0.89)	(1.02–1.66)	(0.32–0.75)	(0.30–0.86)	(0.79–2.21)	(0.57–0.94)
	Agency	0.50***	0.79	1.27	1.40	1.56	0.50***
		(0.38–0.66)	(0.48–1.31)	(0.80–1.99)	(0.82–2.40)	(0.80–3.04)	(0.37–0.67)
Nursing category	Professional nurse	–	–	–		–	–
	Enrolled nurse	0.77	0.57**	0.73		0.58	0.77
		(0.56–1.04)	(0.39–0.83)	(0.52–1.04)		(0.31–1.06)	(0.58–1.04)
	Nursing assistant	0.52***	0.44***	0.71		0.50*	0.54***
		(0.41–0.66)	(0.30–0.65)	(0.49–1.04)		(0.26–0.93)	(0.42–0.69)
Unit	General wards		–				
	Maternity		0.63*				
			(0.43–0.93)				
	ICU		0.91				
			(0.62–1.33)				
	Theatre		0.89				
			(0.63–1.25)				
	Other		0.82				
			(0.56–1.20)				
Years working at	Less than 1 year		–	–		–	
primary job	1–4 years		0.93	1.57*		1.82	
			(0.63–1.37)	(1.00–2.46)		(0.90–3.68)	
	5–9 years		1.45	2.88***		0.95	
			(0.97–2.16)	(1.80–4.63)		(0.38–2.38)	
	10–14 years		1.40	2.71**		1.89	
			(0.89–2.20)	(1.43–5.12)		(0.69–5.16)	
	15–19 years		1.56	1.79		2.14	
			(0.94–2.60)	(0.85–3.79)		(0.80–5.76)	
	20 or more years		1.45	2.00		0.58	
			(0.89–2.37)	(0.88–4.55)		(0.21–1.59)	
Constant		2.36**	0.38*	0.21***	0.07***	0.04***	3.09***
		(1.42–3.94)	(0.17–0.82)	(0.10–0.47)	(0.03–0.21)	(0.02–0.09)	(2.16–4.44)
Observations		3357	3277	3281	3354	3401	3375
Model *p* value		<0.001	<0.001	<0.001	<0.001	<0.001	<0.001

Type 1 indicators in all nurses.

*** *p*<0.001,

** *p*<0.01,

* *p*<0.05.

Instead, the multiple logistic regression analysis found that the differences for these variables were explained by province (geographical location), age, sector of employment, nursing category, and the number of years at the primary job (Table 3).

In terms of geographical location and relative to Gauteng, those participants from the Western Cape were significantly less likely to report that they stayed away from work without authority (OR: 0.54; 95% CI: 0.40–0.75); felt too tired to work while on duty (OR: 0.65; 95% CI: 0.49–0.85); or paid less attention to nursing work while on duty (OR: 0.67; 95% CI: 0.52–0.85). Participants from the Free State province were also less likely to report that they felt too tired to work while on duty, took sick leave when not actually sick, or paid less attention to nursing work while on duty (Table 3).

Interestingly, those nurses over 55 years old were much less likely to report that they stayed away without authority to do so (OR: 0.19; 95% CI: 0.04–0.97); felt too tired to work while on duty (OR: 0.47; 95% CI: 0.24–0.91); had taken sick leave when not actually sick (OR: 0.15; 95% CI: 0.06–0.38); or paid less attention to nursing work while on duty (OR: 0.45; 95% CI: 0.22–0.93).

Relative to the public sector participants, those nurses from the private sector were less likely to report that they stayed away from work without authority (OR: 0.50; 95% CI: 0.30–0.86), felt too tired to work while on duty (OR: 0.68; 95% CI: 0.53–0.89); or had taken sick leave when not actually sick (OR: 0.49; 95% CI: 0.32–0.75). However, they were more likely to report that they paid less attention to nursing while at work (OR: 1.30; 95% CI: 1.02–1.66). Those from a commercial nursing agency were also less likely to report that they felt too tired to work while on duty (OR: 0.50; 95% CI: 0.38–0.66).

The analysis found that nursing assistants were less likely than professional nurses to report any of the incidents measured in the study. However, there were differences related to the number of years of employment at the primary job, with nurses who had been working for between 1 and 14 years reporting higher likelihoods of taking sick leave when not actually sick (Table 3).

### Socio-demographic predictors of negative health system consequences among moonlighters

Similar variations by province, sector, nursing category, and unit were found in the multiple regressions of health system consequences relevant to the moonlighting group only (Table 4).

**Table 4:** Multiple logistic regression of predictors of health system consequences

		Argued with doctors or other nurses	Conflicting schedules between primary and secondary jobs	Took vacation leave to do agency work or moonlighting	Took sick leave to do agency work or moonlighting	Stayed away without authority to do agency work or moonlighting	Treated patients differently in primary vs. secondary job	Any of these
								
Variable		OR (95% CI)	OR (95% CI)	OR (95% CI)	OR (95% CI)	OR (95% CI)	OR (95% CI)	OR (95% CI)
Province	Gauteng	–	–	–	–		–	–
	Eastern Cape	1.01	0.49	0.70	0.82		0.04**	0.79
		(0.58–1.75)	(0.23–1.05)	(0.32–1.55)	(0.31–2.17)		(0.00–0.36)	(0.45–1.40)
	Western Cape	0.45***	0.34***	0.46**	0.95		0.17*	0.45***
		(0.29–0.68)	(0.23–0.51)	(0.27–0.80)	(0.48–1.88)		(0.04–0.71)	(0.32–0.65)
	Free State	0.44***	1.01	1.12	0.24*		0.56	0.83
		(0.29–0.66)	(0.60–1.70)	(0.63–1.99)	(0.07–0.83)		(0.13–2.39)	(0.56–1.24)
Sex	Male		–	–				
	Female	0.82	0.73	0.68				
		(0.53–1.27)	(0.39–1.37)	(0.36–1.27)				
Age group	<25 years			–				–
	25–34 years			3.95				1.68
				(0.71–21.86)				(0.51–5.53)
	35–44 years			4.08				1.51
				(0.74–22.46)				(0.49–4.64)
	45–54 years			2.69				1.02
				(0.46–15.71)				(0.36–2.89)
	55+ years			2.18				0.99
				(0.36–13.29)				(0.28–3.48)
Sector	Public	–		–	–	–	–	–
	Private	1.16		1.09	0.44*	0.63	0.26	1.01
		(0.75–1.79)		(0.69–1.71)	(0.21–0.95)	(0.15–2.67)	(0.05–1.32)	(0.70–1.45)
	Agency	0.54*		0.20***	0.72	5.44*	0.44	0.49*
		(0.32–0.93)		(0.10–0.39)	(0.28–1.88)	(1.31–22.63)	(0.07–2.99)	(0.28–0.85)
Nursing category	Professional nurse	–		–	–			–
	Enrolled nurse	0.65		0.61*	1.66			0.69
		(0.38–1.13)		(0.40–0.94)	(0.75–3.68)			(0.47–1.01)
	Nursing assistant	0.86		0.28**	0.46			0.79
		(0.50–1.47)		(0.13–0.62)	(0.18–1.18)			(0.51–1.22)
Unit	General wards		–	–				–
	Maternity		0.64	0.94				0.70
			(0.23–1.75)	(0.41–2.15)				(0.42–1.19)
	ICU		1.08	2.76**				1.32
			(0.56–2.06)	(1.32–5.78)				(0.88–1.98)
	Theatre		1.47	1.52				1.69**
			(0.76–2.82)	(0.72–3.20)				(1.23–2.32)
	Other		0.59	1.75				1.15
			(0.30–1.16)	(0.88–3.51)				(0.81–1.62)
Years working at	Less than 1 year	–		–	–			–
primary job	1–4 years	1.67		0.88	0.72			1.16
		(0.87–3.21)		(0.48–1.59)	(0.25–2.11)			(0.71–1.88)
	5–9 years	1.40		1.04	0.44			1.38
		(0.92–2.11)		(0.49–2.24)	(0.10–1.86)			(0.88–2.17)
	10–14 years	1.75*		1.17	0.67			1.42
		(1.01–3.02)		(0.51–2.71)	(0.12–3.70)			(0.85–2.38)
	15–19 years	1.86*		1.03				1.41
		(1.07–3.24)		(0.45–2.34)				(0.78–2.56)
	20 or more years	1.35		1.05	0.11			1.23
		(0.79–2.29)		(0.56–1.97)	(0.01–1.06)			(0.75–2.03)
Constant		0.28***	0.19***	0.07**	0.08***	0.01***	0.05***	0.42
		(0.15–0.52)	(0.08–0.48)	(0.01–0.49)	(0.03–0.20)	(0.01–0.02)	(0.03–0.07)	(0.12–1.45)
Observations		1208	1299	1195	1135	1280	1280	1205
Model *p* value		<0.001	<0.001	<0.001		0.030	0.005	<0.001

Type 2 indicators in moonlighting nurses.

*** *p*<0.001

** *p*<0.01

* *p*<0.05.

In terms of geographical location and relative to Gauteng, those moonlighters from the Western Cape were less likely to report that they had conflicting schedules between primary and secondary jobs (OR: 0.34: 95% CI: 0.23–0.51); taken vacation leave to do agency work or moonlighting (OR: 0.46; 95% CI: 0.27–0.80); treated patients differently in the primary compared to the secondary job (OR: 0.17; 95% CI: 0.04–0.71) or argued with doctors or other nurses (OR: 0.45; 95% CI: 0.29–0.68). Participants from the Free State province were also less likely to report that they had taken sick leave to do agency work or moonlighting or that they argued with doctors or other nurses (Table 4).

Those working for a commercial nursing agency were more likely to report that they stayed away without authority to do agency work or moonlighting, with an odds ratio of 5.44 (95% CI: 1.31–22.63).

Relative to professional nurses, enrolled nurses, and nursing assistants were less likely to report that they had taken vacation leave to do agency work or moonlighting (Table 4).

## Discussion

In this study, 4 in 10 nurses reported moonlighting or working for a nursing agency in the year preceding the survey. South Africa's 5-year plan on human resources for health notes that ‘[i]t is common knowledge that public sector professionals “moonlight”, with or without permission, and that this reduces their productivity significantly and is a contributor to poor quality care’ (40, p. 51). Although this logic makes sense, our analysis did not find consistent statistically significant differences in self-reported health system incidents between moonlighting and non-moonlighting nurses. The bivariate analysis found that moonlighters were more likely to take sick leave when not sick (12.5% vs. 9.7%; *p*=0.011) and pay less attention to nursing work while on duty (13.2% vs. 10.3%, *p*=0.035). However, these differences did not remain statistically significant after adjusting for other socio-demographic variables in the multiple regression analysis, even though the odds ratios for these two variables were greater than 1 for moonlighters compared to non-moonlighters (Table 3).

Although the differences in these outcomes were not large enough to achieve statistical significance, it does not mean that the potential problems associated with the casualisation of the nurse workforce should be ignored in practice by hospital managers and health policy-makers. These reported health system incidents, which include taking sick leave when not sick and paying less attention to nursing work on duty, are serious. Cautionary evidence has been found in other studies. In the United Kingdom, for example, research has found that the use of temporary nursing staff (though moonlighting or agency nurses) contributes to the fatigue and burnout of permanent staff, who have to cover for or assist these temporary nurses; reduces the quality of patient care; and increases the risk of liability (27). Our study did not measure patient outcomes, but a US study also found that nursing homes that used a greater proportion of contract licensed staff were more likely to receive the worst quality deficiency ratings (29). Interestingly, the National Audit Office in the United Kingdom found that ward staff do not always report poor performance by temporary nursing staff but make sure that they do not return, hence the poor performance could be repeated elsewhere (27).

Although there is increasing policy attention to the performance of the health workforce (2, 40), the importance of dealing with fatigue among nurses appears to be a low policy priority. The finding that 51.5% of South African nurses reported that they felt too tired to work while on duty is alarming and has major implications for quality of patient care. Although not directly comparable because of different methodologies and tools used, the finding in our study is higher than those of a multicountry study where 38.1% of hospital nurses in China and 30.3% of nurses in Europe reported emotional exhaustion (8). There is well-documented evidence that nurse fatigue is a risk to patient safety and nurse well-being and contributes to negative patient outcomes and reduced job performance (8–10, 46–48). In recognition of this risk, the Registered Nurses Association of Ontario has published extensive guidelines on the prevention and mitigation of fatigue among nurses (48), and the American Institute of Medicine has highlighted the negative effects of fatigue on health care provider performance (49).

In the bivariate analysis, factors associated with nurses reporting feeling tired at work were geographical location (province), age group, marital status, public sector employment, category of nurse, and the number of years qualified as a nurse (Table 1). Interestingly, fewer moonlighters (50.5%) reported fatigue compared to non-moonlighters (52.2%). This unexpected result could be because hospital nursing managers tend to allocate fewer responsibilities to agency (moonlighting) nurses, preferring more complex nursing tasks (administration of intravenous medication) to be performed by permanent staff (50). In the multiple regression analysis, province, age younger than 35 years, public sector employment, and professional nursing category were predictors of feeling tired (Table 3). Study participants from the Eastern Cape Province and Gauteng were more likely to report feeling tired, compared to the other two provinces. Although a possible explanation for the higher rates in the Eastern Cape could be staff shortages in this more rural province with a high number of reported vacancies (40) and possibly larger workloads, more research is needed to determine the reasons for the observed provincial variation. Surprisingly, nurses older than 55 years were less likely to report feeling tired. The finding that public sector nurses were more likely to report feeling tired is not surprising in light of high patient numbers and workloads in the public sector, as the majority of South Africans are dependent on public hospitals for in-patient care (51). This is despite the fact that the same survey found higher moonlighting rates among private sector nurses, compared to those in the public sector (42). Professional nurses were also more likely to report feeling too tired to work while on duty. This could be because of their more advanced nursing skills and greater demand for their services in hospitals compared to other categories of nurses. Furthermore, professional nurses reported higher moonlighting or agency nursing rates, compared to other nursing categories (42).

The reported fatigue among nurses is exacerbated by other negative health system consequences found in this study. Among moonlighting nurses, 11.9% indicated that they had used their vacation leave to do agency work or moonlighting, contributing to fatigue. Nurses also reported unacceptably high rates of unauthorised absences leading to further understaffing, overwork, and health worker exhaustion. Of all nurses, 10.9% indicated that they had taken sick leave when not actually sick, and 5.6% had missed work without permission. These incidents were more common among moonlighting nurses who indicated that the unauthorised absences were sometimes used to do agency work or moonlighting.

A minority of nurses in the study (2.9%) reported a medico-legal incident, with category of nurse being the main predictor of reporting such an incident (Table 3). Nursing assistants were less likely to report a medico-legal incident (OR: 0.50; 95% CI: 0.26–0.93) relative to professional nurses (Table 3), again reflecting their much lower skills and type of tasks performed.

The study found that 33.7% of those who had done moonlighting or agency nursing were involved in any of the negative incidents (Table 2). There were provincial variations, which could be related to more effective management of moonlighting and agency nursing in the Western Cape and Free State. At the time of the study, Western Cape was one of two provinces that had a dedicated nursing director at the provincial level, who was tasked with the responsibility of standardisation of nursing policies, support, and monitoring of all health facilities.

There are a number of limitations of the study. As with all cross-sectional surveys, the temporal sequence between moonlighting or agency nursing and health system consequences could not be determined, leading to uncertainty as to whether these proxy indicators were causally related to moonlighting. Also, the consequences were self-reported, and we did not have objective measures of leave taken, absenteeism (staying away without authority), or medico-legal incidents. We also did not use a pretested instrument to measure fatigue. With self-reported data there is also always the possibility of social desirability bias resulting in lower disclosures of moonlighting or of negative health system consequences. The fact that the questionnaires were self-administered and anonymous provided greater privacy, which should have led to more accurate reporting of practices that are subject to social sanction. However, if moonlighting nurses were less likely to report health system consequences than non-moonlighters, it may also explain the lack of consistent statistical differences between the groups. Other limitations of the general survey are discussed in more detail in the previous article (42).

Notwithstanding these limitations, this study makes a number of important contributions. Our study is one of the first representative studies in South Africa and in Africa to examine the health system consequences of agency nursing and moonlighting – examples of casual or contingent work. The self-reported information on nurse fatigue in this large survey provides a basis for future comparisons of this aspect, which is a risk factor to patient safety and nurse well-being. The study also assisted in putting moonlighting and agency nursing on the health policy agenda in South Africa. However, further research is needed to assess the impact of moonlighting and agency nursing on more objective measures of nurse performance and ultimately on patient outcomes.

Our research has implications for health workforce policies and management and for quality of care. In South Africa, the Basic Conditions of Employment Act regulates the number of hours of employment in both the public and private health sectors (52), hence the legal framework is in place to prevent nurse fatigue. The Canadian guidelines propose the prevention and management of fatigue by allocating financial resources for infrastructure that enables health professionals to rest, recruitment and additional training facilities, appointment of additional staff, and education of all nurses about the causes and consequences of fatigue (48). South Africa's ‘Strategic Plan for Nursing Education, Training and Practice’ (41) contains a comprehensive set of recommendations that includes positive practice environments but there has been little, if any, implementation of these recommendations. Although the appointment of a Chief Nursing Officer at the beginning of 2014 is encouraging, a lot of effort is needed to overcome the implementation inertia characteristic of policy-making in South Africa (53).

In terms of moonlighting, the South African Public Service Act stipulates the conditions for additional, paid employment in the public sector (54). In theory, approval for moonlighting should only be granted if it does not impede the effective or efficient performance of the employee and, once approval is granted, implementation requires careful monitoring (40). The provincial health departments have recognised that the legal provisions are being ‘widely abused and should be much more closely managed’ (40, p. 59). Geographical location (province) explained some of the variation for the negative health system consequences, suggesting that there was better nursing management in some of the provinces. Mitigating the potential health system consequences of agency nursing and moonlighting requires decisive leadership and proactive management from the Chief Nursing Officer and hospital and nursing managers in both the public and private health sectors, rather than more legal provisions. At the same time, the national nursing association should spearhead a broader discussion on agency nursing and moonlighting and its implications for both patients and nurses. Best practice guidelines, drawing on the experience of other countries, should be developed for nurses and health facilities (33, 55).

Lastly, South Africa's emphasis on patient safety and quality of care (56) necessitates that agency nursing and moonlighting be addressed as part of the countrywide initiatives to create a quality revolution in health care.

## Conclusion

This study has investigated the negative health system consequences of agency nursing and moonlighting using a number of self-reported proxy indicators. Although we did not find consistent statistically significant differences between moonlighting and non-moonlighting nurses, the reported health system incidents are serious and further research is warranted. Although the process is complex, the casualisation of the nursing workforce cannot be viewed in isolation of South Africa's overall health system challenges, in particular its human resource challenges. In both the public and private health sectors, agency nurses are used to address nursing shortages (57). At the same time, casualisation with its concomitant shifting work patterns, an ageing nursing workforce, and a disjuncture between policies and implementation exacerbates nursing shortages. Although temporary nursing staff plays a role in dealing with actual and perceived nursing shortages, the potential negative consequences of agency nursing and moonlighting need to be counteracted through a combination of strong nursing leadership, effective management, and consultation with and buy-in from front-line nurses.

## References

[1] WHO (2010). Health system financing: the path to universal coverage.

[2] Global Health Workforce Alliance, WHO (2013). A universal truth: no health without a workforce.

[3] WHO (2010). Strategic directions for strengthening nursing and midwifery services 2011–2015.

[4] WHO AFRO (2007). WHO guidelines for implementing strategic directions for strengthening nursing and midwifery services in the African region 2007–2017.

[5] WHO (2010). Will we achieve universal access to HIV/AIDS services with the health workforce we have? A snapshot from five countries.

[6] Aiken L, Clarke S, Sloane D, Sochalski J, Silber J (2002). Hospital nurse staffing and patient mortality, nurse burnout, and job dissatisfaction. JAMA.

[7] Aiken LH, Sermeus W, Van den Heede K, Sloane DM, Busse R, McKee M (2012). Patient safety, satisfaction, and quality of hospital care: cross sectional surveys of nurses and patients in 12 countries in Europe and the United States. BMJ.

[8] You L-M, Aiken LH, Sloane DM, Liu K, He G-P, Hu Y (2013). Hospital nursing, care quality, and patient satisfaction: cross-sectional surveys of nurses and patients in hospitals in China and Europe. Int J Nurs Stud.

[9] Teng C-I, Shyu Y-IL, Chiou W-K, Fan H-C, Man Lam S (2010). Interactive effects of nurse-experienced time pressure and burnout on patient safety: a cross-sectional survey. Int J Nurs Stud.

[10] Kirwan M, Matthews A, Scott PA (2013). The impact of the work environment of nurses on patient safety outcomes: a multi-level modelling approach. Int J Nurs Stud.

[11] Ausserhofer D, Schubert M, Desmedt M, Blegen MA, De Geest S, Schwendimann R (2013). The association of patient safety climate and nurse-related organizational factors with selected patient outcomes: a cross-sectional survey. Int J Nurs Stud.

[12] Cho S, Ketefian S, Barkauskas VH, Smith DG (2003). The effects of nurse staffing on adverse events, morbidity, mortality, and medical costs. Nurs Res.

[13] Mueller M, Lohmann S, Ralf Strobl R, Boldt C, Grill E (2010). Patients’ functioning as predictor of nursing workload in acute hospital units providing rehabilitation care: a multi-centre cohort study. BMC Health Serv Res.

[14] Unruh LY, Hassmiller SB, Reinhard SC (2008). The importance and challenge of paying for quality nursing care. Policy Polit Nurs Pract.

[15] Wunderlich GS, Sloan F, Davis CK (1996). Nursing staff in hospitals and nursing homes: is it adequate?.

[16] Kurtzman ET, Corrigan JM (2007). Measuring the contribution of nursing to quality, patient safety, and health care outcomes. Policy Polit Nurs Pract.

[17] ILO (2006). Changing patterns in the world of work.

[18] Connelly CE, Gallagher DG (2004). Emerging trends in contingent work research. J Manag.

[19] Aitken RL, Manias E, Peerson A, Parker JM, Wong K (2001). Casualisation of the nursing workforce. A submission for the national review of nursing education, for the Minister for Education, Training and Youth Affairs and the Minister for Health and Aged Care.

[20] Allan C (2000). The hidden organisational costs of using non-standard employment. Pers Rev.

[21] Auer P (2006). The internationalization of employment: a challenge for a fair globalization?. Int Labour Rev.

[22] Clark PF, Stewart JF, Clark DA (2006). The globalization of the labour market for health-care professionals. Int Labour Rev.

[23] Crush J, Ulicki T, Tseane T, Van-Veuren EJ (2001). Undermining labour: the rise of sub-contracting in South African gold mines. J South Afr Stud.

[24] O'Reilly J, Trueman P, Ganderton M, Wright D (2006). Results of census of temporary nursing staff in acute hospital and foundation trusts: final report.

[25] Quinlan M, Mayhew C, Bohle P (2001). The global expansion of precarious employment, work disorganization and consequences for occupational health: a review of recent research. Int J Health Serv.

[26] Webster E (2005). Making a living, earning a living: work and employment in Southern Africa. Int Polit Sci Rev.

[27] Mercer M, Buchan J, Chubb C (2010). Flexible nursing: report for NHS professionals.

[28] Ferrinho P, Van Lerberge W, Fronteira I, Hipolito F, Biscaia A (2004). Dual practice in the health sector: review of the evidence. Hum Resour Health.

[29] Bourbonniere M, Feng Z, Intrator O, Angelelli J, Mor V, Zinn JS (2006). The use of contract licensed nursing staff in U.S. nursing homes. Med Care Res Rev.

[30] Dunton N, Gajewski B, Klaus S, Pierson B (2007). The relationship of nursing workforce characteristics to patient outcomes. Online J Issues Nurs.

[31] Roseman C, Booker JM (1995). Workload and environmental factors in hospital medication errors. Nurs Res.

[32] Alonso-Echanove J, Edwards JR, Richards MJ, Brennan P, Venezia RA, Keen J (2003). Effect of nurse staffing and antimicrobial-impregnated central venous catheters on the risk for bloodstream infections in intensive care units. Infect Control Hosp Epidemiol.

[33] National Audit Office (2006). Improving the use of temporary nursing staff in NHS acute and foundation trusts.

[34] Hurst K, Smith A (2011). Temporary nursing staff – cost and quality issues. J Adv Nurs.

[35] Berman P, Cuizon D (2004). Multiple public-private jobholding of health care providers in developing countries: an exploration of theory and evidence.

[36] Ferrinho P, Van Lerberge W, Julien MR, Fresta E, Gomes A, Dias F (1998). How and why public sector doctors engage in private practice in Portuguese-speaking African countries. Health Policy Plan.

[37] Braddy PK, Washburn TA, Caroll LL (1991). Factors influencing nurses to work for agencies. West J Nurs Res.

[38] Hughes KK, Marcantonio RJ (1991). Recruitment, retention and compensation of agency and hospital nurses. J Nurs Admin.

[39] Peerson A, Aitken RL, Manias E, Parker JM, Wong K (2002). Agency nursing in Melbourne, Australia: a telephone survey of hospital and agency managers. J Adv Nurs.

[40] DOH (2011). Human Resources for Health South Africa: HRH strategy for the health sector: 2012/13 – 2016/17.

[41] DOH (2013). Strategic Plan for Nursing Education, Training and Practice 2012/13 – 2016/17.

[42] Rispel LC, Blaauw D, Chirwa T, de Wet K (2014). Factors influencing agency nursing and moonlighting among nurses in South Africa. Glob Health Action.

[43] Greenacre M, Blasius J (2006). Multiple correspondence analysis and related methods.

[44] Howe LD, Hargreaves JR, Huttly SR (2008). Issues in the construction of wealth indices for the measurement of socio-economic position in low-income countries. Emerg Themes Epidemiol.

[45] Rispel LC, Chirwa T, Blaauw D (2014). Does moonlighting influence South African nurses’ intention to leave their primary jobs?. Glob Health Action.

[46] Clarke SP, Donaldson NE, Hughes RG (2008). Nurse staffing and patient care quality and safety: chapter 25. Patient safety and quality: an evidence-based handbook for nurses.

[47] Scott LD, Hwang WT, Rogers AE (2006). The impact of multiple care giving roles on fatigue, stress, and work performance among hospital staff nurses. J Nurs Adm.

[48] Registered Nurses’ Association of Ontario (2011). Preventing and mitigating nurse fatigue in health care: healthy work environments best practice guidelines.

[49] IOM (2001). Crossing the quality chasm: a new health system for the 21st century.

[50] Rispel LC, Moorman J (2015). The indirect costs of agency nurses: a case study in two public sector hospitals in South Africa. Glob Health Action.

[51] Blecher M, Kollipara A, de Jager P, Zulu N, Padarath A, English R Health financing. South African health review 2011.

[52] Republic of South Africa (2013). Basic Conditions of Employment Act (as amended).

[53] Rispel L, Moorman J, Daniel J, Naidoo P, Pillay D, Southall R Health policy reforms and policy implementation in South Africa: a paradox?. New South African review 3: the second phase-tragedy or farce?.

[54] Public Service Commission (2007). Remunerative work outside the public service: an investigation undertaken in the Gauteng provincial health sector.

[55] National Audit Office (2006). Good practice in managing the use of temporary nursing staff.

[56] RSA (2013). National Health Amendment Act No. 12 of 2013. Government Gazette.

[57] Rispel L, Armstrong S, Blaauw D, Chirwa T, Engelbrecht M, de Wet K (2011). The nature and health system consequences of casualisation, agency nursing and moonlighting in South Africa.

# Sub article: Utilisation and costs of nursing agencies in the South African public health sector, 2005–2010

**DOI:** 10.3402/gha.v7.25053

**Published:** 2014-12-22

**Authors:** Laetitia C. Rispel, George Angelides

**Affiliations:** 1 Centre for Health Policy & Medical Research Council Health Policy Research Group, School of Public Health, Faculty of Health Sciences, University of the Witwatersrand, Johannesburg, South Africa; 2 Gauteng Department of Health, Johannesburg, South Africa

**Keywords:** nursing agency, costs, utilisation, health workforce, South Africa

## Abstract

**Background:**

Globally, insufficient information exists on the costs of nursing agencies, which are temporary employment service providers that supply nurses to health establishments and/or private individuals.

**Objective:**

The aim of the study was to determine the utilisation and direct costs of nursing agencies in the South African public health sector.

**Methods:**

A survey of all nine provincial health departments was conducted to determine utilisation and management of nursing agencies. The costs of nursing agencies were assumed to be equivalent to expenditure. Provincial health expenditure was obtained for five financial years (2005/6–2009/10) from the national Basic Accounting System database, and analysed using Microsoft Excel. Each of the 166,466 expenditure line items was coded. The total personnel and nursing agency expenditure was calculated for each financial year and for each province. Nursing agency expenditure as a percentage of the total personnel expenditure was then calculated. The nursing agency expenditure for South Africa is the total of all provincial expenditure. The 2009/10 annual government salary scales for different categories of nurses were used to calculate the number of permanent nurses who could have been employed in lieu of agency expenditure. All expenditure is expressed in South African rands (R; US$1 ∼ R7, 2010 prices).

**Results:**

Only five provinces reported utilisation of nursing agencies, but all provinces showed agency expenditure. In the 2009/10 financial year, R1.49 billion (US$212.64 million) was spent on nursing agencies in the public health sector. In the same year, agency expenditure ranged from a low of R36.45 million (US$5.20 million) in Mpumalanga Province (mixed urban-rural) to a high of R356.43 million (US$50.92 million) in the Eastern Cape Province (mixed urban-rural). Agency expenditure as a percentage of personnel expenditure ranged from 0.96% in KwaZulu-Natal Province (mixed urban-rural) to 11.96% in the Northern Cape Province (rural). In that financial year, a total of 5369 registered nurses could have been employed in lieu of nursing agency expenditure.

**Conclusion:**

The study findings should inform workforce planning in South Africa. There is a need for uniform policies and improved management of commercial nursing agencies in the public health sector.

The global quest for universal health coverage has once again put the spotlight on the centrality of the health workforce to achieving this goal (1). New modelling estimates suggest a global deficit of around 12.9 million nurses, midwives, and physicians by 2035 (1). In 2010, it was estimated that there were more than 100,000 vacancies – a proxy for staff shortages – for all categories of health care professionals in the South African public health sector (2). Nurses constituted the vast majority of these vacancies (2).

Worldwide, the management of nursing shortages in health facilities is a major problem (3). In Australia, Canada, the United Kingdom, and the United States of America, temporary nursing staff (e.g. agency or casual nurses) have been used to fill the gap created by a lack of full-time, permanent nurses in the health care system (3–8). In South Africa, anecdotal evidence suggests a growth in commercial nursing agencies, which are temporary employment service providers that supply different categories of nurses to health establishments and/or private individuals (9). A large, representative cross-sectional study conducted in 2010 found that one in every 11 nurses worked for a commercial nursing agency and that 37.8% of study participants engaged in agency nursing in the year preceding the survey (10). These nurses are employed through nursing agencies.

Much of the literature on nursing agencies is concerned with high-income countries (5, 6, 8, 11–16). A key focus and concern of the literature have been on the management of temporary or agency staff (11, 12, 14, 15, 17, 18), or the negative implications of temporary staff for nursing managers, communication, patient care, and/or health service delivery (19–21). In recent years, Canada, the United Kingdom, and the United States have studied their nursing workforce profiles, including the number of casual and/or agency nurses, in an attempt to assist with improved health workforce planning and forecasting (22–25). The UK National Audit Office has a monitoring system on the use and management of all temporary nursing staff in acute hospital and foundation trusts, and trend information is available over a period of time (18, 26, 27).

There are few empirical studies on nursing agencies (3, 6, 8), and these tend to be descriptive, sometimes lacking the methodological rigour needed to make generalisable conclusions. Globally, insufficient information exists on the costs of agency nursing. The UK National Audit Office has commissioned several studies, including a census of National Health Service (NHS) Trusts to examine the demand for temporary staff (including agency staff), their utilisation, the costs of procurement, and the impact of initiatives to improve quality and expenditure information (18, 27). A Melbourne study on agency nursing found high utilisation of agency nurses in response to problems of recruitment and retention of nurses in specialty areas, busy and fluctuating caseload units, and lack of permanent night duty staff (8). However, there are methodological limitations as it was a telephone survey, financial data were posted, and the agency response rate was poor (8). In South Africa, expenditure information is more readily available, particularly in the private health sector (28), but the costs of nursing agencies in the public sector have not been well researched.

Cost information is important for the management of temporary staff in a health facility, for shaping policy responses, monitoring trends, and/or benchmarking the performance of nursing agencies in the health system (29, 30). Costing data also allow for improved workforce planning, including comparisons of quality and performance between agency staff and permanent staff (26). In light of this and the dearth of empirical information, the aim of this study was to determine utilisation and costs of nursing agencies in the South African public health sector.

## Methods

The study setting was the nine provincial health departments in South Africa, which were classified as follows: urban (Gauteng and Western Cape), rural (Limpopo, Northern Cape, and North West), and mixed urban-rural (Eastern Cape, Free State, KwaZulu-Natal, and Mpumalanga). The study was approved by the Human Research Ethics Committee (Medical) of the University of the Witwatersrand in Johannesburg. The provincial health authorities also provided approval for the study.

A *survey* was conducted among all nine provincial health departments in South Africa to determine the utilisation and management of nursing agencies. The survey aimed to develop an understanding of the drivers of agency nurse utilisation and to explore the policies on, and management of, nursing agencies. Four face-to-face interviews and three telephonic interviews were conducted, using a semi-structured interview schedule. In the case of one province, the human resource manager in the province completed the interview schedule and faxed it back to the researchers. The interview schedule focussed on utilisation of nursing agencies in the province; the type of health facilities or services that utilise agency nurses; policy frameworks that guide the use of nursing agencies, including the existence of a specific provincial policy; and the perceived advantages and disadvantages of nursing agencies. The information from the interviews was coded and analysed using thematic content analysis.

A cost analysis of nursing agencies was done for the 5-year period from 2005 until 2010. We assumed that the direct costs of nursing agencies were equivalent to *expenditure* on nursing agencies. Data on provincial health expenditure were obtained for five financial years (2005/6–2009/10) from the national transversal Basic Accounting System (BAS) database (i.e. official government statistics). The financial year in government commences on 1 April each year and ends on 31 March of the following year. The data were exported into Microsoft Excel in order to facilitate uniform coding of the expenditure information and to do trend analysis across the entire period (2005/06 to 2009/10). Each financial year was put into a separate worksheet. The number of expenditure line items ranged from 30,595 in the 2005/6 financial year to 34,978 in the 2009/10 financial year, totalling 166,466 line items for coding over the 5-year period. Each line item was coded meticulously, depending on the type of expenditure, and similar items were grouped and aggregated. The coding also took into account the province or health facility where the expenditure occurred. Once each item was coded, cross-tabulations and calculations were done to ensure that the same total expenditure was obtained for each provincial health department, thus ensuring both validity and reliability of the study results.

Overall provincial health expenditure consists of all expenditure items, including compensation of employees, goods and services (operational expenditure), transfer payments (to municipalities or non-governmental organisations), and capital expenditure. Personnel expenditure, also called compensation of employees, consists of salaries, staff benefits, and overtime payments. Nursing agency expenditure consists of all expenses paid to commercial nursing agencies (including administration fees) and is paid from the goods and services budget of each provincial health department.

For each of the nine provinces, the total personnel and nursing agency expenditure was calculated for each financial year. Agency nursing expenditure as a percentage of the total personnel expenditure was then calculated, for each province and for each financial year. The nursing agency expenditure for all provinces was totalled to arrive at agency expenditure for South Africa for each financial year, and over the 5-year period.

In order to calculate the number of full-time-equivalent (FTE) nurses who could have been employed for the given nursing agency expenditure for that province, the 2009/10 government salary scales for different categories of nurses (professional, enrolled, and auxiliary nurses) were obtained. The median salary for each nursing category was calculated. Assuming standard benefits such as health insurance and housing subsidy, 30% was added to the amount. This amount for each nursing category was then used to calculate the number of FTE nurses. All expenditure is expressed in South African rand (R; US$1 ∼ R7, 2010 prices).

## Results

### Utilisation of nursing agencies, policies and management

Eight out of nine provincial health departments responded to the provincial survey on nursing agencies (89% response rate). The team tried unsuccessfully for 18 months to get a response from the Northern Cape Provincial Health Department.

Table 1 shows the reported utilisation of nursing agencies in the South African public health sector.

**Table 1:** Utilisation and management of nursing agencies in the South African public health sector

Province	Nursing agency utilisation	Existence of specific provincial health policy framework	Advantages	Disadvantages
Eastern Cape	Yes, in tuberculosis and public-private partnership hospitals	Unsure	Agencies deploy staff to geographical areas or health facilities where it is difficult to find staff.	Agencies not well regulated Many agency nurses not registered with the South African Nursing Council
Free State	Yes	Unsure	Staff supplementation Savings on personnel budget as they are paid from the goods and services budget	Agencies do not send nurses with required skills. Some agency nurses not familiar with the public health sector. Nurses abuse the system by working through agencies while on leave at the primary employer.
Gauteng	Yes, contract with 10 agencies Mostly used in large hospitals and specialised areas (e.g. critical care)	Yes – provincial nursing agency policy	Assist with staff shortages in specialty areas, such as critical care units	Managers use nursing agencies to manage absenteeism. Agencies send inexperienced nurses or nurses without specialised skills. Contractual obligations not enforced by hospital management. Possible corruption and collusion between agencies, managers, and individual nurses
Kwazulu-Natal	Stopped utilisation of nursing agencies due to budgetary constraints	No Used government supply chain management policy	–	Nurses employed by provincial Department of Health moonlighted via agencies in their hospitals of employment.
Limpopo	No	Not applicable	Not applicable	Not applicable
Mpumalanga	Yes, contract with 2 agencies	No	Able to provide efficient services	Employees compare the wages received from agencies with departmental salaries, and that creates conflict.
North West	No	Not applicable	Not applicable	Not applicable
Western Cape	Yes, contract with 9 agencies Mostly used in large hospitals and specialised areas	Yes, public sector policies on remunerative work outside the public service and provincial human resource management	Able to provide staff at short notice	High costs Inexperienced nurses Sub-optimal quality of care

The provincial survey (Table 1) found that budgetary constraints have either prevented the use of nursing agencies (North West, Eastern Cape, and Limpopo) or led to the termination (KwaZulu-Natal) or the restricted and/or controlled use of nursing agencies (Free State, Gauteng, Mpumalanga, and Western Cape). Gauteng and Western Cape Provinces reported specific policy frameworks that guide the utilisation of nursing agencies. In Free State and Mpumalanga, the generic government procurement policy was used. The perceived advantages of utilising nursing agencies were that they complemented the existing number of staff, especially in specialised areas, and cost savings on the personnel budget, particularly after the implementation of the Occupation-Specific Dispensation (OSD), a financial incentive for health professionals in the public service (31). Reported disadvantages of nursing agencies were: the failure of agencies to supply the required nursing skills; inappropriate use of agencies, often to manage permanent staff absenteeism; lack of enforcement of contractual obligations; possible corruption and collusion between agencies, managers, and nurses; and potential abuse of the system when provincial government nurses do moonlighting through an agency.

### Total nursing agency expenditure in the South African public health sector, 2005–2010

Figure 1 shows the overall nursing agency expenditure in the South African public health sector. Expenditure ranged from R914.29 million (US$130.61 million) in the 2005/6 financial year to a peak of R1.53 billion (US$218.22 million) in 2007/8. In the 2009/10 financial year, R1.49 billion (US$212.64 million) was spent on nursing agencies. The total amount spent on nursing agencies in the South African public health sector for the 5-year study period was R6.47 billion (US$924.01 million).

**Fig. 1:** Trends in total nursing agency expenditure in the South African public health sector, 2005–2010. Source: South Africa National Treasury, national transversal Basic Accounting System (BAS). R = South African rands.

### Trends in nursing agency expenditure

Table 2 and Fig. 2 show the trends in nursing agency expenditure for each of the nine provinces over the 5-year period. As can be seen from Table 2, in 2009/10, agency expenditure ranged from a low of R36.45 million (US$5.21 million) in Mpumalanga Province to a high of R356.43 million (US$50.92 million) in the Eastern Cape Province. Fig. 2 shows that there were erratic expenditure patterns in the majority of provinces, and unexplained peaks in Gauteng in 2007/8, in North West in 2008/9, and in Northern Cape in 2009/10.

**Fig. 2:** Trends in agency nursing expenditure by province, 2005–2010. Source: South Africa National Treasury, national transversal Basic Accounting System (BAS). EC = Eastern Cape; FS = Free State; GP = Gauteng; KZN = KwaZulu Natal; LP = Limpopo; MP = Mpumalanga; NC = Northern Cape; NW = North West; WC = Western Cape; R = South African rands.

**Table 2:** Trends in nursing agency expenditure in the South African public health sector, 2005–2010.

Province	2005/6	2006/7	2007/8	2008/9	2009/10
Eastern Cape	432,799,564	368,740,527	384,777,196	364,378,884	356,429,449
Free State	52,395,839	67,101,223	52,417,250	36,769,318	85,968,276
Gauteng	110,972,166	291,240,036	450,566,358	296,302,273	229,782,134
KwaZulu-Natal	31,192,138	25,542,244	121,060,152	119,355,192	118,437,227
Limpopo	6,354,945	11,098,524	78,030	28,030,416	87,177,905
Mpumalanga	39,818,398	41,153,298	102,911,333	7,859,761	36,445,893
Northern Cape	6,318,734	12,557,108	12,961,058	11,042,380	178,919,686
North West	0	62,684,538	184,306,034	361,680,916	177,014,047
Western Cape	234,437,117	223,761,383	218,497,181	208,431,408	218,279,792
TOTAL	914,288,902	1,103,878,880	1,527,574,592	1,433,850,547	1,488,454,407

Source: South Africa National Treasury, national transversal Basic Accounting System (BAS).

### Nursing agency expenditure as a percentage of total provincial health expenditure

Table 3 shows trends in nursing agency expenditure as a percentage of total provincial health expenditure.

**Table 3:** Trends in nursing agency expenditure as a percentage of overall provincial health expenditure

Province	2005/6 (%)	2006/7 (%)	2007/8 (%)	2008/9 (%)	2009/10 (%)
Eastern Cape	6.62	4.73	4.77	3.45	2.93
Free State	1.69	1.99	1.36	0.83	1.63
Gauteng	1.04	2.43	3.29	1.80	1.19
KwaZulu-Natal	0.29	0.21	0.79	0.68	0.53
Limpopo	0.14	0.19	0.00	0.35	0.96
Mpumalanga	1.44	1.30	2.84	0.18	0.63
Northern Cape	0.57	0.95	0.64	0.63	8.06
North West	0.00	4.64	4.79	8.14	3.44
Western Cape	3.97	3.38	2.82	2.33	2.03

Source: South Africa National Treasury, national transversal Basic Accounting System (BAS).

As can be seen from Table 3, in Limpopo (rural) and KwaZulu-Natal (mixed urban-rural) provinces, nursing agency expenditure was less than 1% of overall health spending for the entire study period. Both Eastern Cape (mixed) and Western Cape (urban) showed a 50% decline in nursing agency expenditure as a percentage of overall health spending between the beginning and end of the study period. In Gauteng (urban), there were increases between 2005/6 and 2007/8, with declines in the 2008/9 and 2009/10 financial years. Free State (mixed) appears to have managed to keep nursing agency expenditure to less than 2% of overall health spending. There were large fluctuations in the rural provinces of Northern Cape and North West, where nursing agency expenditure as a percentage of overall provincial spending was 8.06% in the Northern Cape in 2009/10 and 8.14% in North West in 2008/9.

### Nursing agency expenditure as a percentage of personnel expenditure

Table 4 illustrates trends in nursing agency expenditure as a percentage of personnel expenditure in each province for the study period.

**Table 4:** Nursing agency expenditure as a percentage of personnel expenditure

Province	2005/6 (%)	2006/7 (%)	2007/8 (%)	2008/9 (%)	2009/10 (%)
Eastern Cape	12.56	9.56	8.40	6.02	4.89
Free State	2.82	3.33	2.23	1.29	2.68
Gauteng	2.38	5.36	6.90	3.61	2.28
KwaZulu-Natal	0.52	0.38	1.39	1.19	0.96
Limpopo	0.23	0.34	0.00	0.60	1.63
Mpumalanga	2.67	2.40	5.15	0.31	1.23
Northern Cape	1.21	1.84	1.63	1.25	11.96
North West	0.00	6.75	–a	15.13	6.44
Western Cape	7.86	6.53	5.27	4.28	3.89

a Unable to calculate because of missing data.

Source: South Africa National Treasury, national transversal Basic Accounting System (BAS).

Eastern Cape and Western Cape Provinces show more than 50% declines in nursing agency expenditure as a percentage of personnel expenditure over the study period. In Northern Cape, nursing agency expenditure constituted 11.96% of personnel spending in the 2009/10 financial year, whereas in North West, agency expenditure was 15.13% of the personnel expenditure in the 2008/9 financial year. In Gauteng, nursing agency expenditure as a percentage of personnel spending showed a similar pattern to overall spending, with increases between 2005/6 and 2007/8, and declines in the 2008/9 and 2009/10 financial years. There was no consistent spending pattern in the other provinces.

### Opportunity costs of nursing agency expenditure

In 2009/10, the annual salary package of a professional nurse (4 years of training) was R277,226 (US$40,000), an enrolled nurse (2 years of training) earned an annual salary of R154,471 (US$22,000), and an auxiliary nurse (1 year of training) earned an annual salary of R119,646 (US$17,000). Using these salary packages, Table 5 shows the number of nurses of each category who could have been employed in lieu of nursing agency expenditure for the 2009/10 financial year.

**Table 5:** Opportunity costs of nursing agency expenditure, 2009/10

Province	Professional nurses	Enrolled nurses	Auxiliary nurses
Eastern Cape	1286	2307	2979
Free State	310	557	719
Gauteng	829	1488	1921
Kwazulu-Natal	427	767	990
Limpopo	314	564	729
Mpumalanga	131	236	305
Northern Cape	645	1158	1495
North West	639	1146	1479
Western Cape	787	1413	1824
South Africa	5369	9636	12,441

Data sources: South Africa National Treasury, national transversal Basic Accounting System (BAS). Annual salary packages for different categories of nurses obtained from the Department of Public Service and Administration.

As can be seen from Table 5, in 2009/10, a total of 5369 professional (registered) nurses could have been employed in the South African public health sector. Alternatively, nursing agency expenditure could have funded 9636 enrolled nurses or 12,441 auxiliary nurses.

## Discussion

The provincial survey revealed wide variations in the utilisation and management of nursing agencies across the nine provinces (Table 1), despite the growth in public sector staff numbers in the last decade (32). In the survey, North West and Limpopo Provinces reported that there was no utilisation of nursing agencies, and therefore no need for policies and/or the management of these agencies. However, this was contradicted by the costing study as expenditure was recorded in all provincial health departments. These findings on utilisation could reflect a lack of knowledge on the part of the respondents who were interviewed. On the other hand, the findings point to the disjuncture and lack of communication between human resource and finance departments at the head offices of provincial health departments, and between the head offices (centre) and lower level health facilities. Other studies have also pointed to the problems of sub-optimal communication and ‘silo’ functioning within provincial health departments in South Africa (32, 33).

The costing study found that nursing agency expenditure in the South African public sector ranged from R914.29 million (US$130.61 million) in the 2005/6 financial year to R1.49 billion (US$212.64 million) in the 2009/10 financial year. This total amount of R6.47 billion (US$924.01 million) that was spent on nursing agencies for the 5-year study period was more than the 2009/10 provincial health budget of Free State, Mpumalanga, Northern Cape, or North West Province (34). We could not find comparable studies of nursing agency expenditure in other low- and middle-income settings. In England, a 2004/5 census of temporary nursing staff in acute NHS hospital and foundation Trusts found that the Trusts spent a total amount of £238 million (pounds Sterling) on *agency* nursing staff during 2004/5, and £773 million on all temporary nursing staff (35). However, there are vast differences in the organisation of the health care system, staffing needs, the quantum of health budgets, and/or health expenditure between England and South Africa; hence, the findings are not comparable.

The costing study found that each of the nine provincial health departments spent considerable sums of money on nursing agencies that supply temporary staff (Table 2 and Fig. 2). There were large variations in nursing agency expenditure across the provincial health departments as well as in nursing agency expenditure as a percentage of total provincial health expenditure (Table 3). The latter ranged from 0.53% in KwaZulu-Natal to 8.06% in the Northern Cape Province. These geographical variations in expenditure on temporary nursing staff were also found in the 2006 report of the National Audit Office in England (18) and a more recent newspaper investigation on NHS expenditure on temporary nursing staff (36).

In the 2009/10 financial year, nursing agency expenditure as a percentage of personnel expenditure ranged from 0.96% in KwaZulu-Natal to 11.96% in Northern Cape. The reasons for the variations in expenditure are not clear. In the provincial survey, KwaZulu-Natal indicated that budgetary constraints prevented them from utilising nursing agencies. This could explain the relatively low percentage of nursing agency expenditure, relative to personnel spending. Northern Cape did not respond to the survey, thus its reasons for the utilisation of nursing agencies could not be elicited. A study in Melbourne, Australia, found that expenditure on agency staff ranged from 5 to 10% of the monthly staffing budget, but this figure was as high as 35% in some institutions (37). In England, nursing expenditure as a percentage of the nursing workforce expenditure was 3% in 2004/5 (18). Notwithstanding the report from North West Province that they did not utilise nursing agencies, in the 2008/9 financial year, nursing agency expenditure constituted 15.13% of the personnel expenditure. This was the highest proportion of provincial personnel spending on temporary or casual staff. There are several possible reasons that could explain this figure. Firstly, it could be a financial transaction error on the part of the North West Province, despite the stringent requirements of the Public Finance Management Act (38). Secondly, the utilisation of nursing agencies could be an indication of staff shortages in the many rural health facilities in the province. This reason is corroborated by an estimated 743 vacancies for all categories of nurses in 2010 (2). Thirdly, nursing agencies are paid from the goods and services budget, and not from the personnel budget. Consequently, the spending on nursing agencies could have been a mechanism to prevent or hide overspending on the personnel budget.

There are several limitations of the study. In the costing study, we assumed that direct costs were equal to expenditure. We also used public sector vacancies as a proxy for staff shortages, which is reasonable in the absence of national staffing norms. However, there is no standard definition of a ‘vacancy’, and in some instances a vacancy could be measured against an old, outdated organisational structure or against the available budget (32). Further-more, the national human resource plan was based on modelling estimates, and there was no empirical assessment of health workforce requirements against health need, itself a contested concept (33). Thirdly, we assumed a zero-sum trade-off between nurse agency costs and permanent staff employment, which may not hold in all instances. For example, agency nurses could be used to manage the cost of variations in patient demand, thus resulting in efficiency gains because of the reduction in fixed salary costs. We also do not have comparative data on the expenditure on a full-time vacancy filled by the public sector and the expenditure on a part-time equivalent filled from the agency.

The study also relied on self-reported information from senior government officials, which was shown to be inaccurate in at least two of the provinces. Although financial statistics tend to be more accurate than other information, the study used official government statistics that the provincial health departments submitted to the National Treasury, and data quality remains a challenge.

Notwithstanding these limitations, the study has numerous strengths. The study is novel in combining and contrasting information obtained through a provincial survey, with a detailed analysis and interpretation of expenditure on nursing agencies, and the trends in such expenditure over a 5-year period. A major strength of the study is its national focus, thus making an important contribution to the knowledge and understanding of the expenditure, management, and utilisation of nursing agencies in the South African public health sector. The study provides empirical evidence of the extensive utilisation of temporary agency staff in the South African public health sector, which is illustrated by the high expenditure on nursing agencies in all provinces. An important finding of the study is that 5369 professional or registered nurses with 4 years of training could have been employed in the South African public health sector in 2009/10 in lieu of nursing agency expenditure. The study also illustrates the usefulness of a trend analysis of routine financial data, and the potential of such analysis to inform policy or decision making.

Notwithstanding these limitations, the study has numerous strengths. The study is novel in combining and contrasting information obtained through a provincial survey, with a detailed analysis and interpretation of expenditure on nursing agencies, and the trends in such expenditure over a 5-year period. A major strength of the study is its national focus, thus making an important contribution to the knowledge and understanding of the expenditure, management, and utilisation of nursing agencies in the South African public health sector. The study provides empirical evidence of the extensive utilisation of temporary agency staff in the South African public health sector, which is illustrated by the high expenditure on nursing agencies in all provinces. An important finding of the study is that 5369 professional or registered nurses with 4 years of training could have been employed in the South African public health sector in 2009/10 in lieu of nursing agency expenditure. The study also illustrates the usefulness of a trend analysis of routine financial data, and the potential of such analysis to inform policy or decision making.

The study findings have implications for quality of care, human resource planning and management, and health policy development in South Africa. The 2010 national core standards (39) and the 2013 promulgation of the National Health Amendment Act underscore the importance of quality of care in the country (40). The domain of *patient safety, clinical governance, and clinical care* in the national core standards focusses on ‘how to ensure quality nursing and clinical care and ethical practice, and reduce unintended harm to health care users or patients in identified cases of greater clinical risk’ (39, p. 11). Although the utilisation of temporary agency nurses is a short-term solution to staffing shortages, there is a growing body of evidence that casual or temporary staffing contributes to poor quality of patient care (41–44). Several US studies have found statistically significant associations between the employment of agency nurses and health care deficiencies in nursing homes (41), hospital medication errors (43), and the risk of bloodstream infections among patients with central venous catheters in intensive care units (44). In the United Kingdom, it was also found that temporary staffing could undermine the quality of patient care (18). However, a study of 605 UK general and specialist wards between 2004 and 2009 found no differences in quality scores between temporary and permanent nursing staff (45). Nonetheless, the UK Department of Health has stated that replacing temporary staff with experienced permanent staff leads to increased productivity and better patient care (18). In South Africa, we could not find any studies on the association between the use of agency nurses and quality of care. However, a 2010 survey found high moonlighting rates in critical care units in both the public and private health sectors in South Africa (10), suggesting extensive utilisation of temporary nurses. Hence, similar issues of quality of care could be found in South Africa.

The management of human resources is a key aspect of quality of care as it enables the delivery of safe and effective patient care (1, 39). Hence, the study findings also have implications for human resource planning and management. The provincial survey found that there is no national policy on the management and utilisation of nursing agencies. There is global evidence that hiring agency nurses costs up to three times more than permanent staff (18, 23, 24, 27, 45). Although the utilisation of temporary staff is influenced by efficiency measures, staff shortages, and changing patient numbers and acuity, the demand for temporary nursing staff is also shaped by poor staff planning, staff absenteeism, and lack of involvement of nurses in decision making (3, 27, 45, 46).

In South Africa, there is a policy vacuum on commercial nursing agencies. Until 2005, these agencies were required to register with the South African Nursing Council (9), but the National Health Act classified nursing agencies as health establishments under the jurisdiction of the Department of Health (47). Although the 5-year strategy on nursing education, training, and practice, launched in 2013, recommended the urgent development and implementation of regulations for nursing agencies (48), the draft regulations were vague and unlikely to lead to better control of nursing agencies. The progress has since stalled. The experience of regulating nursing agencies in other countries such as the United Kingdom is instructive. In theUKminimum standards and regulations on nursing agencies aim to ensure ethically and legally compliant service delivery (26). Its national nursing association has developed a complementary set of guidelines for employers, nursing agencies, and agency nurses that summarise regulatory requirements and induction processes to meet minimum standards (49, 50).

Both the South African 5-year strategic plans on human resources for health (2) and on nursing education, training, and practice (36) recommend policy development or improved management of nursing agencies, albeit in a cursory manner. The finalisation of regulations on commercial agencies and the development of good practice guides are critical. Such regulations and practice guides could draw from the UK experience (18, 26) and must address issues of planning for the use of agency nurses, controlling demand, appointing nursing agencies, using temporary nursing staff in an efficient and effective manner, and monitoring expenditure and the performance of all nurses.

## Conclusion

This study has highlighted the need to plan strategically for the use of temporary agency nurses, and to monitor nursing agency expenditure, as part of a comprehensive workforce plan. The planned health reforms towards universal health coverage in South Africa necessitate proactive health workforce planning to improve population health, ensure the delivery of quality care, and align expenditure to budgets.

Notwithstanding the size and complexity of the health sector, implementation of nursing agency regulations and good practice guides must be a priority for the chief nursing officer. This is in light of the well-described gaps between policy and legislation on the one hand, and implementation and practice translation on the other hand (33, 51). Although the development of legislation, policies, and guidelines is important, the Health Ministry should also pay sufficient attention to the management of change, and ensure consultation with the provincial health departments (as implementing agencies) and front-line nurses. Finally, on-going monitoring of nursing agencies is important, tin order to enhance public accountability.

## References

[1] Global Health Workforce Alliance, WHO (2013). A universal truth: no health without a workforce.

[2] DOH (2011). Human Resources for Health South Africa: HRH strategy for the health sector: 2012/13–2016/17.

[3] Massey L, Esain A, Wallis M (2009). Managing the complexity of nurse shortages: a case study of bank and agency staffing in an acute care Trust in Wales, UK. Int J Nurs Stud.

[4] Aiken L (1989). Breaking the shortage cycle. Am J Nurs.

[5] Aitken RL, Manias E, Peerson A, Parker JM, Wong K (2001). Casualisation of the nursing workforce.

[6] Braddy PK, Washburn TA, Caroll LL (1991). Factors influencing nurses to work for agencies. West J Nurs Res.

[7] Buchan J, Edwards N (2000). Nursing numbers in Britain: the argument for workforce planning. Br Med J.

[8] Peerson A, Aitken RL, Manias E, Parker JM, Wong K (2002). Agency nursing in Melbourne, Australia: a telephone survey of hospital and agency managers. J Adv Nurs.

[9] Republic of South Africa Nursing Act No. 50 of 1978.

[10] Rispel LC, Blaauw D, Chirwa T, de Wet K (2014). Factors influencing agency nursing and moonlighting among nurses in South Africa. Glob Health Action.

[11] Adams B (1989). Agency nursing: the hospital response. J Nurs Adm.

[12] Hughes KK, Marcantonio RJ (1991). Recruitment, retention and compensation of agency and hospital nurses. J Nurs Adm.

[13] Jackson BS (1989). Agency nursing: costs and quality [Opinion]. J Nurs Adm.

[14] Manias E, Aitken R, Peerson A, Parker J, Wong K (2003). Agency-nursing work: perceptions and experiences of agency nurses. Int J Nurs Stud.

[15] Manion J, Reid S (1989). Managing temporary staffing services: selecting the right agency. Nurs Econ.

[16] Muir J (1988). Moonlighting. Nurs Times.

[17] Manias E, Aitken R, Peerson A, Parker J, Wong K (2003). Agency nursing work in acute care settings: perceptions of hospital nursing managers and agency nurse providers. J Clin Nurs.

[18] National Audit Office (2006). Improving the use of temporary nursing staff in NHS acute and foundation trusts.

[19] Batch M, Barnard A, Windsor C (2009). Who's talking? Communication and the casual/ part-time nurse: a literature review. Contemp Nurse.

[20] Creegan R, Duffield C, Forrester K (2003). Casualisation of the nursing workforce in Australia: driving forces and implications. Aust Health Rev.

[21] Halcomb EJ, Andrew S, Peters K, Salamonson Y, Jackson D (2009). Casualisation of the teaching workforce: implications for nursing education. Nurs Educ Today.

[22] Aiken LH, Cheu R (2008). Nurse workforce challenges in the United States: implications for Policy.

[23] Baumann A, Keatings M, Holmes G, Oreschina E, Fortier V (2002). Better data, better decisions: a profile of the nursing workforce at Hamilton Health Sciences Corporation, 2002–2003.

[24] Baumann A, Kolotylo C (2010). Forecasting future workforce demand: a process evaluation.

[25] Buchan J, Seccombe I (2009). Difficult times, difficult choices: the UK nursing labour market review 2009.

[26] National Audit Office (2006). Good practice in managing the use of temporary nursing staff.

[27] O'Reilly J, Trueman P, Ganderton M, Wright D (2006). Results of census of temporary nursing staff in Acute Hospital and Foundation Trusts: final report.

[28] Brannigan E (2008). Managing the use of nursing agencies: experience from Netcare. Presented at the RESON consultative workshop entitled: exploring nursing policies, practice and management in South Africa, Airport Grande Hotel, Boksburg.

[29] Hendricks J, Baume P (1997). The pricing of nursing care. J Adv Nurs.

[30] McCloskey JC, Gardner DL, Johnson M (1987). Costing out nursing services: an annotated bibliography. Nurs Econ.

[31] Department of Public Service and Administration (2007). Implementation of the Occupational Specific Dispensations (OSDs) for the occupations professional nurse, staff nurse and nursing assistants in the public service-File 18/2/P.

[32] Integrated Support Teams (2009). Review of health over-spending and macro-assessment of the public health system in South Africa: Consolidated Report.

[33] Rispel LC, Barron P (2012). Valuing human resources: key to the success of a National Health Insurance System. Dev South Afr.

[34] National Treasury (2009). Provincial budgets and expenditure review, 2005/06 – 2011/12.

[35] O'Reilly J, Trueman P, Ganderton M, Wright D (2006). Results of census of temporary nursing staff in Acute Hospital and Foundation Trusts: final report.

[36] Moore A (2013). Rising temporary nursing costs.

[37] Aitken RL, Manias E, Peerson A, Parker JM, Wong K (2002). Agency nursing in metropolitan Melbourne: final Research Report.

[38] Republic of South Africa (1999). The Public Finance Management Act No. 1 of 1999.

[39] DOH (2010). National Core Standards for Health Establishments in South Africa.

[40] RSA (2013). National Health Amendment Act No. 12 of 2013. Government Gazette.

[41] Bourbonniere M, Feng Z, Intrator O, Angelelli J, Mor V, Zinn JS (2006). The use of contract licensed nursing staff in U.S. nursing homes. Med Care Res Rev.

[42] Dunton N, Gajewski B, Klaus S, Pierson B (2007). The relationship of nursing workforce characteristics to patient outcomes. Online J Issues Nurs.

[43] Roseman C, Booker JM (1995). Workload and environmental factors in hospital medication errors. Nurs Res.

[44] Alonso-Echanove J, Edwards JR, Richards MJ, Brennan P, Venezia RA, Keen J (2003). Effect of nurse staffing and antimicrobial-impregnated central venous catheters on the risk for bloodstream infections in intensive care units. Infect Control Hosp Epidemiol.

[45] Hurst K, Smith A (2011). Temporary nursing staff – cost and quality issues. J Adv Nurs.

[46] Page A (2004). Keeping patients safe: transforming the work environment of nurses.

[47] Republic of South Africa (2005). National Health Act No. 61 of 2003.

[48] DOH (2013). Strategic Plan for Nursing Education, Training and Practice 2012/13-2016/17.

[49] Royal College of Nursing (2004). Agency nursing-RCN guidance for nursing and care agencies.

[50] Royal College of Nursing (2007). Agency nurse induction guidelines- RCN guidance for employers, nursing agencies and nursing staff.

[51] Van Rensburg HCJ (2012). Health and health care in South Africa.

# Sub article: The indirect costs of agency nurses in South Africa: a case study in two public sector hospitals

**DOI:** 10.3402/gha.v8.26494

**Published:** 2015-05-11

**Authors:** Laetitia C. Rispel, Julia Moorman

**Affiliations:** 1 Centre for Health Policy & Medical Research Council Health Policy Research Group, School of Public Health, Faculty of Health Sciences, University of the Witwatersrand, Johannesburg, South Africa; 2 Department of Community Health, School of Public Health, Faculty of Health Sciences, University of the Witwatersrand, Johannesburg, South Africa

**Keywords:** nursing agency, indirect costs, health workforce, South Africa

## Abstract

**Background:**

Globally, flexible work arrangements – through the use of temporary nursing staff – are an important strategy for dealing with nursing shortages in hospitals.

**Objective:**

The objective of the study was to determine the direct and indirect costs of agency nurses, as well as the advantages and the problems associated with agency nurse utilisation in two public sector hospitals in South Africa.

**Methods:**

Following ethical approval, two South African public sector hospitals were selected purposively. Direct costs were determined through an analysis of hospital expenditure information for a 5-year period from 2005 until 2010, obtained from the national transversal Basic Accounting System database. At each hospital, semi-structured interviews were conducted with the chief executive officer, executive nursing services manager, the maternity or critical care unit nursing manager, the human resource manager, and the finance manager. Indirect costs measured were the time spent on pre-employment checks, and nurse recruitment, orientation, and supervision. All expenditure is expressed in South African Rands (R: 1 USD=R7, 2010 prices).

**Results:**

In the 2009/10 financial year, Hospital 1 spent R38.86 million (US$5.55 million) on nursing agencies, whereas Hospital 2 spent R10.40 million (US$1.49 million). The total estimated time spent per week on indirect cost activities at Hospital 1 was 51.5 hours, and 60 hours at Hospital 2. The estimated monetary value of this time at Hospital 1 was R962,267 (US$137,467) and at Hospital 2 the value was R300,121 (US$42,874), thus exceeding the weekly direct costs of nursing agencies. Agency nurses assisted the selected hospitals in dealing with problems of nurse recruitment, absenteeism, shortages, and skills gaps in specialised clinical areas. The problems experienced with agency nurses included their perceived lack of commitment, unreliability, and providing sub-optimal quality of patient care.

**Conclusion:**

Hospital managers and policy-makers need to address the effective utilisation of agency nurses and quality of patient care in tandem.

Against a backdrop of worldwide shortages of health personnel, the World Health Organization (WHO) has published an action framework to enhance the capacity of nurses and midwives to contribute to global health targets, people-centred care, universal health coverage, and health workforce policies (1). This is in recognition of the critical role of nurses as front-line service providers in improving health outcomes and providing quality, cost-effective health care (1, p. 2). Building on the global WHO framework, the guidelines of the Africa region emphasise the importance of the nursing profession in addressing the complex disease burden within countries in the region, attaining the health-related Millennium Development Goals, and improving the performance of weak health systems (2). The regional strategies include, *inter alia*, effective management of nurses and midwives, and research and evidence to aid decision-making (2).

Globally, flexible work arrangements, through the use of temporary nursing staff or the working practices of permanent staff, have emerged as an important strategy for dealing with nursing shortages, particularly in hospitals (3–15). Temporary nursing staff include those supplied through nursing agencies or nursing banks, whereas flexible work practices include part-time work or flexi-time (12). In high-income countries, agency nurses play an important role in assisting hospitals to cope with the variability in the patient demand, maintain patient care, and deal with the problems of recruitment and chronic staff shortages (8, 11, 12). However, several authors have highlighted problems associated with the use of agency nurses (12, 15–20). These include tension among hospital managers, permanent hospital staff, and the agency nurses; perceptions that agency nurses provide lower quality of nursing care with loss of care continuity; and agency nurses having lower commitment and less loyalty, as well as varying levels of competence, dependability, productivity, and ethical practice (12, 15–20).

Although the guidelines of the Africa region on strengthening nursing and midwifery services emphasise the importance of expanding the discourse beyond nurse shortages, and examining expenditure, among other issues (2), there is a dearth of literature on agency nursing in general, and on cost information in particular. In South Africa, there is an emerging research focus on agency nursing as one element of analysing the broader process of casualisation in nursing (21–23). A study on the utilisation and costs of nursing agencies reported elsewhere in this volume found wide variations in the utilisation and management of nursing agencies across the nine provinces (21). The study found that 1.49 billion South African Rands (R) (US$212.64 million) were spent on nursing agencies in the public health sector for the 2009/10 fiscal year (21).

However, there are also indirect costs associated with agency nursing, defined as an ‘expense (e.g. supervision or staff orientation) incurred in joint usage and, therefore, difficult to assign to or identify with a specific cost centre, function, or programme’ (in this case nursing agencies) (24). Several authors have highlighted gaps in the literature regarding cost information on nurse staffing patterns and the use of agency personnel (25–28). The United Kingdom Audit Commission attempted to quantify these indirect costs by studying the average amount of time spent by senior nursing staff arranging temporary cover and found wide variations depending on the existence of a centrally coordinated nursing bank (12).

In light of the considerable amount of money spent on nursing agencies in the South African public health sector (21), the objective of the study was to determine the direct and indirect costs of agency nurses, as well as the advantages and the problems associated with agency nurse utilisation in two public sector hospitals in South Africa.

## Methods

Two public sector hospitals – one central hospital providing tertiary services and one large regional hospital – were selected purposively in Gauteng Province, South Africa.^1^ These two hospitals were part of a larger study on casualisation, agency nursing, and moonlighting conducted in four South African provinces (22). The main reasons for the purposive selection of the two hospitals were: budgetary and staff constraints; the fact that we had obtained detailed expenditure information from the South African National Treasury for the entire public sector and this component was a complementary research activity; the concentration of agency nurses in certain hospitals in the public sector; and the logistical difficulties of collecting in-depth qualitative information on indirect costs from numerous hospitals.

The University of the Witwatersrand Human Research Ethics Committee (Medical) granted ethical approval for the study. The public healthcare authorities and the chief executive officers (CEOs) at the two hospitals also provided study approval. All participants received a study information sheet and were required to sign an informed consent form to indicate their willingness to participate in the study.

This was a financial costing study conducted in 2011. There were two components to data collection: analysis of expenditure information at the two hospitals for a 5-year period from 2005 until 2010 to determine direct costs; and in-depth interviews with executive and middle managers at each of the hospitals to determine indirect costs.

### Determining direct costs

A trend analysis of nursing agency expenditure was conducted at the two selected hospitals. We analysed provincial health expenditure data for the 5-year period from 2005 until 2010, obtained from the national transversal Basic Accounting System database (21). The reasons for the trend analysis were the availability of the financial information as part of the larger study (21) and because it enhanced our understanding of the fluctuations in the utilisation of agency nurses at the selected hospitals over the study period.

The financial data were exported into Microsoft Excel in order to facilitate uniform coding of the expenditure information and to do trend analysis across the entire period (2005/6 through 2009/10). Each financial year was entered into a separate worksheet. The total number of expenditure line items ranged from 30,595 in the 2005/6 financial year to 34,978 in the 2009/10 financial year, totalling 166,466 line items for coding over the 5-year period (21).

Each line item was coded meticulously, depending on the type of expenditure, and similar items were grouped and aggregated. The coding also took into account the healthcare facility where the expenditure occurred. Once each item was coded, cross-tabulations and calculations were done to ensure that the same total expenditure was obtained for each hospital, thus ensuring both validity and reliability of the study results (21).

Overall hospital expenditure consists of all expenditure items, including compensation of employees, goods and services (operational expenditure), transfer payments (to municipalities or non-governmental organisations), and capital expenditure (21). Personnel expenditure, also called ‘compensation of employees’ consists of salaries, staff benefits, and overtime payments. Nursing agency expenditure consists of all expenses paid to commercial nursing agencies (including administration fees) and is paid from the ‘goods and services’ budget of each hospital (21).

For each hospital, the overall hospital, personnel and nursing agency expenditure was calculated for each financial year. Agency nursing expenditure as a proportion of the total hospital expenditure and of the personnel expenditure was then calculated, for each hospital and for each financial year (21). All expenditure is expressed in South African Rands (R: 1 USD=R7, 2,010 prices).

### Determining indirect costs

In the study, an agency nurse was defined as a nurse registered with the South African Nursing Council (SANC) who is employed by a commercial nursing agency, providing temporary cover in a hospital. The nurse is paid by the agency, which, in turn, charges the hospital a fee. Agency nurses may be registered with several agencies as well as having a job in a public or private healthcare facility (19).

During 2011, semi-structured interviews were conducted with the following individuals in each hospital: the CEO (1), the executive nursing services manager (1), the nursing manager of the maternity or critical care unit (1), the human resource manager (1), and the finance manager (1). Ten people were interviewed at the two hospitals.

Following informed consent, the interview schedule, which was pilot-tested at another hospital, collected the following information: background characteristics, including any outstanding features or peculiarities; the number of nursing agencies on contract; the utilisation of nursing agencies for the provision of temporary nursing staff; the temporary nursing cover arrangements used in the hospital (e.g. part-time staff, overtime); the indirect costs of using agency nurses; the advantages of agency nursing; and the challenges experienced with the use of temporary nursing staff within the hospital.

In this study, the indirect costs measured were the time taken or spent on: pre-employment checks (e.g. checking SANC registration, identity, or qualifications); identifying the need for agency nurses; recruitment; orientation of the nurse; supervision required, including dealing with any problems related to the agency nurses (e.g. patient or doctor complaint); and the administration related to the agencies, which includes checking, processing, and paying invoices. The time taken was expressed in hours per week and respondents were only asked about their own time spent on each of these activities. One working week was assumed to be equal to 40 hours, in line with the requirements of South Africa's Basic Conditions of Employment Act (29). This time value was then translated into a monetary figure, based on the 2010 expenditure on nursing agencies at the two case study hospitals. The qualitative information from the interviews was analysed using thematic content analysis (30).

## Results

### Background information

Table 1 shows background information on the two case study hospitals. At the time of the study in the second half of 2011, Hospital 2 (a large regional hospital) had stopped using agency nurses due to cash flow problems and because of a directive from the Provincial Department of Health to discontinue the use of agency staff. Nonetheless, both hospitals had utilised agency nurses in the 2009/10 financial year on a daily basis, particularly in the maternity and critical care units. The most frequently used categories of agency nurses were professional nurses (with 4 years of training) and enrolled nurses (with 2 years of training).

**Table 1:** Background characteristics of case study hospitals

	Hospital 1	Hospital 2
Characteristics	Large public sector, tertiary hospital rendering specialised and highly specialised services upon referral from other hospitals and/or clinics. One of 10 central hospitals in South Africa, part of an academic health complex, and a teaching hospital for one of the country's eight medical schools. Located in a city, close to the central business district.	Large public sector, regional hospital rendering specialised, secondary-level services. One of the regional teaching hospitals for one of the country's eight medical schools. Located in a densely populated, socio-economically disadvantaged residential area (known as a township), characterised by a large number of foreigners and informal settlements.
Number of beds	832	840
Managers interviewed	CEO, nursing service manager, maternity unit manager, finance and human resource managers	CEO, nursing service manager, critical care unit manager, finance and human resource managers
Utilisation of agency nurses	Yes	Yes^a^
Types of agency nurses utilised	Professional nurses only (4 years training)	Professional and enrolled nurses (2 years training)
Clinical areas that utilise agency nurses	Maternity and critical care units	Throughout hospital
Reported frequency of use	Every day	Every day

a At the time of the research, Hospital 2 had stopped using agency nurses because of cash flow problems.

### Trends in nursing agency expenditure

Figure 1 shows the trends in nursing agency expenditure for the two case study hospitals for the 5-year period from 2005 to 2010. As shown in Fig. 1, at Hospital 1, agency expenditure was R21.91 million (US$3.1 million) in the 2005/6 financial year, peaking at R38.86 million (US$5.55 million) in 2009/10. The graph for Hospital 1 shows a sharp increase between 2005/6 and 2006/7, and then a fairly consistent pattern of agency nurse expenditure for the remainder of the study period. In contrast, there was an erratic expenditure pattern in Hospital 2, ranging from R120,696 (US$17,242) in 2005/6 to a peak of R10.40 million (US$1.49 million) in the 2009/10 financial year.

**Fig. 1:** Trends in agency nurse expenditure, 2005/10. Source: South African National Treasury, National Basic Accounting System.

Table 2 shows agency expenditure as a proportion of the overall hospital expenditure and as a proportion of personnel expenditure (compensation of employees) for the 2009/10 financial year.

**Table 2:** Expenditure on agency nurses 2009/10

	Hospital 1 South African Rand (R)	Hospital 2 South African Rand (R)
Overall hospital expenditure	1,143,417,253	396,493,291
Compensation of employees (personnel)	727,229,805	251,458,449
Annual expenditure on agency nurses	38,864,376	10,404,186
Weekly expenditure on agency nurses	747,392	200,081
Expenditure on agency nurses as % of overall hospital expenditure	3.40%	2.62%
Expenditure on agency nurses as % of compensation of employees (personnel)	5.34%	4.14%

Source: South African National Treasury, National Basic Accounting System.

### Indirect costs of agency nurses

Table 3 shows the estimated weekly time spent on recruitment and management of agency nurses.

**Table 3:** Estimated weekly time spent on recruitment and management of agency nurses^a^

Indirect cost element	Hospital 1	Hospital 2
Pre-employment checks	Not done	Not done
Identify need for agency nurses	1	4
Arranging cover/recruitment of agency nurses	10.5	20
Orientation	2	8
Supervision, including dealing or managing problems	28	20
Verification of invoices	0	4
Processing of invoices	10^b^	4
Processing of payments	Done centrally	Done centrally
Monitoring of expenditure	Not done	Not done
Total hours per week	51.5	60
Estimated indirect cost per week	962,267	300,121

a In calculating hours per week, it was assumed that managers worked 8 hours per day and 5 days per week.

b Hospital 1 spent 4 hours per agency per invoice every 2 weeks. There were five agencies, averaging 10 hours per week.

As shown in Table 3, the case study hospitals did not do any pre-employment checks such as registration with the SANC, or confirmation of the nurse's identity and/or qualifications. The burden of arranging the hiring of agency nurses was considerable and a time-consuming process for nursing managers, with Hospital 1 spending 10.5 hours per week on these activities, and Hospital 2 spending 20 hours per week. The time taken to find nurses was compounded by the agencies themselves being ‘short-staffed’, nurses preferring to work in the private health sector as the workload was lighter, and the public sector's delays in paying nursing agencies. Managers indicated that they spent relatively little time per week on induction or orientation of agency nurses, because it was not a worthwhile undertaking if the nurse only worked one shift and never returned to the hospital.

The nursing managers interviewed indicated that agency nurses needed considerably more supervision than did permanent staff. Agency nurses were not allowed to administer medication unless supervised. They also required supervision with report-writing and patient care documentation, wound dressings, and the use of highly specialised equipment in critical care units. As Hospital 1 renders highly specialised tertiary services, they also provided additional supervision for agency nurses taking care of women with high-risk pregnancies.

Hospital 1 nursing managers indicated that they did not spend time on the verification of invoices, whereas Hospital 2 estimated that 4 hours per week were spent on this activity.

At both hospitals, the finance managers were responsible for processing invoices. The actual payments were done centrally at the provincial shared services centre, which existed at the time of the study. The finance manager at Hospital 1 indicated that 4 hours were spent per agency to process invoices, there were five agencies on contract, and these agencies sent invoices every 2 weeks. This amounted to a total of 40 hours per month, averaging 10 hours per week. The finance manager at Hospital 2 indicated that half a day or 4 hours per week were spent on processing invoices.

Hence, the total estimated time spent per week on indirect cost activities at Hospital 1 was 51.5 hours, and 60 hours at Hospital 2. The estimated monetary value of this time at Hospital 1 was R962,267 ($137,467), and at Hospital 2 the value was R300,121 ($42,874) (Table 3). At both hospitals, the indirect costs per week exceeded the direct costs of nursing agencies.

### Perceived advantages of agency nurses

Nursing agencies assisted hospitals in dealing with problems of nurse recruitment, shortages, and skills gaps, particularly in specialised areas such as critical care units. The CEO at Hospital 1 was also of the opinion that there is some transfer of risk to the agency, in that they are obliged to provide nurses to hospitals upon request. It was also felt that agency nursing enables nurses with young children to work in a flexible manner and that it enables public hospital nurses who do agency nursing in the private sector to gain skills such as inventory control.

### Perceived disadvantages of agency nurses

The CEOs and nursing managers at both hospitals listed several negative experiences with and disadvantages of utilising agency nurses. Three themes emerged from the responses: human resource issues, quality of care, and cost. These themes overlap and are elaborated below.

Human resource issues tended to dominate the negative experiences or disadvantages. These included: poor attitudes of agency nurses, their perceived lack of commitment, disloyalty, unreliability, reluctance to take on ‘extra duties’ or perform certain nursing tasks, time taken on supervision, poor relationship with doctors, and perceptions that they do not have the same ‘culture of caring’ compared to permanent staff. The qualitative comments provide insights into hospital managers’ views, whereas Table 4 shows the nursing managers’ subjective experience of the performance of agency nurses in the 3 months preceding the study.

The extra demands on supervision are huge. Most patients are high-risk, and they [agency nurses] need extra supervision when they take care of high-risk patients with eclampsia, induction of labour, and so on. (Nursing manager, Hospital 1)

Agency nurses need additional supervision, especially for procedures such as assisting with the insertion of a central line. They are not allowed to do everything – we always have to co-check medication, and they do limited administration. They often have limited experience with technology. (Maternity unit nursing manager, Hospital 2)

**Table 4:** Nursing managers’ experience of the performance of agency nurses in preceding 3 months (*n*=4)

Indicator	Every day	Several times per week	Once per week	Once per month	Never
Late arrival		3			1
Not turning up (no show)	1	1	2		
Turning away the nurse					4
Inappropriate experience		1		2	1
Too tired to work			1		3
Not familiar with medical equipment		3		1	
Leaving early				1	3
Doctor unhappy	1			2	1

Those interviewed were of the opinion that the quality of care provided by agency nurses leaves much to be desired.

There are more serious adverse events with agency nurses – they are bad-tempered with patients. They are tired and sleep all the time. (CEO, Hospital 2)

They do not have the right skills … [patient care] policies change and they need updates. They do not take responsibility or accountability, and these [performance] gaps are much greater at night. I think there are definitely more adverse events. (Maternity unit manager, Hospital 2)

The CEO at Hospital 1 was of the opinion that the risks of using agency nurses outweighed the benefits and that the cost was much higher than would be incurred if they had permanent staff. Furthermore, the hospital overspent its allocated budget consistently, and nursing agencies were seen as a contributing factor. The CEO of Hospital 2 indicated that their overall experience with nursing agencies was poor, with little value for money, and noted that they had stopped all agency nurses during 2010, a decision accelerated because of hospital cash flow problems.

## Discussion

This case study of the indirect costs of two large public sector hospitals found that there was extensive utilisation of agency nurses in these hospitals in the critical care and maternity units. These agency nurses assisted the two hospitals in dealing with the difficulties of nurse recruitment and shortages. Hospital 1, a large teaching hospital providing tertiary care services, spent R38.86 million (US$5.55 million) on agency nurses in the 2009/10 financial year, or 5.34% of the personnel budget. Hospital 2 spent R10.40 million (US$1.49 million) in the same period, or 4.14% of the personnel budget.

At face value, it appears that this was a reasonable proportion of the personnel budget spent on agency nurses, comparing well to the UK National Health Service trusts where the average agency expenditure across all NHS trusts was 3.85% of personnel expenditure in the 2007/8 financial year (12). However, the agency expenditure as a proportion of the personnel budget was higher than the Gauteng provincial average of 2.28% (21). Furthermore, the case study found that the estimated weekly indirect costs of agency nurses exceeded the direct costs by 29% at Hospital 1, and by 50% at Hospital 2. The recruitment and supervision of agency nurses accounted for the major part of the indirect costs. In the UK, the National Audit Office (NAO) estimated that the average amount of time spent by senior staff in arranging temporary cover ranged from 9–30 minutes per day (15), which is more time than that spent at the two case study hospitals.

Nursing managers indicated that the time taken to recruit nurses was compounded by the inability of the nursing agencies to supply them. This was despite the fact that Hospital 1 had contracts with five separate nursing agencies. Other factors, such as the preference of nurses to work in private hospitals rather than the public sector, delays in payment, and the location of Hospital 2 in a socio-economically disadvantaged area, also hindered the recruitment of agency nurses.

In both hospitals, managers indicated that a considerable amount of time was spent on supervision of agency nurses. There was a general perception that the performance of agency nurses was sub-optimal, compared to permanent hospital staff. At the same time, nursing managers indicated that little time was spent on induction, as it was not seen as a worthwhile activity for temporary staff, who may never return to the hospitals. The UK NAO also found that the average induction time for agency nurses was low, as senior managers tended to weigh up the benefits of the induction versus the costs in terms of time (15). However, any nurse, despite qualifications or competencies, is less likely to perform well in an unfamiliar setting, thus some orientation or induction is critical in order to reduce the potential risk to patient care. Furthermore, agency nurses’ performance may be sub-optimal because they are sent to understaffed areas.

The study found that the two hospitals did not do pre-employment checks, including nursing council registration, nurse identity, and nurse qualifications. This could jeopardise patient safety, thus exacerbating the negative impact on the quality of care. The UK NAO has cautioned about the potential risks of deception and has suggested that hospitals should ensure that the nurses are competent to perform the tasks asked of them, because incompetence could add to the workload of permanent staff, and jeopardise patient care and safety (15). Hence it is important that hospitals invest in these quality control mechanisms.

The qualitative comments revealed managers’ perceptions of the poor attitudes of agency nurses, the lack of a caring attitude, their perceived lack of commitment, unreliability, and their reluctance to perform certain nursing tasks. Although this was a two-hospital case study, these findings are similar to those highlighted by other authors (12, 15–20). Table 4 shows the problems experienced by nursing managers in the 3 months preceding the study, which ranged from late arrival and agency nurse exhaustion, to lack of familiarity with medical equipment.

Although they could not produce evidence, there was a general perception among managers in the two hospitals that agency nurses provide sub-optimal quality of care. These perceptions might be justified, as studies in the US and UK have found that casual or temporary staffing contributes to poor quality of patient care (31–34).

There are a number of study limitations. The study was conducted in two public sector hospitals and cannot be generalised to other hospitals. This was a financial costing study, rather than a full economic evaluation which would require the externality costs of various forms and quantifiable indirect costs, such as administration. In the analysis of direct costs of agency nurses, we assumed that these were equal to expenditure. Although financial statistics tend to be more accurate than other information, the study used official government statistics that the provincial health department submitted to the National Treasury (21). The study relied on self-reported information from hospital managers. They estimated the time spent on activities used to measure indirect costs, which could have resulted in over- or underestimates. Future studies would need to quantify the exact time spent on indirect activities. We also did not quantify all indirect costs, such as administrative costs (telephone, consumables), and the costs to the hospitals or to patients of poor quality of care, including adverse events. Furthermore, we assumed that the value of time spent by a hospital finance officer on paying billswas the same as that spent by a senior nursing manager in recruiting agency nurses.

Notwithstanding these limitations, this case study has numerous strengths. The focus on indirect costs of agency nurses is unique and gives managers an idea of the risks that need to be managed. Another interesting feature of the case study is the detailed analysis of nursing agency expenditure at the two hospitals, and the trends in such expenditure over a 5-year period. The study adds to the empirical evidence and knowledge base on the widespread use of temporary agency nurses in South African public sector hospitals. The study also illustrates the usefulness of a trend analysis of routine financial data at hospital level, combined with qualitative insights on indirect costs, in informing hospital management approaches.

The study findings have implications for quality of care and human resource management. The findings suggest that the quality of patient care may be compromised by utilisation of agency nurses. However, the impact of the high use of agency nurses on the quality of patient care was not measured, and this is an area for further research. It is of concern that there is no risk management and no formal system to monitor the quality of care provided by agency nurses at the two case study hospitals. It is therefore recommended that both hospitals put in place systems to monitor the performance of agency nurses. This should include: giving feedback to the agency on the performance of nurses, and improving the quality of pre-employment checks, such as nursing council registration, identity checks, verification of qualifications, and previous work experience. In light of the direct and indirect costs associated with the use of agency nurses, in particular the time taken by managers to arrange temporary cover, it is important that they are used effectively. For this reason, agency nurses need appropriate orientation to the ward and hospital.

The utilisation of agency nurses is influenced by various factors, including staff shortages, changing patient disease profiles, poor staff planning, staff absenteeism, and lack of involvement of nurses in decision-making (8, 11, 19, 35). In order to address these issues, hospital nursing managers should find creative alternatives to agency nurse utilisation. As a minimum, the following are needed at hospital and ward levels: an analysis and understanding of the reasons why agency nurses are being booked, introduction of greater flexibility for permanent staff, procedures to manage nursing vacancies, analysis of nurse absenteeism patterns, and mechanisms to reduce unacceptable levels of absenteeism (12, 15).

## Conclusion

The delivery of safe and effective patient care in hospitals is dependent on the availability of competent and motivated nurses (36, 37). This study has highlighted the considerable direct and indirect costs of using agency nurses in the two public sector hospitals. In general, agency nurses enable numerical flexibility in hospitals and is an approach to the management of staff shortages and nurse absenteeism (12). At the same time, the perceived disadvantages of agency nurses include high cost, the provision of sub-optimal patient care, and attitude and performance problems. Hitherto, the management of temporary agency nurses has not featured on the agenda of executive hospital managers or health policy-makers. In light of the government's major emphasis on quality of care in South Africa, the interrelated issues of the effective utilisation of agency nurses and quality of care would need to be addressed in tandem.

## Footnotes

1 A private hospital was also selected, but is excluded from the analysis because of lack of the availability of trend information.

## References

[1] WHO (2010). Strategic directions for strengthening nursing and midwifery services 2011–2015.

[2] WHO AFRO (2007). WHO guidelines for implementing strategic directions for strengthening nursing and midwifery services in the African region 2007–2017.

[3] Adams B (1989). Agency nursing: the hospital response. J Nurs Adm.

[4] Aitken RL, Manias E, Peerson A, Parker JM, Wong K (2002). Agency nursing in metropolitan Melbourne: final research report.

[5] Baumann A, Kolotylo C (2010). Forecasting future workforce demand: a process evaluation.

[6] Buchan J, Thomas S (1995). Managing temporary nursing staff: nurse banks in Scotland. Health Manpow Manage.

[7] Creegan R, Duffield C, Forrester K (2003). Casualisation of the nursing workforce in Australia: driving forces and implications. Aust Health Rev.

[8] Hurst K, Smith A (2011). Temporary nursing staff – cost and quality issues. J Adv Nurs.

[9] Manias E, Aitken R, Peerson A, Parker J, Wong K (2003). Agency-nursing work: perceptions and experiences of agency nurses. Int J Nurs Stud.

[10] Manias E, Aitken R, Peerson A, Parker J, Wong K (2003). Agency nursing work in acute care settings: perceptions of hospital nursing managers and agency nurse providers. J Clin Nurs.

[11] Massey L, Esain A, Wallis M (2009). Managing the complexity of nurse shortages: a case study of bank and agency staffing in an acute care Trust in Wales, UK. Int J Nurs Stud.

[12] Mercer M, Buchan J, Chubb C (2010). Flexible nursing. Report for NHS Professionals.

[13] North N, Hughes F, Rasmussen E, Finlayson M, Ashton T, Campbell T (2006). Use of temporary nurse mechanisms by New Zealand's District Health Boards. N Z J Employ Relat.

[14] Zeytinoglu IU, Denton M, Davies S, Baumann A, Blythe J, Boos L (2006). Retaining nurses in their employing hospitals and in the profession: effects of job preference, unpaid overtime, importance of earnings and stress. Health Pol.

[15] National Audit Office (2006). Improving the use of temporary nursing staff in NHS acute and foundation trusts.

[16] Braddy PK, Washburn TA, Caroll LL (1991). Factors influencing nurses to work for agencies. West J Nurs Res.

[17] Hughes KK, Marcantonio RJ (1991). Recruitment, retention and compensation of agency and hospital nurses. J Nurs Adm.

[18] Jackson BS (1989). Agency nursing: costs and quality [Opinion]. J Nurs Adm.

[19] O'Reilly J, Trueman P, Ganderton M, Wright D (2006). Results of census of temporary nursing staff in acute hospital and foundation trusts: final report.

[20] Peerson A, Aitken RL, Manias E, Parker JM, Wong K (2002). Agency nursing in Melbourne, Australia: a telephone survey of hospital and agency managers. J Adv Nurs.

[21] Rispel LC, Angelides G (2014). Utilisation and costs of nursing agencies in the South African public health sector, 2005–2010. Glob Health Action.

[22] Rispel LC, Blaauw D, Chirwa T, de Wet K (2014). Factors influencing agency nursing and moonlighting among nurses in South Africa. Glob Health Action.

[23] Rispel LC, Chirwa T, Blaauw D (2014). Does moonlighting influence South African nurses’ intention to leave their primary jobs?. Glob Health Action.

[24] Business dictionary Definition of indirect costs. http://wwwbusinessdictionarycom/definition/indirect-costhtml.

[25] Duffield C, Roche M, O'Brien-Pallas L, Catling-Paull C (2009). Implications of staff ‘churn’ for nurse managers, staff and patients. Nurs Econ.

[26] Goryakin Y, Griffiths P, Maben J (2011). Economic evaluation of nurse staffing and nurse substitution in health care: a scoping review. Int J Nurs Stud.

[27] Brooten D, Youngblut JM, Kutcher J, Bobo C (2004). Quality and the nursing workforce: APNs, patient outcomes and health care costs. Nurs Outlook.

[28] Spetz J (2005). The cost and cost-effectiveness of nursing services in health care. Nurs Outlook.

[29] Republic of South Africa (2013). Basic Conditions of Employment Act (as amended).

[30] Miles M, Huberman AM, Saldaña J (2014). Qualitative data analysis: a methods sourcebook.

[31] Bourbonniere M, Feng Z, Intrator O, Angelelli J, Mor V, Zinn JS (2006). The use of contract licensed nursing staff in U.S. nursing homes. Med Care Res Rev.

[32] Dunton N, Gajewski B, Klaus S, Pierson B (2007). The relationship of nursing workforce characteristics to patient outcomes. Online J Issues Nurs.

[33] Roseman C, Booker JM (1995). Workload and environmental factors in hospital medication errors. Nurs Res.

[34] Alonso-Echanove J, Edwards JR, Richards MJ, Brennan P, Venezia RA, Keen J (2003). Effect of nurse staffing and antimicrobial-impregnated central venous catheters on the risk for bloodstream infections in intensive care units. Infect Control Hosp Epidemiol.

[35] Page A (2004). Keeping patients safe: transforming the work environment of nurses.

[36] South African National Department of Health (2010). National core standards for health establishments in South Africa.

[37] Global Health Workforce Alliance, WHO (2013). A universal truth: no health without a workforce.

# Sub article: Exploring the characteristics of nursing agencies in South Africa

**DOI:** 10.3402/gha.v8.27878

**Published:** 2015-05-11

**Authors:** Omolola I. Olojede, Laetitia C. Rispel

**Affiliations:** Centre for Health Policy & Medical Research Council Health Policy Research Group, School of Public Health, Faculty of Health Sciences, University of the Witwatersrand, Johannesburg, South Africa

**Keywords:** nursing agency, nurses, labour broker, health workforce, South Africa

## Abstract

**Background:**

Nursing agencies are temporary employment service providers or labour brokers that supply nurses to health establishments.

**Objective:**

This study was conducted to determine the characteristics of nursing agencies and their relationship with clients in the health sector.

**Methods:**

During 2011, a cross-sectional national survey of 106 nursing agencies was conducted. After obtaining informed consent, telephone interviews were conducted with a representative of the selected nursing agency using a pretested structured questionnaire. Questions focused on the following: ownership, date of establishment, province of operation, distribution of clients across private and public health facilities; existence of a code of conduct; nature of the contractual relationship between nursing agencies and their clients, and numbers and cadres of nurses contracted. The survey data were analysed using STATA^®^ 12.

**Results:**

Fifty-two nursing agencies participated in the survey, representing a 49% response rate. The study found that 32 nursing agencies (62%) served private-sector clients only, which included private hospitals, homes for elderly people, patients in private homes, and private industry/company clinics, and only four (8%) of the agencies served the public sector only. Twenty-seven percent of nursing agencies provided services to homes for elderly individuals. Nursing agencies were more likely to have contracts with private-sector clients (84%) than with public-sector clients (16%) (*p* = 0.04). Although 98% of nursing agencies reported that they had a code of conduct, the proportion was higher for private-sector clients (73%) compared to public-sector clients (27%). In terms of quality checks and monitoring, 81% of agencies agreed with a statement that they checked the nursing council registration of nurses, 82% agreed with a statement that they requested certified copies of a nurse's qualifications. Only 21% indicated that they conducted reference checks of nurses with their past employers.

**Conclusions:**

Nursing agencies should enhance their quality assurance mechanisms when engaging contracted staff. Overall, the study findings suggest the need for improved governance and management of nursing agencies in South Africa.

The health workforce crisis remains a priority in all countries around the world (1), but is particularly acute in Africa. In South Africa, addressing health workforce challenges is critical in order to achieve health development goals (2). Nurses in South Africa, as elsewhere, make up the largest single group of health-care providers, and their role in attaining quality health care services cannot be over-emphasised (3, 4). They play various roles in the health sector, and they are often the link between communities or patients and health-care facilities (5). However, there are numerous challenges faced by this group of health-care providers. These include, *inter alia*, changes in disease patterns; growing demand for health-care services; an ageing nursing workforce; a shortage of nurses; and an increasing process of casualisation of nursing work, evidenced by practices such as moonlighting (having a second job additional to a primary job) and agency nursing (3, 6–8).

Casualisation, or the employment of workers on a contract part-time basis without the benefits associated with permanent employment, is a global phenomenon (9). Casual employment is done typically through a temporary employment service (TES) agency or labour broker, that employs the casual worker and then contracts the person to a company, organisation, or individual that needs the service (10). Casualisation is a triangular form of employment that includes a third party who is an intermediary between the employee and the employer. Thus there is no formal relationship between the employee and employer, and labour laws do not always protect the employees (10). Figure 1 illustrates the relationship between the casual worker, the TES agency (the labour broker), and the employer.

**Fig. 1:** Triangular employment in labour brokering.

This method of employment has also found its way to the health sector, influenced by globalisation of the health workforce, individual preferences for flexibility, and the additional income it offers (11). Developments in the health sector are particularly evident in the employment of nurses through nursing agencies, which play the role of TES agencies or labour brokers (12, 13).

In South Africa, anecdotal evidence suggests that there has been a growth of the nursing agency industry in the past decade. The 1978 Nursing Act (Section 1) defines a nursing agency as ‘a business which supplies registered nurses or midwives or enrolled nurses or nursing auxiliaries to any person, organisation or institution, whether for gain or not and whether in conjunction with any other service rendered by such business or not’ (14).

Much of the literature on nursing agencies comes from Australia, Canada, the United Kingdom, and the United States (11, 15–24). This literature is of limited use in this study, because it tends to focus on agency nurses, rather than on the nursing agency industry. The focus of the literature is on nurses’ motivation for agency employment, management of agency nurses by the hospital administration, the quality of health delivery by agency nurses, and permanent nurses’ relationship with agency nurses (11, 15–24). In South Africa, research has shown that there is widespread utilisation of nursing agencies in the public health sector (6). In the 2009–2010 financial year alone, 1.49 billion South African rands (US$212.64 million) were spent on nursing agencies in this sector (6). There is also extensive utilisation of nursing agencies in the private health sector (25). Notwithstanding the health-care expenditure on nursing agencies, nurses moonlight through commercial nursing agencies. A 2010 cross-sectional survey found that 37.8% of study participants engaged in agency nursing in the year preceding the survey (7).

Little is known about nursing agencies except that they play the role of labour brokers in the health sector. At the time of the study, there were heated debates in South Africa on the future of labour brokers and on the negative impact of nursing agencies on the public health sector (26–28). In light of limited empirical information and a proposed ban on labour brokers in South Africa, this study was conducted to determine the characteristics of nursing agencies and their relationship with clients in the health sector and to explore possible health policy implications of the findings.

## Methods

During 2011, a cross-sectional national survey of nursing agencies was conducted. Ethical clearance for the study was obtained from the University of the Witwatersrand's Human Research Ethics Committee (Medical). Standard ethical procedures were adhered to. These included a detailed information sheet, informed consent, and voluntary participation.

The sampling frame consisted of all registered nursing agencies on the 2010 database of the South African Nursing Council (SANC). The latter is a regulatory authority established in terms of the Nursing Act that governs the nursing and midwifery professions in South Africa (14). Historically, commercial nursing agencies were required by law to register with the SANC (14). At the time of the study, the SANC database presented the best available information, as neither the Department of Health nor the Department of Labour had any database or consolidated information on nursing agencies.

Nursing agencies that were members of the Association of Nursing Agencies in South Africa (ANASA), a voluntary umbrella body set up to represent the industry, were excluded because a separate study focused on ANASA members. Agencies that had closed and were no longer in operation were also excluded. In cases where agencies had multiple branches, only one branch was selected to represent the agency.

Previous information indicated that the majority of nursing agencies were located in the urban provinces of Gauteng and the Western Cape. The agencies were grouped into three strata, namely ‘Gauteng’, ‘Western Cape’, and ‘Others’. A stratified random sample of agencies was then selected from each stratum, proportional to the number of agencies in each stratum, totalling a sample of 106 nursing agencies. The details of the sampling procedure are shown in Fig. 2.

**Fig. 2:** Sampling approach and calculation of sample size.

An introductory call was made to each of the selected agencies to explain the purpose of the study and to invite their participation. The information sheets and consent forms were sent by email or fax to the agencies. Once the agency representative had agreed to participate, the questionnaire was completed over the telephone.

The survey questionnaire was pretested and structured to focus on the following: agency ownership, date of establishment, province(s) of operation, distribution of clients across private and public health facilities, the existence of a code of conduct (typically an agreement between the agency and the client that sets out principles of engagement, ethical conduct, and mechanisms of communication), the nature of the contractual relationship between nursing agencies and their clients, and the number and categories of nurses employed. Using a seven-point Likert scale, the advantages and challenges of agencies were elicited using a series of statements to which a representative of the agency was asked to indicate the extent of agreement or disagreement. A final open-ended question elicited any comments or suggestions about nursing agencies or the health system.

To ensure a high response rate, an average of three follow-up calls on three separate days were made during office hours to each agency, but in some instances up to nine calls were made to an agency.

The data were analysed using STATA^®^ 12. The Likert scale options of strongly agree, agree, and slightly agree were pooled into one group labelled ‘agree’, while strongly disagree, disagree and slightly disagree were grouped as ‘disagree’. A descriptive analysis was conducted that included frequency tabulations of characteristics of nursing agencies and further cross-tabulations of each of these characteristics by province to investigate any statistical associations. The Fisher's exact test or the Chi-square test was used to test associations between variables. All tests were done at a 95% confidence interval. The qualitative information from the response to the open-ended question was analysed using thematic content analysis (29).

## Results

There were 52 nursing agencies that participated in the survey, representing a 49% response rate. Of these, 26% of the nursing agencies were located in Gauteng Province, 9% in the Western Cape, and 15% were from the other seven provinces.

There were four categories of the non-respondents (51%) in the study: 18% of the agencies were not operational or were closed, and this aspect only became known during the actual fieldwork; 16% of agencies did not answer the phone, despite a minimum of three phone calls on three separate days during office hours; 2% of agencies were ineligible because they were members of ANASA, and this only came to light during the survey; and 15% of agencies refused to participate in the study.

### Characteristics of nursing agencies

The overall characteristics of responding nursing agencies are shown in Table 1. The majority of agencies (*n* = 40; 77%) were established between 2000 and 2009. Most of the agencies surveyed were not owned by larger organisations (90%); only five agencies had a parent organisation (10%), and 83% of agencies had only one branch. One agency from Gauteng Province had five branches.

**Table 1:** Characteristics of nursing agencies

Characteristics of nursing agencies	Gauteng		Western Cape		Others		Total		
	
Sample size (*n*)/%	*n*=27	%	*n*=9	%	*n*=16	%	*n*=52	%	p*
Mean number of years in business (SD)	8.2 years (SD = 7.5)								
	Clients of nursing agencies [No. (%)]^a^								
Homes for elderly people	8	30	4	44	2	13	14	27	0.197
Provincial Departments of Health	4	15	3	33	3	19	10	19	0.50
Private Hospital Group 1^b^	4	15	2	22	1	6.3	7	14	0.506
Private Hospital Group 2^b^	5	19	0	–	4	25	9	17	0.277
Private Hospital Group 3^b^	4	15	1	11	4	25	9	17	0.621
Other private hospitals	3	11	0	–	4	25	7	14	0.187
Private patients	11	41	6	67	6	38	23	44	0.322
Private-sector clients only^c^	–	–	–	–	–	–	32	62	
Public-sector clients only^d^	–	–	–	–	–	–	4	8	
No public- or private-sector clients	–	–	–	–	–	–	10	19	
Public- and private-sector clients	–	–	–	–	–	–	6	12	
	Year established [No. (%)]								
≤1964	1	4	0	–	0	–	1	2	0.530
1986–1994	1	4	1	11	1	6	3	6	
1995–1999	4	15	3	33	1	6	8	15	
2000–2009	21	78	5	56	14	88	40	77	
	Branches of agencies [No. (%)]								
1 branch	24	89	7	78	12	75	43	83	0.566
2 branches	2	7	1	11	3	19	6	12	
3 branches	0	–	1	11	1	6	2	4	
5 branches	1	4	0	–	0	–	1	2	
	Ownership of agencies [No. (%)]								
Owned by larger organisations	1	4	2	22	2	13	5	10	0.272

a These were not mutually exclusive.

b There are three large private hospital groups in South Africa – numbers are used here for the sake of anonymity.

c Private-sector clients are combined: private hospitals, homes for elderly people, private home patients, and private industry/company clinics.

d Public-sector clients are combined Provincial Departments of Health.

* *P*-value from Chi-square test of association.

At the time of the survey, 27% of nursing agencies had homes for elderly people as clients, followed by the provincial departments of health (19%). At least 10 agencies supplied casual staff to at least one provincial department of health. A total of 23 (44%) nursing agencies served private patients in their homes. There were no statistical significant differences between the characteristics of the nursing agencies and the province in which they were located (Table 1).

Table 1 also shows that 32 nursing agencies (62%) served private clients only, 6 (12%) agencies served both public and private clients, whereas 10 (19%) nursing agencies served neither public-sector nor private-sector clients and had no clients at the time of the survey or were inactive.

### Staff contracted by nursing agencies

In terms of categories of casual staff, 86% of agencies reported that they contracted professional nurses, 65% contracted enrolled staff nurses, whereas 61% and 67% of agencies contracted nursing assistants and caregivers, respectively. The median number of casual staff (nurses or caregivers) registered with agencies was 50 (IQR = 15–120).

### Relationship between nursing agencies and clients

Overall, 77% indicated that they had formal contracts or agreements with their clients. Nursing agencies were more likely to have contracts with private-sector clients (84%) than with public-sector clients (16%), and this difference was statistically significant (*p=*0.04). Although 98% of nursing agencies reported that they had a code of conduct, the proportion was higher for private-sector clients (73%) than for public-sector clients (27%). However, this difference was not statistically significant. Similarly, the majority of agencies (96%) stated that they had a reporting mechanism for client complaints, and this applied to 72% of private-sector clients, as opposed to 28% of public-sector clients. This client reporting mechanism was primarily verbal, rather than in writing (Table 2).

**Table 2:** Nursing agencies’ reported relationships with their clients

Variable	Private-sector clients^a^ (%)	Public-sector clients^b^ (%)	Total (%)	*P*
Formal contracts with clients	31 (84)	6 (16)	37 (77)	0.04*
Policy to guide supply of nurses	30 (73)	11 (27)	41 (82)	0.57
Existence of code of conduct	35 (73)	13 (27)	48 (98)	0.70
Existence of client complaint reporting mechanism	34 (72)	13 (28)	47(96)	0.53

a Private-sector clients are combined: private hospitals, homes for elderly people, private home patients and private industry/company clinics.

b Public-sector clients are combined provincial departments of health.

* Statistically significant at 0.05 level.

Participating agencies reported that the supply of casual staff to clients is mostly based on a client's demand for the type of staff needed (92%), rather than on the individual nurse's preference (35%). Nursing agencies reported that the median number of nursing staff or caregivers allocated on a daily basis to clients was 15 (IQR = 5–31.5).

In terms of clinical services (closely related to the types of clients), 37% of agencies provided nurses for geriatric care, followed by adult intensive care units (35%) and other services (10%). Other services included HIV testing in some private organisations and occupational health services.

In terms of quality checks and monitoring, 81% of agencies agreed with a statement that they checked the SANC registration of nurses, 82% agreed with a statement that they requested certified copies of a nurse's qualifications. Only 21% indicated that they conducted reference checks of nurses with their past employers.

### Challenges experienced by nursing agencies

Table 3 shows the agencies’ responses to a series of statements in the questionnaire on possible challenges experienced by them.

**Table 3:** Reported challenges experienced by nursing agencies

Reason	% Agreement
There is a shortage of specialised nurses.	94
We find it challenging to recruit nurses.	83
Fixed commission rate.	80
The government is supportive of nursing agencies.	67
Hospitals are willing to partner with agency on nursing training.	44
Hospitals pay their fees on time.	44
Nurses are committed and loyal professionals.	29
Client expectations of nursing agencies are clear.	29
The performance of retired nurses is unsatisfactory.	28
It is easy to communicate with the hospitals.	18

Three themes emerged from the responses to the open-ended question: governance of nursing agencies, client-related issues, and issues related to individual nurses. These topics overlap and are elaborated on below.

Fifteen of the comments related to issues of governance of nursing agencies, with 10 commenting on the perceived lack of support from the SANC when complaints about nurses were submitted or information about their registration was requested. They also commented on the SANC's lack of support for caregivers (lay health workers). Minor comments related to the policy uncertainty in light of the debates at the time regarding the proposed ban on labour brokers, lack of recognition of nursing agencies, and the high membership fees of ANASA, the voluntary association for nursing agencies.

Another set of comments related to the clients of nursing agencies, notably the high competition for clients among the various agencies, especially those owned by the large private hospital groups (*n=*7); lack of nurse orientation in some private hospitals (*n=*1); inability to meet clients’ demand (1); and racial discrimination or bias in selecting some agencies (*n* = 1).

With regard to individual nurses, agencies complained about the unreliability of nurses (*n=*4), the alleged misconduct of nurses (*n=*2), and the quality of care provided by nurses (*n=*1).

## Discussion

This study was done at a time of heated debate in South Africa on the future of labour brokers and on the negative impact of nursing agencies on the public health sector (26–28). The study found that the majority of agencies surveyed (77%) were established between 2000 and 2009. Because we do not have comparative data prior to this period, it is difficult to determine whether this number represents a significant increase in the number of agencies established during this period. One explanation could be that nursing agencies do not last very long, so that by the time of the survey only those that had been established in the past decade were still in business. One the other hand, the establishment of the agencies could be linked to the health workforce challenges experienced in the South African health system. For example, in 1994, there were 251 nurses per 100,000 population, compared to 110 per 100,000 in 2007; hence, fewer nurses were available relative to population size (30). As with all cross-sectional surveys, the temporal sequence between the establishment of a nursing agency and nursing shortages or possible growth in labour force casualisation could not be determined.

The study found that 32 (62%) of the surveyed agencies served private clients only and did not have any public-sector clients. Just over one-quarter (27%) of nursing agencies' clients were homes for elderly people, and geriatric care comprised an important component of the clinical services that these agencies provided. These findings suggest that homes for elderly people and geriatric care could be the niche areas of these smaller nursing agencies. It might be that ANASA members are the main providers of nursing services to large hospitals, particularly in the public sector, and that the smaller agencies could or would not compete with the larger agencies for the patronage of hospitals. As the findings of the ANASA study have not yet been analysed, it is not possible to compare the findings in this study with that of the ANASA study. However, any legislative or policy initiative on nursing agencies would need to take account of the nursing care needs of private homes or those for elderly people.

In this study, all but four nursing agencies (98%) indicated that they had a code of conduct, and 77% of agencies indicated that they had formal contracts with clients. Although this is a requirement of the Basic Conditions of Employment Act (BCEA), which states that labour brokers are to be jointly responsible for an employee (31), it is encouraging that so many nursing agencies reported the existence of a code of conduct. However, nursing agencies with private-sector clients were more likely to report formal contracts with clients (84%), compared to those who had formal contracts with non-private-sector clients (16%). This is of concern, because one would expect similar or greater accountability by public-sector clients for public monies spent on nursing agencies. As was the case with the nursing agency study in Australia (24), this study found that the predominant method that clients used to report complaints to nursing agencies was informal and verbal.

The study found that nurses or caregivers were allocated based on clients’ demands, rather than on the nurse or caregiver's preference or skills. Other studies have found that when nurses are not allocated to clients according to their preferences, the quality of care is compromised (19, 20, 32). This survey found that 81% of agencies agreed with a statement that they checked the SANC registration of nurses and 82% agreed with a statement that they requested certified copies of a nurse's qualifications. Of concern, however, is that almost one-fifth of agencies did not seem to comply with the basic quality checks of checking registration or requesting copies of qualifications. Only one-fifth of agencies (21%) indicated that they conducted reference checks with past employers. Hence this is an area that needs improvement, because the failure of a nursing agency to conduct these basic quality checks could lead to serious negative incidents both for individual clients and for the health system as a whole (33).

The highest reported challenges by agencies were nurse-related, notably shortages of specialised nurses (94%) and recruitment of nurses (83%). This suggests that the country's overall shortage of nurses, especially in specialised areas, also affects nursing agencies (34). This could lead to competition between agencies and health-care facilities for the employment of the limited number of specialised nurses, thus exacerbating the overall health workforce problems in South Africa.

This study has found that a monitoring system for agency nurses was lacking. In theory, a nurse could register and work with more than one agency within a short time period or the registration or qualifications of a nurse might not be checked, thus impacting on quality of care, a finding supported by an Australian study (12). The nursing agencies themselves raised the issue of governance in response to the open-ended question. The study findings point to the need for tighter management and regulation of agencies and improved monitoring and evaluation.

This study has revealed that this group of agencies that are not members of ANASA provided services to homes for elderly people and private homes, similar to the findings of a study done in a UK health district (35). Because 27% of nursing agencies provided services to homes for elderly people, that have remained outside the mainstream debates on labour brokers, these views would need to be incorporated into health policy development.

Despite careful planning and specific steps to minimise bias, which included doubling of the required sample size from 53 agencies to 106 agencies, a major weakness of the study was the low response rate of 49%. However, this response rate is much higher than an Australian study that had a response rate of 23% (24). In this study, non-operational agencies and no response to numerous phone calls accounted for 34% of non-respondents, indicating problems with the SANC database. These database problems arose due to a legislative vacuum, because SANC is no longer responsible for nursing agencies, which were classified as health establishments by the National Health Act (36).

The study did not ask for the reasons why these agencies were not operational, but one reason could be a lack of sustainability of smaller agencies. This is an area for further research. Only 15% of agencies contacted refused to participate, hence it is possible that with an accurate and updated database, the actual response rate would have been much higher. ANASA members were excluded, which limits the generalisability of the study findings to all nursing agencies.

Telephone surveys do not allow anonymity of responses, hence there could be social desirability bias, particularly in the responses to questions about the code of conduct and quality checks on nurses. Some of the questions might have been misunderstood by respondents and the person being interviewed may have had limited knowledge on the agency they worked for. The study was limited by the small sample size, which may explain some of the findings that were not statistically significant. In the analysis, some responses were not mutually exclusive, which limited the type of analysis conducted. Future studies regarding nursing agencies can build on the questionnaire used in this study and refine the questions to ensure that they are mutually exclusive.

Nevertheless, there are many study strengths. The focus is novel, and it is one of the first studies to focus on the nursing agency industry in South Africa. The findings provide a basis for future research on the nursing agency industry and sheds light on the characteristics of the industry. Selecting a stratified random sample, rather than a convenient sample of agencies, is a strength. The study provides unique information on agencies that are not part of ANASA.

There are a number of recommendations that flow from this study. A first step in strengthening the management of nursing agencies should be the development of a comprehensive database of all registered agencies in the country, which should be updated on a yearly basis. In terms of the National Health Act (36), a comprehensive set of regulations on nursing agencies should be developed, covering both the clients and staff contracted by the agencies. The guidelines should draw on best practice in other countries such as the United Kingdom (22, 23), as well the results of this study. The regulations should include a set of quality standards that are part of the national core standards (37).

Nursing agencies are health establishments (36) but are also labour brokers. Hence they face a dilemma of governance as they have to report to the National Department of Health (NDOH), the SANC, and the National Department of Labour (NDOL). There is a need for a consensus document that will outline the roles and responsibilities of the three organisations in regulating the industry. The NDOH could create a special unit to monitor the activities of nursing agencies annually. This special unit could be within the independent Office of Health Standards Compliance and could work together with a responsible unit at the NDOL to enforce labour standards. All agencies should be mandated to provide information to the responsible government institution on a set of core indicators that covers both their clients and the casual staff that they contract.

## Conclusion

The South Africa health system faces numerous problems of recruitment, management, and retention of health-care providers (4). Casualisation of the workforce compounds these challenges, especially in the public health sector. The study findings underscore the need for improved management, governance, and regulation of nursing agencies and enforcement of existing legislation. In the long term, there is a need for open policy debate on the future of nursing agencies to ensure that they meet the needs of the South African health system.

## References

[1] Global Health Workforce Alliance, WHO (2013). A universal truth: no health without a workforce.

[2] DOH (2011). Human Resources for Health South Africa: HRH strategy for the health sector 2012/13–2016/17.

[3] DOH (2013). National Strategic Plan for Nursing Education, Training and Practice 2012/13–2016/17.

[4] Rispel LC, Barron P (2012). Valuing human resources: key to the success of a national health insurance system. Dev South Afr.

[5] Kabene SM, Orchard C, Howard JM, Soriano MA, Leduc R (2006). The importance of human resources management in health care: a global context. Hum Resour Health.

[6] Rispel LC, Angelides G (2014). Utilisation and costs of nursing agencies in the South African public health sector, 2005–2010. Glob Health Action.

[7] Rispel LC, Blaauw D, Chirwa T, de Wet K (2014). Factors influencing agency nursing and moonlighting among nurses in South Africa. Glob Health Action.

[8] Rispel LC, Chirwa T, Blaauw D (2014). Does moonlighting influence South African nurses’ intention to leave their primary jobs?. Glob Health Action.

[9] ILO (2006). Changing patterns in the world of work: report of the director-general to the International Labour Conference, 95th session.

[10] Webster E, Van Holdt K (2005). Beyond the apartheid workplace: studies in transition.

[11] Buchan J, O'May F (1999). Globalisation and healthcare labour markets: a case study from the United Kingdom. HRDJ.

[12] Creegan R, Duffield C, Forrester K (2003). Casualisation of the nursing workforce in Australia: driving forces and implications. Aust Health Rev.

[13] Storch JL (1998). Casualisation of nurses and unregulated workers impair ethical practice. Int Nurs Rev.

[14] Republic of South Africa (2005). Nursing Act No. 33 of 2005.

[15] Adams B (1989). Agency nursing: the hospital's response. J Nurs Adm.

[16] Braddy PK, Washburn TA, Caroll LL (1991). Factors influencing nurses to work for agencies. West J Nurs Res.

[17] Hughes KK, Marcantonio RJ (1991). Recruitment, retention and compensation of agency and hospital nurses. J Nurs Adm.

[18] Jackson BS (1989). Agency nursing: costs and quality [Opinion]. J Nurs Adm.

[19] Manias E, Aitken R, Peerson A, Parker J, Wong K (2003). Agency-nursing work: perceptions and experiences of agency nurses. Int J Nurs Stud.

[20] Manias E, Aitken R, Peerson A, Parker J, Wong K (2003). Agency nursing work in acute care settings: perceptions of hospital nursing managers and agency nurse providers. J Clin Nurs.

[21] Manion J, Reid S (1989). Managing temporary staffing services: selecting the right agency. Nurs Econ.

[22] National Audit Office (2006). Improving the use of temporary nursing staff in NHS acute and foundation trusts.

[23] National Audit Office (2006). Good practice in managing the use of temporary nursing staff.

[24] Peerson A, Aitken RL, Manias E, Parker JM, Wong K (2002). Agency nursing in Melbourne, Australia: a telephone survey of hospital and agency managers. J Adv Nurs.

[25] Rispel L (2008). Research on the State of Nursing (RESON): exploring nursing policies, practice and management in South Africa – Workshop Report.

[26] COSATU (2009). COSATU, FAWU, NEHAWU, NUM, NUMSA, SACCAWU and SATAWU submission on labour broking. http://www.cosatu.org.za/show.php?ID=2237.

[27] Ramutloa L (2011). Media briefing on the status of labour brokers and the NEDLAC negotiations on Labour Law Amendments by Labour Minister, Mildred Oliphant. http://www.labour.gov.za/media-desk/speeches.

[28] ANASA (2010). 2010 Newsletter: labour Broker Ban. http://anasa.org.za/documents/Newsletter-9.pdf.

[29] Miles M, Huberman AM, Saldaña J (2014). Qualitative data analysis: a methods sourcebook.

[30] Lehmann U, Barron P, Roma-Reardon J (2008). Strengthening human resources for primary health care. South African Health Review 2008.

[31] Republic of South Africa (1997). Basic Conditions of Employment Act No. 75.

[32] Massey L, Esain A, Wallis M (2009). Managing the complexity of nurse shortages: a case study of bank and agency staffing in an acute care Trust in Wales, UK. Int J Nurs Stud.

[33] Rispel L, Armstrong S, Blaauw D, Chirwa T, Engelbrecht M, de Wet K (2011). The nature and health system consequences of casualisation, agency nursing and moonlighting in South Africa.

[34] Scribante J, Bhagwanjee S (2007). National audit of critical care resources in South Africa – nursing profile. S Afr Med J.

[35] Bennett J (1986). Private nursing homes: contribution to long stay care of the elderly in the Brighton Health District. Br Med J (Clin Res Ed).

[36] Republic of South Africa (2005). National Health Act No. 61 of 2003.

[37] Department of Health (2011). “Towards quality care for patients”. National core standards for health establishments in South Africa.

# Sub article: ‘Practice what you preach’: Nurses’ perspectives on the Code of Ethics and Service Pledge in five South African hospitals

**DOI:** 10.3402/gha.v8.26341

**Published:** 2015-05-11

**Authors:** Janine White, Maureen Phakoe, Laetitia C. Rispel

**Affiliations:** 1 School of Public Health, Faculty of Health Sciences, University of the Witwatersrand, Johannesburg, South Africa; 2 Centre for Health Policy & Medical Research Council Health Policy Research Group, School of Public Health, Faculty of Health Sciences, University of the Witwatersrand, Johannesburg, South Africa

**Keywords:** code of ethics, nurses pledge, nurses, ethical dilemmas, South Africa

## Abstract

**Background:**

A recent focus of the global discourse on the health workforce has been on its quality, including the existence of codes of ethics. In South Africa, the importance of ethics and value systems in nursing was emphasised in the 2011 National Nursing Summit.

**Objective:**

The study explored hospital nurses’ perceptions of the International Code of Ethics for Nurses; their perceptions of the South African Nurses’ Pledge of Service; and their views on contemporary ethical practice.

**Methods:**

Following university ethics approval, the study was done at a convenience sample of five hospitals in two South African provinces. In each hospital, all day duty nurses in paediatric, maternity, adult medical, and adult surgical units were requested to complete a self-administered questionnaire. The questionnaire focused on their perceptions of the Code of Ethics and the Pledge, using a seven-point Likert scale. STATA^®^ 13 and NVIVO 10 were used to analyse survey data and open-ended responses, respectively.

**Results:**

The mean age of survey participants (*n*=69) was 39 years (SD=9.2), and the majority were female (96%). The majority agreed with a statement that they will promote the human rights of individuals (98%) and that they have a duty to meet the health and social needs of the public (96%). More nuanced responses were obtained for some questions, with 60% agreeing with a statement that too much emphasis is placed on patients’ rights as opposed to nurses’ rights and 32% agreeing with a statement that they would take part in strike action to improve nurses’ salaries and working conditions. The dilemmas of nurses to uphold the Code of Ethics and the Pledge in face of workplace constraints or poor working conditions were revealed in nurses’ responses to open-ended questions.

**Conclusion:**

Continuing education in ethics and addressing health system deficiencies will enhance nurses’ professional development and their ethical decision-making and practice.

A recent focus of the global discourse on the health workforce has been on its quality, including evidence of regulatory bodies in countries and the existence of codes of ethics (1). Ethical standards are common in the healthcare professions and are operationalised through codes of ethics and/or service pledges (2, 3). A code of ethics governs professional conduct and is a symbolic written expression, whereas a pledge is the verbal expression of ethical considerations in professional conduct (4). The code of ethics and practice of pledges have their origins in the deontological view that it is a nurse's responsibility to act in the best interest of individuals in their care. This view suggests that professional values influence nurses’ behaviour in practice, and assumes that there is a direct link between nurses’ awareness and understanding of the code and pledge and their ethical behaviour and practice (4, 5).

The International Code of Ethics for Nurses was developed in 1953 by the International Council for Nurses (ICN) (6). The Code has undergone four revisions, with the most recent revision in 2012. The ICN Code is organised into four fundamental guiding elements: nurses and people, nurses and practice, nurses and the profession, and nurses and co-workers (6). The first element, nurses and people, focuses on nurses’ interactions with people in the healthcare setting. It upholds the advancement of the human rights of individuals, family, and community; the dissemination of information for decision-making; and the protection of confidential information (6). The second element, nurses and practice, links professional nursing practice with personal responsibility and accountability, and highlights the importance of continuing education (6). The third element, nurses and the profession, emphasises the nurse's role in the implementation of acceptable standards of clinical practice, while the fourth element, nurses and co-workers, is concerned with the relationship between the individual nurse and other co-workers, the protection of individuals, families, and communities when a health threat is identified, and the promotion of ethical conduct among co-workers (6).

The International (Nightingale) Nurses’ Pledge is a promise undertaken by nurses to uphold the core values and principles of nursing, and is aligned to the ICN Code of Ethics. It refers to promoting the rights of patients and the promotion of health, protection of information, refraining from endangering the life and health of patients, maintaining professional competence and continuing education, and sustaining good working relationships with colleagues (7). The Pledge is commonly recited at nursing graduation ceremonies around the world, including in South Africa.

In South Africa, the South African Nursing Council (SANC) Pledge of Service (also referred to as the Nurses’ Pledge or the Pledge) is an adapted version of the Nightingale Pledge (8). The SANC, established in terms of the Nursing Act (9), is the regulatory body for all categories of nurses (including midwives) and is responsible for setting standards for professional and ethical nursing practice, and nursing education and training throughout South Africa (10). Hence, the education of all professional nurses with 4 years of training includes a mandatory ethics component, prior to registration with the SANC.

The importance of ethics and value systems in nursing was emphasised at the 2011 National Nursing Summit, that brought together close to 2000 nurses in South Africa (11). The final report of the Ministerial Task Team on Nursing Education, Training and Practice contains key recommendations on ‘restoring ethics and respect in nursing’ and the ‘mainstreaming of ethics in all nursing training programmes’ (11).

Notwithstanding the importance of ethics and value systems in nursing, the Code of Ethics and Nurses’ Pledge are not without controversy, particularly in the context of industrial action by nurses to achieve enhanced status for the profession, improved career paths, increased salaries, and better quality of care for patients (12–16). Nonetheless, there are several studies that have focused on Codes of Ethics for nurses in different country contexts (14, 17–23), ethical dilemmas faced by nurses in the workplace (5, 24–28), and the ethical issues involved in industrial action by nurses (12, 13, 15, 16, 29, 30). Those studies that have focused on Codes of Ethics have found that there is widespread support among nurses as these Codes serve as a guide to ethical decision-making and behaviour (14, 17–22). However, some scholars have pointed out that these Codes do not assist with ethical dilemmas in the workplace or with solving the resource constraints and poor working conditions faced by nurses (14, 18, 24, 31). This is exacerbated by lack of awareness of Codes of Ethics and sub-optimal knowledge among nurses of what constitutes good ethical practice (19, 21, 31, 32).

In South Africa, a number of studies have focused on the tensions between nurses’ ethical values or conduct and industrial action (12, 29, 30), or ethical values and termination of pregnancy (28). In light of the renewed focus of the South African government on the nursing profession and the call for the implementation of ‘a comprehensive programme to restore ethics and respect in nursing’ (11), the aim of this study was to explore nurses’ perspectives on the ICN Code of Ethics and the SANC Nurses’ Pledge in a convenience sample of hospitals in Gauteng and Free State Provinces of South Africa.

## Methods

The study was nested in a larger project that examined the nature and dynamics of nursing management and quality of care in hospitals. The larger study focused on nine randomly selected hospitals in Gauteng (urban) and Free State (mixed urban–rural) Provinces in South Africa. The choice of the two provinces, Gauteng and the Free State, was purposive, and influenced by geographical proximity to the researchers, prior health authority approval, and budgetary constraints. Gauteng is the most urbanised and densely populated province with a population of 12.2 million, while the Free State Province is a mixed urban–rural, largely agricultural province, with a population of 2.7 million (33).

Because the larger study was already underway when the ethics protocol was finalised, the ethics component of the study was done in the two remaining private hospitals in Gauteng Province, and the three public hospitals in Free State Province. Hence the five hospitals, although randomly selected as part of the larger study, could be considered as a convenience sample for the ethics study.

The Human Research Ethics Committee (Medical) of the University of the Witwatersrand in Johannesburg provided ethics approval for the study. The relevant public and private health care authorities also gave the necessary study approvals. All participants received a study information sheet and provided written, informed consent.

At each hospital, one medical, surgical, paediatric, and maternity (labour and post-natal) unit was selected (total four units per hospital); hence, 20 units were part of the study. Emergency and critical care units were excluded because the focus of the larger study was on nursing unit managers. In the ethics study, the population of interest was all categories of nurses registered with the SANC. On the survey day, all day duty nurses working in the selected units were invited to participate in the study. Night duty nurses were excluded because of logistical difficulties in conducting fieldwork at night at the selected hospitals.

Following informed consent, each nurse completed a self-administered questionnaire that contained both closed and open-ended questions. The questionnaire consisted of four sections. The first three sections contained closed-ended questions that elicited information on: participant characteristics; perspectives on the ICN Code of Ethics; and perspectives on the South African Nurses’ Pledge of Service. The Code of Ethics section in the questionnaire consisted of 14 questions, which related to each of the four elements of the ICN Code (6). The section on the Nurses’ Pledge consisted of nine questions, which related to the elements of the Pledge (7). The questions were designed on a seven-point Likert scale ranging from 1 (strongly disagree) to 7 (strongly agree). The questions on the Nurses Code of Ethics and Nurses’ Pledge were phrased in a manner that attempted to minimise an unreflective response by participants. The fourth section consisted of three open-ended questions that focused on the opinions of participants on current ethical practice of nurses, strategies for enhancing ethical practice, and any other comments on the Code of Ethics or Nurses’ Pledge. The questionnaire was piloted prior to implementation, and no changes were deemed necessary.

We analysed the quantitative data using STATA^®^ 13. Frequency tabulations were done to describe the socio-demographic characteristics of the respondents, and the responses to questions on the Nurses’ Code of Ethics and Nurses’ Pledge. A thematic content analysis of transcripts was conducted (34) using NVIVO version 10 software for the qualitative data analysis. Two members of the research team (LCR and JW) coded the open-ended questions independently and then established inter-coder agreement. Once the codes were agreed to, the transcripts were loaded into the software, and the coding of each transcript was done to identify recurring themes. To ensure the trustworthiness of the data, continuous peer debriefing and checking of researchers’ interpretations against the raw data was done.

## Results

The study recruited 69 nurses of all categories in the five hospitals. There were no refusals, representing a 100% response rate.

### Participant characteristics

The age of participants ranged from 23 to 61 years, with a mean age of 39 years (SD 9.2). The majority of participants were female (96%) and employed in public hospitals (61%) (Table 1).

**Table 1:** Demographic and employment characteristics of study participants

Characteristic	Total^a^
Number of participants	69
Mean age (standard deviation)	39 (9.2)
<29 years (%)	16 (23)
30–39 (%)	20 (29)
40+ years (%)	33 (48)
Sex	
Female (%)	66 (96)
Male (%)	3 (4)
Marital status	
Married (%)	32 (46)
Living together (%)	4 (6)
Single (%)	28 (41)
Divorced/separated (%)	1 (1)
Widowed (%)	4 (6)
Category of nurse	
Professional nurse (%)	23 (35)
Enrolled nurse (%)	13 (19)
Auxiliary nurse (%)	17 (25)
Category not specified	15 (22)
Sector of employment	
Provincial hospital (%)	42 (61)
Private hospital (%)	27 (39)
Unit of work	
Paediatric (%)	9 (13)
Medical (%)	17 (25)
Surgical (%)	17 (25)
Maternity (%)	19 (27)
Other (%)	5 (7)

a Minor discrepancies due to missing values.

### Nurses’ opinions on the ICN Code of Ethics

Table 2 shows the participants’ opinions on the 14 questions that explored their opinions on the ICN Code of Ethics, ranked in order of the level of agreement.

**Table 2:** Nurses’ opinions on the ICN Code of Ethics

Statement	*n* ^a^	Agree %	Neutral %	Disagree %
I play an active role to maintain good relationships with my co-workers	69	100	0	0
I promote the human rights of individuals under my care	66	98	2	0
I always maintain the standards of personal conduct required by my profession	69	96	0	4
I have a duty to meet the health and social needs of the public	68	96	1	3
I get upset when I do not have equipment to provide good patient care	68	95	0	5
I think too much emphasis is placed on patients’ rights at expense of nurses’ rights	65	60	10	30
I will take part in strike action to improve nurses’ salaries	68	32	6	62
I believe that a trade union is better than a professional organisation to improve nurses’ socio-economic conditions	68	26	19	55
I would provide information on a patient's HIV positive status to his/her family	69	22	2	76
It is not my responsibility to change the image of nursing	68	19	2	79
Patients should not receive a lot of information about their care as it confuses them	65	17	2	81
It is not my role to implement acceptable standards of clinical nursing practice	69	10	2	88
Providing care to homosexuals is against my ethical values	67	6	8	86
It is not my own responsibility to maintain professional competence	66	6	0	94

a Discrepancies due to missing values.

As can be seen from the table, 100% of respondents agreed with the statement ‘I play an active role to maintain good relationships with co-workers’. High levels of agreement were found for promotion of the human rights of the people in their care (95%); the nurses’ duty to meet the health and social needs of the public (96%); maintaining the standards of personal conduct required by the nursing profession (96%); and ‘getting upset’ when lacking necessary equipment to provide good patient care (95%).

Similarly, high levels of disagreement were found in negative statements such as ‘nurses do not have a responsibility to maintain professional competence’ (94%), ‘it is not my role to implement acceptable standards of clinics nursing practice’ (88%), ‘providing care to homosexuals is against my ethical values’ (86%) and ‘patients should not receive a lot of information about their care as it confuses them’ (81%). More nuanced responses were obtained for the three questions that elicited responses on nurses versus patients’ rights, strike action, and a trade union versus a professional association (Table 2).

### Nurses’ opinions on the Pledge of Service

Table 3 shows the participants opinions on the nine questions that explored their opinions on the Nurses’ Pledge, ranked in order of the level of agreement.

**Table 3:** Nurses’ opinions on the Pledge of Service

Statement	*n* ^a^	Agree %	Neutral %	Disagree %
I care for sick patients with all the skill and understanding I possess	66	100	0	0
I recited the Nurses’ Pledge with pride	66	98	0	2
I make efforts to keep my professional knowledge and skills at the highest level	68	98	0	2
I hold in confidence all personal information given over to me	65	93	0	7
I am passionate about alleviating suffering	67	77	3	20
I cannot uphold the integrity of the professional nurse	66	17	0	83
Race influences the way I take care of patients	66	9	3	88
I cannot respect the religious beliefs of patients under my care	68	3	0	97
People who pay for their care should get better services than those who do not pay	68	3	3	94

a Discrepancies due to missing values.

As can be seen from Table 3, 100% of respondents agreed with the statement ‘I care for sick patients with all the skill and understanding that I possess’. High levels of agreement were found for statements on reciting the Nurses’ Pledge with pride (98%); and making effort to keep the highest level of professional knowledge and skills (98%).

Similarly, high levels of disagreement were found in the negative statement ‘I cannot respect the religious beliefs of patients under my care’ (97%) and that ‘People who pay for their care should get better services than those who do not pay’ (94%).

### Opinions on contemporary ethical practice of nurses

Although overlapping, three broad themes emerged from nurses’ responses to the open-ended questions. These were: insufficient awareness or knowledge on what constitutes good ethical practice; the need for a strong ethics focus in nursing education and training; and the dilemma of nurses to uphold the code of ethics and service pledge in face of workplace constraints or poor working conditions.

The majority of participants were of the opinion that there is insufficient awareness or knowledge among students or younger nurses on what constitutes good ethical practice.

Nurses of today have got no clue of the word ethics. One could see with the way they lack professionalism, etiquette and confidentiality. [Respondent 13, Gauteng private hospital 2]

The Nurses’ Pledge is only for registered or professional nurses, other categories of nursing do not know about it, but they are also practising as nurses. They should also be taught about ethics and pledge. [Respondent 57, Free State public hospital 2]

The study participants were also of the opinion that nursing education and training programmes should place a greater emphasis on ethical behaviour and practice.

The ethical practice must be improved. All [nurses] must know about the rules and regulations and the Code of Ethics. [Respondent 21, Gauteng private hospital 2]

I think they must start in the colleges, the learning centres – they must start to tell these people what is ethics, because I don't think they know – they write exams, but I don't think they know how to practise this in the operational field. [Respondent 37, Free State public hospital 1]

Training and education on ethical practice in nursing should be done regularly to all nursing personnel, maybe after every six months. [Respondent 51, Free State public hospital 2]

Ethical dilemmas mentioned by study participants included: providing care in the face of disrespect from patients, the problems created by individuals who do nursing for the wrong reasons; and being blamed by managers for errors or mistakes, despite staff shortages or health system deficiencies, especially in public hospitals.

Some respondents were of the opinion that patients have a ‘bad attitude towards nurses’, and their disrespectful behaviour, makes it more difficult for nurses to practise ethically. Several study participants lamented about the perceived ‘poor’ calibre of new entrants to the nursing profession, as can be seen from the following comments:

New nurses’ attitudes are completely different than 20 years ago. There is need for a complete turnaround and thorough sifting of individuals before they enter the [nursing] profession. [Respondent 9, Gauteng private hospital 1]

I think the problem lies with the nurses that start to nurse for the wrong reasons – nursing has become a money-spinning thing, but the deep passion and that deep born thing for caring is missing. [Respondent 37, Free State public hospital 1]

Some study respondents, particularly in public hospitals, mentioned health system deficiencies as hindering ethical practice by nurses.

The ethical practice of nurses is not up to standard due to shortage of staff. Hire more staff to boost nurses’ morale and reduce stress periods. [Respondent 38, Free State public hospital 2]

People lose [their] morale in the nursing profession. There must be enough staff, equipment, and nurses must get enough support from the government–financially and psychologically. [Respondent 40, Free State public hospital 2]

### Strategies to enhance ethical practice in nursing

The overwhelming number of responses related to a ‘practice what you preach’ approach to the Code of Ethics and the Pledge, illustrated by the comment below:

Nursing students should practise professionalism at an early level in the college or university. All nursing educators and registered nurses must be role models for nursing students and other subordinates, such as enrolled nurses. [Respondent 64, Free State public hospital 3]

Recommendations included the need for continuous professional education on ethical behaviour and practice; using health service events (e.g. hospital open days) to remind nurses of good ethical practice; and addressing nurses’ salaries and working conditions, resource constraints and health system deficiencies such as the lack of functioning equipment.

## Discussion

This study found that there were high levels of awareness among nurses in the selected hospitals on the Code of Ethics and the Pledge, illustrated by their responses to a series of proxy statements on the Code of Ethics and Nurses’ Pledge. The questions on the Code of Ethics (Table 2) elicited high levels of agreement on the statements regarding the maintenance of good relationships with co-workers, the promotion of the human rights of patients, the nurses’ duty to meet the health and social needs of the public, maintaining the standards of personal conduct required by the nursing profession, and ‘getting upset’ when lacking necessary equipment to provide good patient care. Similarly, high levels of disagreement were found in negative statements where such disagreement was appropriate. The statements on the Nurses’ Pledge of Service (Table 3), also found high levels of agreement on caring for sick patients with the necessary skill and understanding, reciting the Nurses’ Pledge with pride; and making effort to keep the highest level of professional knowledge and skills. Although not directly comparable, other studies have also found high levels of awareness of and support for Codes of Ethics to guide ethical decision-making and behaviour (14, 17–22).

More nuanced responses were obtained for the three questions that elicited responses on nurses versus patients’ rights, strike action, and a trade union versus a professional association. The majority of respondents (60%) agreed with the statement ‘I think too much emphasis is placed on patients’ rights at the expense of nurses’ rights’. This was borne out by the qualitative comments made in the open-ended questions, with some nurses indicating that perceived disrespect from patients influenced their ability to provide optimal quality of care. The finding could reflect the emphasis on the government's core national standards that aim to improve patients’ experiences of public sector care at the time of the study (35), and misunderstanding on the part of nurses regarding the overall goal of the national core standards.

Although a minority of nurses agreed with the statements that they will embark on strike action to improve nurses’ salaries (32%) and that a trade union is better than professional association to improve the socio-economic conditions of nurses (26%), the results are not surprising. Another South African study also found that 32.5% of study participants supported strike action by nurses as a constitutional and legal right (29). Studies in other countries have also found that although strike action is not an easy decision, nurses will embark on industrial action to achieve improvements in the health care system and in their own working conditions (13, 15).

The three themes that emerged from the analysis of the open-ended questions: insufficient awareness or knowledge on what constitutes good ethical practice; the need for a strong ethics focus in nursing education and training; and the dilemma of nurses to uphold the Code of Ethics and the Pledge in the face of workplace constraints or poor working conditions contradicted the participants’ responses to the closed-ended questions. There are several reasons for this apparent contradiction. Firstly, the responses might be a reflection of social desirability bias (36), with participants giving responses that they thought the researchers wanted to hear, or that were appropriate at the time of the study. Secondly, the contradictory responses might reflect the disjuncture between their levels of awareness and the difficulties of translating that awareness or knowledge into practice. Thirdly, it might be that ethics is dealt with in pre-service nursing training, but that there is little focus and discussion on dealing with ethical issues in the workplace.

This was one of the first studies in South Africa to explore nurses’ views on the ICN Code of Ethics and the Nurses’ Pledge, following the 2011 National Nursing Summit in the country. The paper makes an important contribution to the discourse on ethical behaviour and practice of nurses and midwives. However, the findings are not generalisable as the study was small and limited to five South African hospitals that constituted a convenience sample. Although the statements that served as a proxy to determine nurses’ views on the Code of Ethics and the Pledge of Service were phrased carefully to avoid non-reflective responses, the contradictory responses indicate some social desirability bias (36). The cross-sectional design means that the study reflects the views of nurses at a point in time. Nonetheless, the study provides valuable insights into nurses’ perspectives on the International Code of Ethics and the Nurses’ Pledge of Service, and opens a scholarly discourse on ethics in South Africa. Further research on ethics is needed, which focuses on a more representative sample of nurses and which examines possible variations in the perceptions and ethical dilemmas faced by nurses between urban and rural areas, and between the public and private health sectors.

Notwithstanding the limitations of the study, the findings support the recommendations contained in the strategic plan on Nursing Education, Training and Practice (11). These recommendations include: raising awareness on ethics as a means to strengthen ethical nursing practice; ‘core, compulsory modules at all levels of nursing and midwifery training’ that emphasise professionalism, ethics and caring; and the introduction of a ‘Continuing Professional Development (CPD) system for all nurses and midwives, linked to licensing and professional progression, and which includes professionalism and ethics as a compulsory component’ (11, p. 7).

Other studies have also recommended raising awareness of professionalism and ethical behaviour as a means of strengthening ethical nursing practice (23, 37). Our study participants expressed concern with the quality and content of nursing ethics at undergraduate level. They recommended that greater emphasis should be placed on ethics at undergraduate level, but that ethics should be linked clearly to the competencies of nurses and their ability to provide good quality of care and navigate their way through ethical dilemmas. However, the SANC is the regulator of nursing and midwifery standards of practice, education and training in South Africa and emphasises the ethical and moral obligations of nurses in performing their duties (9). These obligations are expressed as Regulations to the Nursing Act (9). The participants’ perceptions of the inadequacy of ethics training in pre-service nursing education and training could be related to the policy-implementation gaps that are well described (38, 39). A study in Thailand found that appropriate didactic methods were necessary to create a learning environment that promotes ethical practice and appropriate behaviour of nursing students (37). The authors recommended additional training of new graduate nurses in decision-making and how to manage ethical dilemmas in nursing practice (37).

However, some scholars have pointed out that raising awareness is important, but that a holistic understanding of ethical practice includes the four components of ethical sensitivity, ethical judgement, ethical motivation and ethical action (5). Specific strategies recommended include CPD that focuses on the four component model, the establishment of nursing ethics groups, nurses’ participation in inter-disciplinary ethics rounds, the possible introduction of nursing ethics ward rounds, seeking guidance or support from senior or experienced nursing or medical colleagues, and a wider discourse on ethical nursing practice (5, p. 69).

Other nursing scholars have warned about the imposing position that pledges and oaths may bring (40) and have recommended a change leadership approach within nursing that combines visible, ethical leadership with participation of front-line nurses (40). Such leadership includes appropriate role modelling, which emphasises again the ‘practice what you preach’ approach, suggested by many study participants.

Importantly, the health system deficiencies alluded to by study participants would need to be addressed in order to facilitate ethical nursing practice. The appointment of the Chief Nursing Officer at the beginning of 2014 and a detailed strategic plan for Nursing Education, Training and Practice provide a good foundation in South Africa for strengthening ethics training and enhancing ethical nursing practice.

## Conclusion

In light of the numerical dominance of nurses in South Africa, and their role in the health and well-being of patients and communities, the importance of their technical and ethical competence is undisputed. The study participants displayed high levels of awareness of the ICN Code of Ethics and Nurses’ Pledge of Service. However, the responses revealed contradictions between knowledge and awareness, and contemporary nursing practice within a value-based, ethical framework. Continuing education in ethics and addressing health system deficiencies will enhance nurses’ professional development and their ethical decision-making and practice.

## References

[1] Global Health Workforce Alliance, WHO (2013). A universal truth: no health without a workforce.

[2] Leach MM, Harbin J (1997). Psychological ethics codes: a comparison of twenty-four countries. Int J Psychol.

[3] Wilson-Barnett J (1986). Ethical dilemmas in nursing. J Med Ethics.

[4] Yeun E-J, Kwon Y-M, Kim H-K (2004). A Q-methodological study on nursing students’ attitudes toward nursing ethics. Taehan Kanho Hakhoe Chi.

[5] Robichaux C (2012). Developing ethical skills: from sensitivity to action. Crit Care Nurse.

[6] ICN (2012). The International Code of Ethics for Nurses.

[7] ICN The International Nurses Pledge.

[8] SANC The Nurses Pledge of Service.

[9] Republic of South Africa (2005). Nursing Act No. 33 of 2005.

[10] Breier M, Wildschut A, Mgqolozana T (2009). Nursing in a new era: the profession and education of nurses in South Africa.

[11] DOH (2013). Strategic Plan for Nursing Education, Training and Practice 2012/13–2016/17.

[12] Heunis C, Pelser AJ (1997). Down the needle! Should public sector nurses in South Africa have the right to strike?. Curationis.

[13] Strachan G (1997). Not just a labour of love: industrial action by nurses in Australia. Nurs Ethics.

[14] Biton V, Tabak N (2003). The relationship between the application of the nursing ethical code and nurses’ work satisfaction. Int J Nurs Practice.

[15] Tabak N, Wagner N (1997). Professional solidarity versus responsibility for the health of the public: is a nurses’ strike morally defensible?. Nurs Ethics.

[16] Brown GD, Greaney A-M, Kelly-Fitzgibbon ME, McCarthy J (2006). The 1999 Irish nurses’ strike: nursing versions of the strike and self-identity in a general hospital. J Adv Nurs.

[17] Dobrowolska B, Wronska I, Fidecki W, Wysokinski M (2007). Moral obligations of nurses based on the ICN, UK, Irish and Polish codes of ethics for nurses. Nurs Ethics.

[18] Hussey T (1996). Nursing ethics and codes of professional conduct. Nurs Ethics.

[19] Tadd W, Clarke A, Lloyd L, Leino-Kilpi H, Strandell C, Lemonidou C (2006). The value of nurses’ codes: European nurses’ views. Nurs Ethics.

[20] Verpeet E, Dierckx de Casterlé B, Lemiengre J, Gastmans CAE (2006). Belgian nurses’ views on codes of ethics: development, dissemination, implementation. Nurs Ethics.

[21] Verpeet E, Dierckx de Casterle B, Van der Arend A, Gastmans CAE (2005). Nurses’ views on ethical codes: a focus group study. J Adv Nurs.

[22] Zahedi F, Sanjari M, Aala M, Peymani M, Aramesh K, Parsapour A (2013). The Code of Ethics for Nurses. Iran J Public Health.

[23] Miller B, Beck L, Adams D (1991). Nurses’ knowledge of the code for nurses. J Contin Educ Nurs.

[24] Dierckx de Casterlé B, Izumi S, Godfrey NS, Denhaerynck K (2008). Nurses’ responses to ethical dilemmas in nursing practice: meta-analysis. J Adv Nurs.

[25] Donnelly PL (2000). Ethics and cross-cultural nursing. J Transcult Nurs.

[26] Creel EL, Robinson JC (2010). Ethics in independent nurse consulting: strategies for avoiding ethical quicksand. Nurs Ethics.

[27] Turale S (2014). Ethical dilemmas: the challenge of advocating for human rights. Int Nur Rev.

[28] Botes A (2000). Critical thinking by nurses on ethical issues like the termination of pregnancies. Curationis.

[29] Muller M (2001). Strike action by nurses in South Africa: a value clarification. Curationis.

[30] Van Rensburg AJ, van Rensburg DJ (2013). Nurses, industrial action and ethics: considerations from the 2010 South African public-sector strike. Nurs Ethics.

[31] Wolf ZR, Zuzelo PR (2006). “Never again” stories of nurses: dilemmas in nursing practice. Qual Health Res.

[32] Numminen OH, Leino-Kilpi H, van der Arend A, Katajisto J (2009). Nursing students and teaching of codes of ethics: an empirical research study. Int Nurs Rev.

[33] Statistics South Africa (2012). Census 2011: statistical release (revised) P0301.4.

[34] Green J, Thorogood N (2004). Qualitative methods for health research.

[35] DOH (2010). National core standards for health establishments in South Africa.

[36] Randall DM, Fernandes MF (1991). The social desirability response bias in ethics research. J Bus Ethics.

[37] Chaowalit A, Hatthakit U, Nasae T, Suttharangsee W, Parker M (2002). Exploring ethical dilemmas and resolutions in nursing practice: a qualitative study in Southern Thailand. Thai J Nurs Res.

[38] Rispel L, Moorman J, Daniel J, Naidoo P, Pillay D, Southall R (2013). Health policy reforms and policy implementation in South Africa: a paradox?. New South African Review 3: the second phase-tragedy or farce?.

[39] Van Rensburg HCJ (2012). Health and health care in South Africa.

[40] Gallagher A (2013). Values for contemporary nursing practice: waving or drowning?. Nurs Ethics.

# Sub article: Using diaries to explore the work experiences of primary health care nursing managers in two South African provinces

**DOI:** 10.3402/gha.v7.25323

**Published:** 2014-12-22

**Authors:** Pascalia O. Munyewende, Laetitia C. Rispel

**Affiliations:** Centre for Health Policy & Medical Research Council Health Policy Research Group, School of Public Health, Faculty of Health Sciences, University of the Witwatersrand, Johannesburg, South Africa

**Keywords:** primary health care, nursing managers, diary methodology, health reforms, South Africa

## Abstract

**Background:**

South Africa is on the brink of another wave of major health system reforms that underscore the centrality of primary health care (PHC). Nursing managers will play a critical role in these reforms.

**Objective:**

The aim of the study was to explore the work experiences of PHC clinic nursing managers through the use of reflective diaries, a method hitherto under-utilised in health systems research in low- and middle-income countries.

**Methods:**

During 2012, a sub-set of 22 PHC nursing managers was selected randomly from a larger nurses’ survey in two South African provinces. After informed consent, participants were requested to keep individual diaries for a period of 6 weeks, using a clear set of diary entry guidelines. Reminders consisted of weekly short service messages and telephone calls. Diary entries were analysed using thematic content analysis. A diary feedback meeting was held with all the participants to validate the findings.

**Results:**

Fifteen diaries were received, representing a 68% response rate. The majority of respondents (14/15) were female, each with between 5 and 15 years of nursing experience. Most participants made their diary entries at home. Diaries proved to be cathartic for individual nursing managers. Although inter-related and not mutually exclusive, the main themes that emerged from the diary analysis were health system deficiencies; human resource challenges; unsupportive management environment; leadership and governance; and the emotional impact of clinic management.

**Conclusion:**

Diaries are an innovative method of capturing the work experiences of managers at the PHC level, as they allow for confidentiality and anonymity, often not possible with other qualitative research methods. The expressed concerns of nursing managers must be addressed to ensure the success of South Africa's health sector reforms, particularly at the PHC level.

Primary health care (PHC) has re-occupied centre stage in the global efforts towards universal coverage and improved health system performance (1, 2). Within this context, there is global recognition that competent managers are essential for ensuring that priority health needs are met, quality health services are delivered, and that resources are used effectively (3–7). Health managers play a strategic role in planning, allocating resources, and monitoring health policy targets and outcomes (6, 8). At an operational (hospital ward or clinic) level, managers are responsible for effective service delivery (6, 8).

South Africa is on the brink of another wave of major health sector reforms towards universal health coverage that underscore the centrality of PHC (9, 10). Nurses are the single largest category of trained health workers, and they play a crucial role in the current provision of PHC services and the management of the existing network of more than 3,000 government PHC clinics and community health centres (11). The PHC re-engineering strategy is one of the major health sector reforms designed to improve population health outcomes and the performance of the health care system (9, 12). These reforms acknowledge the critical role of nurses at the PHC level, both as members of multi-disciplinary clinical teams, and as managers of the community-based outreach teams and school health services (11, 13).

There is a plethora of literature on management and the different conceptual approaches to management (14–18). The management concept has also assumed increasing importance in the health sector (19, 20), with an entire WHO series focusing on different aspects of management strengthening (1, 4, 6, 8). In the health sector, studies have focused on district health managers (i.e. individuals in-charge of an entire health district), and range from a description of socio-demographic characteristics of managers (6), their roles and responsibilities (4, 6, 21), relationships between district managers and their staff, through to assessments of their competencies (4, 6, 21–23). The study findings suggest that there is a general lack of appreciation of managers as a critical component of the health workforce (4, 6, 21). In South Africa, a national assessment of district management structures, competencies, and training programmes found several shortcomings, including incomplete restructuring initiatives, over-extended staff, sub-optimal implementation of policies, and gaps in management competencies (21).

In terms of nurse managers, there have been several empirical studies that have examined the relationship between their management styles and the impact on staff job satisfaction and turnover, patient satisfaction, and quality of care (24–31). These studies have found that transformational and supportive management styles of nurse managers result in lower nurse turnover and higher levels of job satisfaction, which in turn impact positively on patient outcomes (24–31). However, all these studies have concentrated on hospitals, rather than on PHC facilities. In South Africa, there have been a number of studies that focus on nurses working at PHC facilities (32–37). However, the majority of these tend to focus on registered nurses (with 4 years of training) who are the direct service providers (33–35, 37), rather than on PHC clinic managers.

There are several reasons for focusing on PHC clinic nursing managers. Firstly, they are responsible for overseeing the strategic direction of health service delivery, and hence, they play a critical role in the implementation of any health sector reforms (6, 8). Secondly, the literature suggests that effective operational management is positively associated with staff retention, levels of job satisfaction, and quality of patient care (24–31). Thirdly, there is a dearth of information on the perspectives of PHC nursing managers, how they experience or reflect on their work and their practice environment. The aim of this study, therefore, was to explore PHC nursing managers’ work experiences, particularly the successes, challenges or ambiguities faced by them, thereby contributing to recommendations for enhancing management and performance of the health system at the PHC level.

## Methods

### Study setting

The diary study was carried out in Gauteng (GP), an urban province, and Free State (FS), a mixed urban–rural province, as part of a larger doctoral study that included a job satisfaction survey (36). These two provinces were chosen due to geographical proximity to the researchers, budgetary constraints, and prior approval from the health service authorities.

### Ethical considerations

The study was approved by the University of the Witwatersrand's Human Research Ethics Committee (Medical), as well as the relevant provincial and municipal health authorities. The researchers adhered to standard ethical procedures, including detailed participant information sheets, informed consent, and ensuring confidentiality of information.

### Population of interest

The population of interest was professional nurses (with 4 years of training) in charge of 8-hour (day) PHC clinics. These clinics serve catchment populations that range from 10,000 to 180,000 (J. Hunter, personal communication, 2014). The clinics provide preventive services (e.g. immunisation, family planning, and antenatal care), basic curative care for acute and chronic conditions, health promotion, and community outreach services.

### Sampling, recruitment, and data collection

During 2012, a sub-set of 22 nursing managers, 10 in FS and 12 in GP, was selected randomly from an overall survey sample of 111 PHC nursing managers in charge of these 8-hour clinics (36). The details of the job satisfaction survey have been described elsewhere (36).

The event-contingent diary method was used, as participants were asked to record an event that answers a specific research question (38). In our study, we were interested in the qualitative experiences of PHC nursing managers – their successes, challenges, and ambiguities – in the workplace. The reason for the selection of diaries over traditional methods such as in-depth interviews was that it enabled the research team to obtain temporal and/or spontaneous information on work events and nursing manager experiences in the PHC clinic context (39). The diaries also allowed for confidentiality and unguarded responses that are not possible with face-to-face interviews.

Each selected clinic nursing manager was given an information sheet and the voluntary nature of study participation was explained to them. Following informed consent, the diary entry guidelines were explained verbally to each nursing manager. The selected clinic manager was then given an attractive diary, with the guidelines pasted in the front of the diary. Participants were assured of confidentiality and asked not to write their names in the diary.

Each manager was requested to write once a week for a period of 6 weeks about an event that happened at the clinic and that stood out for him/her. Once a week was considered reasonable and realistic for nursing managers who work with limited human resources, and it was done to avoid getting limited or no data at all. Participants had to reflect on why they chose that event, how it made them feel, what they learned from it and what the implications were for their current or future management practice. Clinic managers were also asked to write down the date of the diary entry so that these could be counted during analysis. Participants were encouraged to see the diary as their own personal diary, and the researchers undertook to return the diary to them after completion of data capturing. Participant reminders consisted of mobile text messages and weekly telephone calls.

### Data analysis

The diaries were collected from participants and were stored safely at the researchers’ offices in Johannesburg. The diaries were assigned number codes to prepare for analysis and to ensure confidentiality when returning them to their owners. The diaries were also grouped by province to allow for qualitative comparisons.

The diary entries, hand-written in English by each nursing manager, were typed and saved as individual Microsoft Word documents. During data capturing, we noted that diary entries were longer and more detailed in the first week and shorter in subsequent weeks.

The diary entries were analysed using thematic content analysis (40). The first step in the analysis was to look at participants’ own words and phrases and without preconceived notions or classification. We then examined the language used by each participant in light of the following questions: What do the responses tell us about the experiences, feelings and perspectives of PHC nursing managers? What is emerging about the nature and dynamics of PHC nursing management? What is the ‘lived’ reality of PHC clinic managers’ work experiences?

To ensure reliability, two researchers (an experienced qualitative researcher with health system experience and a nurse academic) participated in the development of the themes by reading the diary entries independently from the first researcher in order to establish inter-coder agreement (40, 41). Following the initial analysis, the team met to discuss the themes generated independently, and to reach agreement on the themes and sub-themes (Table 1). Once agreement was reached on the themes, the diary entries were grouped into the various themes (40). Following the generation of themes, a diary feedback meeting was held with PHC clinic nursing managers. They were asked to comment on the themes, whether the themes represented their work experiences, and to reflect on whether the results obtained represented their working experiences. The feedback meeting provided space for reflectivity and ensured the credibility of the research findings. After the meeting, the diaries were returned to participants.

**Table 1:** Diary entry themes

Theme	Description
Health system deficiencies	Complaints about emergency medical services (EMS) Poor referral system Shortages of medicines or consumables
Human resource challenges	Shortages of all categories of staff (e.g. nurses, pharmacists, cleaners) Staff absenteeism Avoidable mistakes by staff, insubordination, or lack of professionalism
Unsupportive management environment	Negative remarks made by clinic supervisor Tension between supervisor and other district-level managers Poor communication (from supervisor or about meetings) Delays in responding to requests for additional staff Failure to honour appointments Demands for health information (monthly statistics, information for research and/or monitoring, and evaluation purposes)
Leadership and governance	Lack of strategic planning Tensions between clinic manager and staff or senior managers Lack of delegation and authority (e.g. of the budget) Difficulties in managing staff or their performance
Emotional impact of clinic management	Feeling scared, tense, being overwhelmed, feeling abused, burnout, exhaustion, frustration, anger, demotivation Includes personal crises at work or at home Patient, community or political complaints about service delivery Perceived burden of urgent or unscheduled meetings Getting positive feedback from clinic supervisors, or feeling supported Sense of achievement or feeling happy

## Results

Diary entries were all hand-written in English. Fifteen clinic nursing managers participated in the diary study, representing a 68% response rate (GP, *n*=10 and FS, *n*=5). The reasons cited for non-participation by the remaining seven clinic managers were: handing over the individual diary to the courier company who lost it (*n*=5), stolen diary (*n*=1) and lost diary during a motor vehicle accident (*n*=1).

The majority of diary participants were female, with only one male respondent. Close to half of the participants were between the ages of 41 and 50 years (45% *n*=10) and a similar number were above the age of 51 (45% *n*=10), and the remainder were in the 21–30 age group. Participants’ work experience ranged from 5 to 15 years. The majority had been qualified as professional nurses for more than a decade and possessed a PHC clinical training qualification.

In general, the participants took a reflective approach in their diary entries. Although inter-related and not mutually exclusive, the themes that emerged from the diary entries were: health system deficiencies, human resource challenges, unsupportive management environment, leadership and governance, and emotional impact of clinic management. All the themes are shown in Table 1 and summarised separately for the sake of clarity.

### Health system deficiencies

Diary entries revealed several health system deficiencies. These ranged from poor emergency medical services (EMS), shortage of medicines, to lack of an enabling environment for service delivery, such as lack of running water. These deficiencies contributed to the difficulties in managing the clinics, as can be seen from the diary excerpts below.

This is not the first [EMS] incident, but it's definitely the worst in terms of time turnaround …. A patient lost her life having waited for more than two hours for an ambulance. Unless the problem is resolved … more patients will complicate or die waiting for an “emergency vehicle.” [Respondent 3, Gauteng Province]

In some clinics in the FS, diary entries show that clinics would sometimes run out of water and this affected the functioning of the clinic. Managers pointed out that hand washing and other infection control measures were dependent on the availability of water. One FS clinic manager had sought support from the clinic supervisor and the municipality but the problem was not being addressed. Eventually, with support from the community, the clinic had obtained a large plastic water container, to serve as a contingency measure for lack of running water. The example below shows the frustration of this FS nursing manager as she wrote about the lack of water as a recurrent problem:

There is no water in the clinic for three consecutive days …. How will you implement infection control and prevention principles when you work without running water for three days? The clinic gets water cuts frequently – almost every 2–3 weeks …. [Respondent 1, Free State Province]

### Human resource challenges

Human resource challenges were the second major theme that emerged, and the diary entries reflected the negative impact on their management activities. Nursing managers documented wide-ranging responsibilities, including patient consultations, with an apparent disjuncture between their job descriptions and the actual roles they performed in the clinic. Staff shortages impacted on management functions, as managers had to perform clinical duties, in addition to the management functions. In those situations where a staff member was absent or there was a vacant post, nursing managers reported that they had to take on that role, for example, as a pharmacist to dispense medication. High rates of planned and unplanned absenteeism among nursing staff affected clinic operations, and exacerbated the difficulties of PHC clinic management.

I came to work at 7:30 am today realising again nobody came to work. Some are off sick, some just phone to say they will not be coming. Staff shortages are a big problem. I tried to get help again. Nobody from the other clinic can assist. So I must see patients again. All my work is piling up and I did not attend to it yet because of the shortage of staff. Patients are more important than the paper work so I saw patients. [Respondent 7, Gauteng Province]

Staff shortages also led to increased patient waiting times, and in some instances impacted on managers’ health and well-being.

My blood pressure was 156/102 and my glucose level 2.0 mmol/L, I was feeling dizzy and tired. I was unable to go to the doctor because that will mean only one professional will be left at the clinic with more than 100 patients. I never reported the shortage to anyone. The answers that we mostly receive when reporting shortages are “where do you think we will get nurses, find one if you can” that is why we do not report [staff] shortage problems. [Respondent 3, Free State Province]

It was a hectic week, only three nurses on duty on Wednesday and Thursday. I was doing curative [care], adults and children at the same time and I was also busy with statistics in the office. [Respondent 5, Gauteng Province]

The reported staff shortages were exacerbated by perceptions of disabling provincial policies (such as the moratorium on filling posts), staff absenteeism, and an unsupportive management environment.

### Unsupportive management environment

The third major theme that emerged from the diary entries was perceptions of an unsupportive management environment. Clinic managers expressed their disillusionment with their supervisors, who were perceived to be uncooperative and who lacked an understanding of the difficulties faced by them.

My supervisor brought an action plan with time frames. Some of the interventions are not realistic. The clinic was full and staff members were not enough. I was juggling from dispensary, [patient] consulting and solving patients’ minor queries and attending to my supervisor. I feel that I had to give priority to my patients. It was not her [supervisor] visit day according to the schedule. I was disorganised and had to accommodate her …. [Respondent 1, Free State Province]

At times, the diary entries reflected the perceived disrespect, punitive behaviour and verbal abuse from supervisors:

She [supervisor] said there would be no replacement as I only have one entry point in the clinic … she shouted at me that whether I agree or not, she is going to instruct my clerk to go to another clinic which she did … she was so rude and dropped the phone in my ear …. [Respondent 1, Gauteng Province]

On one occasion an FS supervisor did not keep the scheduled appointment, despite calling the clinic manager at her home and giving her instructions for the visit. Nursing managers also complained that clinic supervisors had a top-down approach to supervision and were prescriptive of what needed to be done in the clinics. Supervisors appeared to be unresponsive to requests from clinic managers, especially about additional staff. The excerpt below gives a glimpse of an unpleasant experience of a GP clinic manager.

I had informed the clinic supervisor a month prior to arrange someone for relief [staff] and she had promised to do so. Two weeks before and a week prior, I again reminded her and she still did not know who she was going to send to my clinic to relieve the PHC sister on leave … they sent me someone else whom I was only made aware of that morning. Another professional nurse from the clinic where the relief sister works called demanding that she returns back to her clinic (meanwhile there are four professional nurses in the same clinic) … harassing her that she should return to the clinic. When I checked on her she was tearful and threatening to resign. She found herself torn between wanting to assist at my clinic and being recalled back to her original clinic. This frustrated me even more … the pain … I realised I was … emotionally drained. I called my supervisor who at that time was actually changing from what she said … she now wanted the relief sister to go back to her original clinic while she searched for another one … I refused that the professional nurse leaves the clinic before the relief arrived. After two hours no one arrived. I called again … the nurse was restless and having her bag in hand and was on her way out. This really frustrated me …. [Respondent 2, Gauteng Province]

The above quote reflects the unsupportive approach of clinic supervisors regarding staff shortages and the impact it has on the emotions of nursing managers.

### Emotional impact of clinic management

The multitude of health system problems, human resource challenges, an unsupportive management environment and a range of other problems, coalesced in an overwhelming expression of negative emotions in the diaries, and revealed the emotional impact of PHC clinic management. In some instances, nursing managers wrote about ‘incompetent’ staff reporting to them, and the negative impact on their morale and family life. Importantly, the diary entries reflected the personal stress experienced by these managers at clinics.

I was exhausted … I asked God why I had to come to work with such demotivated staff. I'm starting to hate my work. I know why they are demotivated … they couldn't get study leave, there is no performance management system, even though the population is increasing steadily. It's hard to work with demotivated staff because you must always follow after them for things to be done properly. The thing that hurts the most is that there is no support from co-ordinators of programmes. It's just complaints from patients then staff and from management. Nobody understands the depression we are going through. [Respondent 5, Free State Province]

[My] sleeping patterns are changed because one has to wake up in the early hours of the morning to catch up with administration and [to] meet deadlines. It does not mean one has poor time management but there is a lot of pressure that the manager is subjected to. Family life is also affected by this. Because you come home exhausted, household chores during the week become a challenge. I have sacrificed my weekends and public holidays in order to do my administration. [Respondent 6, Gauteng Province]

PHC clinic managers were frustrated by poor communication from regional and provincial health managers, who often requested information at short notice or summoned them to unplanned meetings. Nursing managers reflected on the perceived burden of these unplanned meetings, despite careful planning on their part. They lamented the lack of control over their average working day as this could be interrupted by ‘an urgent meeting’:

I was very upset on Wednesday. They called me and said there was an urgent meeting and all facility managers must attend. All my plans for the day messed up. [Respondent 12, Gauteng Province]

We were called for an urgent meeting whereby one of our colleagues was together with the supervisor questioning our Regional Health Manager's authority to delegate authority to us as operations managers. Assessing the whole deliberation, I realised that we were caught up in an ongoing misunderstanding and poor communication between the two senior managers … it causes paralysis [Respondent 9, Gauteng Province]

Despite the experience of negative emotions caused by an unsupportive management environment, health system deficiencies and unplanned meetings, clinic managers recognised their important role in health service delivery. They reflected on their responsibilities of: implementing health programmes in the clinic, managing human resources, liaising with community members and relevant stakeholders, and ensuring that clinic operations run smoothly. Notwithstanding the challenges experienced by nursing managers, the diary entries suggest nursing managers who have great concern for patients and the quality of care delivered. One made the following diary entry:

On that morning, the clinic was so full and there were many babies for immunisation and sick adults in the main hall. The passage leading to my office was packed! I had to ask 13 antenatal clients (three new cases) to wait inside the small fourth consultation room. I had to attend to family planning clients and to ARV initiation clients who need to be assessed and have their bloods taken for baseline, to TB patients who were collecting their medication and also referring one very sick (TB/HIV) patient which took almost an hour. [Respondent 1, Gauteng Province]

My “little voice” told me to check the BP (blood pressure) again – it was 240/160!! Severely, severely raised! Apart from now having to treat and refer a pre-eclamptic patient I also realised the terrible risk we take by relying on vital signs taken by a nursing assistant. [Respondent 2, Free State Province]

Despite their crucial role at the PHC level, nursing managers indicated that they seldom receive positive feedback or feel appreciated in the health system. Three entries showed the appreciation of clinic managers when they received positive feedback from their managers or when they felt a sense of achievement:

It was a clinic managers meeting where we were given feedback on programme performance for each clinic. I was told that our tuberculosis (TB) programme had improved since I allocated two professional nurses with the intention of making the programme a success. [Respondent 13, Free State Province]

I came on duty in my culture day dress and it was very nice. No problems this far. Two nurses did not pitch for work but clinic was not that full so I can do my work. I worked out the off duties and started on my report. It was a lovely day and I got all my things done. [Respondent 12, Gauteng Province]

I had local area meeting on Wednesday. I am feeling good because the manager mentioned that our clinic does the best we can with limited resources (staff). I am just glad she realises it. [Respondent 1, Free State Province]

### Leadership and governance

Some nursing managers reflected on feelings of disempowerment, and at times ‘paralysis’, caused by the lack of strategic planning at higher levels of the health system and the difficulties of managing staff reporting to them, the absence of teamwork and their perceptions of a general lack of caring and professionalism on the part of front-line nurses. They complained of the ‘poor work ethic’ among many nurses reporting to them, changing value systems, resistance to change, and lack of accountability. They also reflected on the importance of leadership in nursing given the constant changes in the healthcare system.

However, the diaries revealed that nursing managers do not hold the chain of command in clinics as this power resides with the clinic supervisor and in most instances with the district manager. The local area manager, who oversees several clinics, is responsible for the clinic budget. PHC clinic managers bemoaned the centralisation of the clinic budget, and their lack of control thereof, despite their responsibilities for the day-to-day management of the clinic. The clinic managers were often not consulted on spending priorities, despite compiling annual budget requests. One said the following:

The budget is centralised, one has no power over it. The financial year comes and goes with little improvement … shortages of medicines occur because suppliers are not being paid. [Respondent 1, Gauteng Province]

Clinic managers also receive instructions from doctors, pharmacists, social workers, and vertical health programme co-ordinators responsible for HIV or tuberculosis. All these combine to add further pressure on the clinic manager.

## Discussion

This is one of the first studies to explore the work experiences of PHC nursing managers in two South African provinces using diaries as a research method. The major recurring themes in the diary entries were health system deficiencies, human resource challenges, and an unsupportive management environment – these problems are inter-related and contributed to the difficulties of working in or managing these PHC clinics.

PHC clinic managers expressed frustration with EMS problems and the unpredictable turnaround times, which in one case resulted in a seemingly avoidable patient death. Reliable EMS services have been found to be a critical component of health systems strengthening (42). The nursing managers both reacted and responded to the health system deficiencies in their own way, either by trying to cope with staff shortages or by responding creatively to the lack of water in rural clinics, through partnering with the local community. Other studies have also found that PHC clinic managers often balance operational management and service delivery to many patients amidst staff shortages in the health system (33–35). Although the diary entry on the lack of running water in some FS clinics appears to be an isolated incident, the lack of running water at rural clinics is a common finding in national infrastructure assessments (43). This influences the ability of nurses to comply with infection control standards in these rural clinics, and contributes to the sub-optimal performance of the health system.

The issues highlighted in the diaries resonate with health system deficiencies found in other studies (33, 35, 44, 45). Staff shortages were highlighted in all diaries. The factors that appear to influence these shortages included provincial policies (such as a moratorium on filling of vacant posts), inadequate or poor planning on the part of clinic supervisors, and absenteeism of front-line staff. This meant that PHC nursing managers had to take responsibility for clinical duties often at the expense of their administrative or managerial duties. Although this diary study was small and qualitative, other studies have found that staff shortages have constrained South Africa's ability to achieve the strategic planning goals on HIV and AIDS (46) and the implementation of services at PHC level (34, 47).

The reported staff shortages were made worse by nursing managers’ perceptions of largely unsupportive supervisors. PHC nursing managers wrote about the lack of understanding, disrespect and at times verbal abuse from their supervisors. Notwithstanding the existence of the detailed clinic supervision manual (48), there appears to be a disjuncture between the supervision guidelines in the manual and the clinic managers’ diary reflections of an unsupportive management environment. For example, the manual states that: ‘for the best provision of PHC in facilities, there should be a supervisor who facilitates good teamwork and promotes good working relationships among all the structures of the primary health care system’ (48, p. 4). The lack of quality clinic supervision has been found in other studies as well (49, 50). Effective supervision of PHC clinics is a critical issue that needs to be addressed, given that health sector reforms include a wide range of community-based services and the inclusion of community health workers (47).

In light of the reported challenges experienced by PHC nursing managers, it is not surprising that the diary entries were dominated by an expression of negative emotions, which could be a symptom of the stress experienced by these managers. In response to the question on how the recorded event made them feel, the most frequent responses were: exhausted and frustrated, angry, sad, burnt out, and demotivated. This was borne out by the larger job satisfaction survey, which found that being tired at work and the experience of verbal abuse were predictors of low job satisfaction of these nurses (36). A study in Lithuania among PHC nurses also found that around 60% of nurses experienced negative emotions and resultant emotional stress (51). The Lithuanian study also found that bullying and abuse by supervisors in the workplace caused stress and contributed to feelings of humiliation and disrespect (51). Similarly, a study in Taiwan found that 25% of nursing managers were depressed, 30% suffered from anxiety and 44% suffered from poor quality of sleep leading to high levels of burnout and lower rates of retention (52). Despite some of the negative emotions and experiences recorded in the diaries, overall, the entries reflect a commitment to providing quality care and a need to be acknowledged for their hard work.

There are limitations of this diary study, which was undertaken among a sub-sample of 22 PHC nursing managers. The majority of study participants were from GP, which is the economic powerhouse of South Africa. These clinics are likely to be much better resourced, compared to deep-rural clinics in other parts of South Africa. We had fewer diaries from the FS Province, due to the logistical problems experienced with the courier company. Hence, the study findings may not be transferrable to other PHC clinics in South Africa or elsewhere.

Nonetheless, the findings from the diary study are borne out by the findings of national health system assessments which have highlighted health system deficiencies, human resource challenges and supervision and management problems at the PHC level (44, 45). The diaries are an innovative method of capturing the nature and dynamics of nursing management, as the method allows for confidentiality and anonymity, often not possible with individual interviews or focus group discussions. The diaries gave a voice to PHC nursing managers, facilitated greater self-awareness and allowed them to reflect on their management practices. Nursing managers reported that the diaries were cathartic, as it allowed them to say things that no-one in authority could see or hear. In some instances, the diaries facilitated practical action with identified problems at PHC level, such as when one nursing manager communicated directly with the senior EMS manager after a patient had died. However, the use of diaries requires participant commitment and buy-in, as well as good preparation and initial piloting prior to implementation. It is important to ensure that study participants understand the study objectives and the guidelines for keeping a diary. The success of diary studies depends on regular communication with participants through constant reminders to ensure compliance and maintain the interest of the respondents.

The diary entries have given a glimpse into the difficulties of policy implementation at the local level, from the perspective of PHC nursing managers. These managers give effect to high-level government policies as they are at the interface of community members (and patients) and the formal health system. The PHC nursing managers are expected to manage the bulk of PHC reforms. Their experiences of disempowerment and paralysis need to be addressed through a participatory and inclusive approach, which could simply mean eliciting their views and opinions regarding prerequisites and implementation strategies. This is important because they have to mediate or manage complex health system problems, while ushering in the proposed reforms.

The human side of the managers found expression in a deluge of negative emotions recorded in the diaries. This study has shown that relationships matter and that how they are managed has an impact on how services are delivered or managed. The diary study has also illustrated the resilience among PHC nursing managers and their strategies for coping with a sub-optimal health care system in order to provide adequate care to patients or users. Inflexible hierarchies or policies (e.g. around staff recruitment) appear to make clinic work more onerous, with potential negative consequences for patients and clinic managers. Nursing managers are also curtailed by the centralisation of budget control, and they have to rely on supervisors who do not seem to know how to communicate effectively with them. This lack of delegation of authority, particularly of the clinic budget, exacerbated the reported health system deficiencies. The sense of disempowerment and paralysis experienced by PHC clinic managers was illustrated by the many negative emotions recorded in all the diaries. The relationship between the inability to manage or control the budget and feelings of disempowerment was also found in a 2008 assessment of district managers (21).

Although this was a small, qualitative study, the realities experienced by nursing managers point to issues that need to be addressed as part of the universal health coverage reforms in South Africa. Firstly, efforts to improve the performance of the health system must be comprehensive and recognise that PHC revitalisation must be accompanied by effective and efficient EMS, and appropriate delegation of authority. Secondly, chronic staff shortages require creative strategies, and there appears to be room for improved performance management to reduce staff absenteeism. Thirdly, there are clear guidelines for supportive clinic supervision, which appear to be largely ignored at present. Supervisors may need to be reoriented to the guidelines or receive additional training to enhance their supervision skills. Clinic managers have long experience in the health services, and the health system needs to find a way of harnessing their wisdom in support of current health reforms. Lastly, the identified challenges need to be addressed by policy-makers working together with managers at all levels of the health system, given that health system reforms will create different work demands and diverse experiences for nursing managers.

## Conclusion

This study has highlighted the work experiences of PHC nursing managers, using diaries, a hitherto under-utilised research instrument. The PHC clinic managers’ negative emotions expressed in the diaries have the potential to affect or derail health system reforms, as demoralised PHC nursing managers are unlikely to be champions for change or be committed to such change. At the same time, the PHC nursing managers who participated in the study highlighted the importance of sufficient numbers of health workers, supportive supervisors, and optimal functioning of the health system. The current reform process of South Africa's healthcare system provides a golden opportunity for policy-makers to address the root causes of health system inefficiencies in a participatory manner and through the creation of enabling work environments. To this end, the critical role of the health workforce requires much more attention than is currently the case. Addressing the challenges identified in the work experiences of PHC nursing managers would go a long way in ensuring the successful implementation of health sector reforms.

## References

[1] WHO (2008). The World Health Report 2008: primary health care, now more than ever.

[2] ILO (2014). Universal health protection: progress to date and the way forward.

[3] Rafferty AM, Maben J, West E, Robinson DW (2005). What makes a good employer? Issue 3 of the Global Nursing Review Initiative.

[4] Egger D, Ollier E (2007). Managing the health millennium development goals – the challenge of management strengthening: lessons from three countries.

[5] Global Health Workforce Alliance, WHO (2013). A universal truth: no health without a workforce.

[6] WHO (2009). Who are health managers? Case studies from three African countries. Human resources for health observer.

[7] Campbell J, Buchan J, Cometto G, David B, Dussault G, Fogstad H (2013). Human resources for health and universal health coverage: fostering equity and effective coverage. Bull World Health Organ.

[8] WHO (2007). Towards better leadership and management in health: report on an international consultation on strengthening leadership and management in lower-income countries.

[9] DOH (2010). Negotiated Service Delivery Agreement.

[10] DOH (2011). National Health Insurance in South Africa: policy paper. Government notice: 657 of 12th August 2011, Gazette Number 34523.

[11] DOH (2013). Strategic Plan for Nursing Education, Training and Practice 2012/13–2016/17.

[12] DOH (2010). National Department of Health Strategic Plan 2010/11–2012/13.

[13] Naledi T, Barron P, Schneider H, Padarath A, English R (2011). Primary health care in SA since 1994 and implications of the new vision for PHC re-engineering. South African health review 2011.

[14] Weitz E, Shenhaav Y (2000). A longitudinal analysis of technical and organizational uncertainty in management theory. Organ Stud.

[15] Cooke B, Mills AJ, Kelley ES (2005). Situating Maslow in cold war America: a recontextualization of management theory. Group Organ Manag.

[16] Murman JP, Aldrich HE, Levinthal D, Winter SG (2003). Evolutionary thought in management and organization theory at the beginning of the new millennium: a symposium on the state of the art and the opportunities for future research. J Manag Inq.

[17] Koontz H (1980). The management theory jungle revised. Acad Manag Rev.

[18] Ahonen A, Kallio TJ (2009). On the cultural locus of management theory industry: perspectives from auto-communication. Manag Organ Hist.

[19] WHO (2007). Everybody's business. Strengthening health systems to improve health outcomes: WHO's framework for action.

[20] MSH (2005). Managers who lead: a handbook for improving health services.

[21] Byleveld S, Haynes R, Bhana R, Dudley L, Barron P (2008). A review of structures, competencies and training interventions to strengthen district management in the national health system of South Africa. District management study: a national summary report.

[22] Lehmann U, Barron P, Roma-Reardon J (2008). Strengthening human resources for primary health care.

[23] Lehmann U, Makhanya N, Ijumba P, Barron P (2005). Building the skills base to implement the district health system. South African health review 2005.

[24] Acree CM (2006). The relationship between nursing leadership practices and hospital nursing retention. Newborn Infant Nurs Rev.

[25] Bratt MM, Broome M, Kelber S, Lostocco L (2000). Influence of stress and nursing leadership on job satisfaction of pediatric intensive care unit nurses. Am J Crit Care.

[26] Currie EJ, Carr Hill RA (2012). What are the reasons for high turnover in nursing? A discussion of presumed causal factors and remedies. Int J Nurs Stud.

[27] Hayes LJ, O'Brien-Pallasa L, Duffield C, Shamian J, Buchan J, Hughes F (2006). Nurse turnover: a literature review. Int J Nurs Stud.

[28] Moss R, Rowles CJ (1997). Staff nurse job satisfaction and management style. Nurs Manag.

[29] Cummings GG, MacGregor T, Davey M, Lee H, Wong CA, Lo E (2010). Leadership styles and outcome patterns for the nursing workforce and work environment: a systematic review. Int J Nurs Stud.

[30] Nassar ME, Abdou HA, Mohmoud NA (2011). Relationship between management styles and nurses’ retention at private hospitals. Alexandria J Med.

[31] Raup GH (2008). The impact of ED nurse manager leadership style on staff nurse turnover and patient satisfaction in academic health center hospitals. J Emerg Nurs.

[32] Moyo S, Madale R, Ogunmefun C, English R (2013). Public health and management competency requirements for primary health care facility managers at sub-district level in the District Health System in South Africa. Research snapshot.

[33] Vhuromu EN, Davhana-Maselesele M (2009). Experiences of primary health care nurses in implementing integrated management of childhood illnesses strategy at selected clinics of Limpopo Province. Curationis.

[34] Bester CL, Engelbrecht MC (2009). Job satisfaction and dissatisfaction of professional nurses in primary health care facilities in the Free State province of South Africa. Afr J Nurs Midwife.

[35] Xaba NA, Peu MD, Phiri SS (2012). Perceptions of registered nurses regarding factors influencing service delivery in expanding programmes in a primary healthcare setting. Health SA Gesondheid.

[36] Munyewende P, Rispel LC, Chirwa T (2014). Positive practice environments influence job satisfaction of primary health care clinic nursing managers in two South African provinces. Hum Resour Health.

[37] Delobelle P, Rawlinson JL, Ntuli S, Malatsi I, Decock R, Depoorter A-M (2010). Job satisfaction and turnover intent of primary healthcare nurses in rural South Africa: a questionnaire survey. J Adv Nurs.

[38] Wheeler L, Reis H (1991). Self-recording of everyday life events: origins, types and uses. J Pers.

[39] Roghmann KJ, Haggerty RJ (1972). The diary as a research instrument in the study of health and illness behavior: experiences with a random sample of young families. Med Care.

[40] Miles M, Huberman AM, Saldaña J (2014). Qualitative data analysis. A methods sourcebook.

[41] Denzin NK, Lincoln YS (2000). Handbook of qualitative research..

[42] MacFarlane C, Loggerenberg CV, Kloeck W (2005). International EMS systems in South Africa: past, present, and future. Resuscitation.

[43] Lutge E, Mbatha T (2007). PHC facility infrastructure: a situation analysis of data available.

[44] Integrated Support Teams (2009). Review of health over-spending and macro-assessment of the public health system in South Africa.

[45] Rispel LC, Moorman J, Munyewende P, Meyiwa T, Nkondo M, Chitiga-Mabugu M, Sithole M, Nyamnjoh F (2014). Primary health care as the foundation of the South African health system: myth or reality?. State of the Nation South Africa 2014: South Africa 1994–2014: a twenty-year review.

[46] George G, Quinlan T, Reardon C (2009). Human resources for health: a needs and gaps analysis of HRH in South Africa.

[47] Rispel LC, Barron P (2012). Valuing human resources: key to the success of a national health insurance system. Dev South Afr.

[48] DOH (2009). Primary health care supervision manual: a guide to primary health care facility supervision.

[49] Loveday M, Scott V, McLoughlin J, Amien F, Zweigenthal V (2011). Assessing care for patients with TB/HIV/STI infections in a rural district in KwaZulu-Natal. S Afr Med J.

[50] Van Rensburg HCJ (2014). South Africa's protracted struggle for equal distribution and equitable access – still not there. Hum Resour Health.

[51] Glumbakaite E, Kalibatas J, Kanapeckiene V, Mikutiene D (2007). Connections with sequels of stress and psychological demands on nurses working at primary health care centres. Gerontologija.

[52] Chang EM, Hancock KM, Johnson A, Daly J, Jackson D (2005). Role stress in nurses: review of related factors and strategies for moving forward. Nurs Health Sci.

# Sub article: The activities of hospital nursing unit managers and quality of patient care in South African hospitals: a paradox?

**DOI:** 10.3402/gha.v8.26243

**Published:** 2015-05-11

**Authors:** Susan J. Armstrong, Laetitia C. Rispel, Loveday Penn-Kekana

**Affiliations:** 1 Centre for Health Policy & Medical Research Council Health Policy Research Group, School of Public Health, Faculty of Health Sciences, University of the Witwatersrand, Johannesburg, South Africa; 2 Department of Nursing Education, School of Therapeutic Sciences, Faculty of Health Sciences, University of the Witwatersrand, Johannesburg, South Africa; 3 Department of Infectious Disease Epidemiology, London School of Hygiene and Tropical Medicine, London, United Kingdom

**Keywords:** nursing unit managers, quality of care, hospitals, time and motion, South Africa

## Abstract

**Background:**

Improving the quality of health care is central to the proposed health care reforms in South Africa. Nursing unit managers play a key role in coordinating patient care activities and in ensuring quality care in hospitals.

**Objective:**

This paper examines whether the activities of nursing unit managers facilitate the provision of quality patient care in South African hospitals.

**Methods:**

During 2011, a cross-sectional, descriptive study was conducted in nine randomly selected hospitals (six public, three private) in two South African provinces. In each hospital, one of each of the medical, surgical, paediatric, and maternity units was selected (*n*=36). Following informed consent, each unit manager was observed for a period of 2 hours on the survey day and the activities recorded on a minute-by-minute basis. The activities were entered into Microsoft Excel, coded into categories, and analysed according to the time spent on activities in each category. The observation data were complemented by semi-structured interviews with the unit managers who were asked to recall their activities on the day preceding the interview. The interviews were analysed using thematic content analysis.

**Results:**

The study found that nursing unit managers spent 25.8% of their time on direct patient care, 16% on hospital administration, 14% on patient administration, 3.6% on education, 13.4% on support and communication, 3.9% on managing stock and equipment, 11.5% on staff management, and 11.8% on miscellaneous activities. There were also numerous interruptions and distractions. The semi-structured interviews revealed concordance between unit managers’ recall of the time spent on patient care, but a marked inflation of their perceived time spent on hospital administration.

**Conclusion:**

The creation of an enabling practice environment, supportive executive management, and continuing professional development are needed to enable nursing managers to lead the provision of consistent and high-quality patient care.

Improving the quality of health care delivery is an important global priority (1). Around the world, the purpose of health care quality improvement initiatives is to ensure patient safety, improve clinical effectiveness, and promote public accountability (2). In South Africa, the government has established an independent quality of care regulator, the Office of Health Standards Compliance (OHSC), as part of an overall suite of health sector reforms that aim to achieve universal health coverage (3). The aims of the OHSC are to protect and promote the health and safety of health service users, through the effective management of patient complaints and the enforcement of compliance to prescribed norms and standards (3).

There is well-documented evidence globally that the number, competencies, and effectiveness of nurses are critical in determining the quality of care in hospitals and the nature of patient outcomes (4–14). Nursing unit managers play a key role in coordinating patient care activities and in ensuring safety and quality care in hospital wards. These unit managers are professional nurses registered with the South African Nursing Council (SANC), with at least 4 years of nursing training, and extensive clinical experience. The nursing unit manager (sometimes referred to as ‘charge nurse’ or ‘operational manager’) is responsible for the management of: nursing care to patients; all nursing staff within the unit; and the resources associated with health care delivery in the unit (15). These unit managers in both the private and public health sectors are held accountable for the quality of patient care in their units or wards, and they enter into performance management agreements that outline their operational management responsibilities.

Notwithstanding the importance and commonality of time as an economic resource available to all managers (16), most of the time and motion studies have been conducted among doctors and nurses providing direct care to patients (17–26). The focus of these studies has been on: the relationship between nursing time and quality of care (20, 23, 27); interruptions in nursing tasks and the association with adverse clinical events (21, 28); or to determine the time taken for various nursing tasks in specific nursing units to enable staff planning (17–19, 23–26, 29). The findings of these studies suggest that there is a positive association between the amount of time nurses spend with patients and patient or nurse satisfaction, as well as patient outcomes and safety (17–21). Despite the voluminous literature on time and motion studies (22), the majority of these studies are in high-income countries. Furthermore, with the exception of a 1934 study that focused on ‘head nurses’ in the Unites States (30), we could not find recent studies that focus specifically on the nursing unit manager or studies that concentrate on a low- and middle-income country (LMIC) setting. In light of this dearth of empirical evidence and the major emphasis of the South African government on quality of care, this paper examines whether the activities of nursing unit managers facilitate the provision of quality patient care in selected South African hospitals. The specific study objectives were to: record the time taken by nursing unit managers on various activities in the hospital ward; determine the perceptions of these unit managers regarding their own time management; and examine whether the range and diversity of nursing unit manager activities facilitate quality of care in their units. The findings reported in this paper are part of a larger research programme to examine the relationship between nursing management and quality of care.

## Methods

### Ethical considerations

The University of the Witwatersrand Human Research Ethics Committee (Medical) granted ethical approval for the study. The public and private health care authorities in the two study provinces also provided study approval. All participants received a study information sheet and were required to sign an informed consent form to indicate their willingness to participate in the study.

### Research setting

Two South African provinces, Gauteng and the Free State, were selected purposively because of geographical proximity to the researchers, health authority approval, and budgetary constraints. Gauteng is the most urbanised and densely populated province with a population of 12.2 million, while the Free State province is a mixed urban–rural, largely agricultural province with a population of 2.7 million (31).

### Sampling

The study sample was not intended to be representative of all unit managers in the country, but rather to explore issues of nursing management, quality of care, and clinical governance at hospital unit level that could serve as a basis for future research. Budgetary constraints also influenced the number of hospitals and units selected for in-depth study.

In the Gauteng province, three hospitals were selected from the public health sector, and three from the private health sector. In the case of the public sector hospitals, one hospital was selected randomly from each cluster of tertiary, regional, and district hospitals (total of three hospitals). All specialised psychiatric and tuberculosis hospitals were excluded. In the case of the private hospitals, only non-specialised hospitals with more than 100 beds were included in the sampling frame. One hospital was selected randomly from each of the three major private hospital groups in South Africa (total of three hospitals).

In the Free State province, only public hospitals were included, with one hospital selected from each of the cluster of tertiary, regional, and district hospitals (total of three hospitals). At each hospital, one medical, surgical, paediatric, and maternity (labour and post-natal) unit was selected (total four or five units per hospital depending on the size of the hospital). In hospitals with more than one of these units, a simple random sampling technique was used to select the unit. A total of 36 units were selected.

The final sample consisted of six public sector hospitals out of a total of 54 public hospitals, and three private hospitals out of a total of 99 private hospitals in the two study provinces.

### Study participants

All the nursing unit managers (*n*=36) in charge of the selected hospital units were invited to participate in the study.

### Data collection and analysis

The data was collected over a 4-month period from September until December 2011. There were two components to the data collection: a time and motion study which involved continuous and independent observation of unit managers’ work activities; and semi-structured in-depth interviews to determine the managers’ perceptions of their own time use and management.

Following informed consent, each selected unit manager was observed by a trained, nurse-field worker for a period of 2 hours on the survey day (1 hour in the morning and one in the afternoon) and the activities recorded on a minute-by-minute basis. The fieldworkers were trained to be as discreet as possible and not to interfere with the work of the unit manager.

Hence, there was a total of 72 hours of observation. The activities were entered into Microsoft Excel and coded into categories. The research team used thematic content analysis to develop the themes or categories (32). One researcher read through all the recorded activities of nursing unit managers to get an overview of the findings and to highlight important issues that emerged (32). A distinction was then made between the main, unique, and other themes that emerged from the data. A second researcher read a sample of the recordings independently and also recorded the issues that emerged from the data. The researchers met to discuss the emerging themes in light of the study objectives and to reach agreement on these themes or categories of activities (Table 1).

**Table 1:** Categories of nursing managers’ activities identified from time and motion study

Category	Brief description
Patient care	Includes the assessment of patients or providing, checking, directing, discussing, organising, or coordinating care
Hospital administration	Management or quality assurance meetings, discussion with individuals, private or corporate organisations
Patient administration	Admissions and discharges, completing or checking nursing records, checking medical or essential equipment
Education	Includes patient or junior staff education, or organising continuing professional development activities
Communication and support	Includes telephone calls and communication or support provided to all categories of staff (doctors, nurses, other staff members), patients, their relatives or visitors, students
Equipment and stock management	Ordering, checking, receiving, distributing, or locating items or consumables
Staff management	Directing, correcting, orientating, sourcing, allocating and delegating, conflict resolution, and staff evaluation
Miscellaneous (other)	Walking around and seeking items, ward hygiene, tidying, maintenance and support services, breaks – lunch/tea/rest

After agreement was reached on the categories, the activities were coded and analysed according to the time spent on activities in each category. Microsoft Excel was used to analyse the data and to determine the time spent on the various categories of activity by each unit manager and the average time spent on each category of activities when the data was combined for all nursing unit managers.

A semi-structured in-depth interview was conducted with each nursing unit manager, who was asked to think about and reflect upon the previous working day. The manager was asked to indicate which three activities or tasks took up most of their time, and whether there were any activities or tasks that they wanted to get done, but just could not find the time to do. They were also asked whether there were daily tasks that take up too much of their time; and/or tasks that they do not have time to get round to. Each interview was recorded and transcribed verbatim. The information related to their perceptions of the activities in which they had engaged the previous day were analysed using thematic content analysis (32). The activities mentioned by the nursing managers were listed, as well as their estimates of the time spent on each activity. The self-reported information obtained from the inductive analysis was compared with the codes derived from the time and motion study described above, and found to be similar. The research team listed additional emerging themes separately.

Following the generation of themes, a workshop was held with the nursing unit managers. They were asked to comment on the analysis of their daily activities, the categories and themes, and to reflect on whether the results obtained represented their management experiences. The feedback meeting provided space for reflectivity and ensured the credibility of the research findings.

## Results

### Time and motion study

All nursing unit managers agreed to participate in the study. Eight categories of activity were identified from the observations of the unit managers’ activities. The time and motion study found that nursing unit managers spent 25.8% of their time on direct patient care, 16% on hospital administration, 14% on patient administration, 3.6% on education, 13.4% on support and communication, 3.9% on managing stock and equipment, 11.5% on staff management, and 11.8% on miscellaneous activities (Fig. 1).

**Fig. 1:** Nursing unit managers’ activities in the time–motion study.

As can be seen from Fig. 1, unit managers spent 25.8% of their time on direct patient care (Fig. 1), which was more time spent than on any other single group of activities. These activities took place at the patient's bedside and included direct patient care such as positioning the patient, assisting new mothers to breastfeed, administering analgesia, or assisting patients to eat. The nursing manager indicated that they felt obliged to provide this basic nursing care, rather than delegate it to a more junior nurse. The provision of direct patient care typically occurred while on the way to perform a more advanced nursing task such as administering intravenous medication or monitoring a blood transfusion. Much of the time spent coordinating care resulted from the doctors’ ward rounds, with the manager ensuring that the treatment was given or that X-rays and blood tests were done. In the public hospitals where a doctor did a ward round once a day, the unit managers were able to organise all treatment at one time, which greatly assisted with time management. This task became very time consuming in the private hospital units where private doctors may have one or two patients in the ward and arrive at a time that suited them which resulted in the unit managers having to stop other activities to deal with the instructions of a single doctor.

Nursing unit managers spent an average of 16% on hospital administration. Meetings took up a significant part of the unit manager's time, particularly in the public hospitals where all the unit managers would be called to meetings with the nursing service manager, lasting more than 1 hour at a time. At the time of the fieldwork, the one province was expecting accreditation visits to be carried out within the month. The time spent on quality assurance activities at these provincial hospitals was significant with one unit manager spending her entire day making sure that all hospital records were ready for the inspection.

Only 3.6% of unit manager's time was spent teaching staff and students despite all of the selected hospitals being training facilities. Nursing students were present in many of the wards but the unit managers did not appear to see teaching as part of their responsibilities. However, the managers corrected staff and students when they observed the provision of inadequate care.

Unit managers spent an average of 3.9% on managing stock and equipment, but this varied depending on the presence of a ward clerk in the unit. One hospital had devolved the management of stock and equipment to the responsible department and did not hold the unit manager accountable for managing stock. For example, in the case of medicines, the pharmacist was responsible for checking stock levels, ordering, and supplying the appropriate medicines.

The study found that 11.8% of the nursing unit manager's time was spent on miscellaneous activities, with almost half of this proportion (48%) spent on walking and in search of stock, keys or other staff members. In several of the units, the unit manager was the only person entrusted with the stock and medicine keys resulting in her having to walk back to the cupboards every time a junior staff member needed medication or other items for patient treatment. Unit managers preferred to record information in patient files in their offices or at the nurses’ stations, hence time was spent time fetching these patient files. In the public hospitals, unit managers collected medicines or consumables personally from the pharmacy or store-rooms respectively, because they reportedly had greater powers of persuasion, compared to junior staff members who were less likely to obtain these necessary items.

### Perception of nursing managers of time spent on different activities

Figure 2 shows the perceptions of nursing managers of the time spent on different activities.

**Fig. 2:** Nursing unit managers’ perceptions of time spent on different activities.

As can be seen from Fig. 2, 24% of the activities identified by nursing unit managers related to patient care, 30% to hospital administration, 16% support and communication, 12% on patient administration, 8% stock and equipment, with the remaining time spent on staff management, and education.

### Interruption experienced by nursing managers

The time and motion study found that a nursing unit manager had to deal with 36 different types of activities in 1 hour. In one instance, it took a nursing manager 30 minutes to make an entry in a patient record, because of all the interruptions that ranged from answering the telephone, responding to patient visitors, providing support to doctors or assisting junior nurses. In order to prevent these interruptions, unit managers frequently completed their management reports in an area away from the unit.

### Factors influencing unit managers time utilisation and management

The interviews revealed that several issues influenced the time spent by nursing managers on the various activities, as well as the management of their time. These were: health workforce issues (shortages, staff performance); sub-optimal communication; resource constraints; and unplanned activities and interruptions. Each of these is explored briefly.

A recurring theme that emerged from the interviews was issues related to the health workforce, particularly staff shortages and sub-optimal staff performance, the latter influenced by their perceived competencies. Unit managers particularly in the public hospitals made several references to staff shortages which reportedly interfered with their management responsibilities, illustrated by the following comments.

I was so short staffed I didn't do any managerial duties. (Respondent 15, public paediatric unit)

I didn't get round to off duties. I was particularly understaffed yesterday as the staff nurse was on a [training] course and students were in class. (Respondent 2, public surgical unit)

The performance of staff was another concern for unit managers. Absenteeism of nurses with little or no notice and seemingly suspect reasons caused a lot of frustration among the nursing unit managers. A private hospital maternity unit manager gave an example of the incompetence of a nurse in her unit, whom she felt she could not trust, and this impacted on the time she (the nursing manager) spent on various activities.

I didn't have time to sit and talk with a nurse who other staff members have been complaining that she isn't doing her work. I need to deal seriously with this issue. Keep a record of complaints etc. Need to build a case – and work out what to do. Letting it linger could lead to a problem with a patient. (Respondent 28, private maternity unit)

Nursing managers in both public and private hospitals reported problems of sub-optimal communication, particularly poor record-keeping that influences patient care, as well as inadequate communication between doctors, patients, and nurses. A manager from a private paediatric unit said:

We (have to) compensate for the fact that the doctors can't explain clearly to the patients. (Respondent 35, private paediatric unit)

This same problem was reported in another private hospital paediatric unit where the manager said:

Supervision- you waste so much time … checking records that keep being wrong. (Respondent 15, private paediatric unit)

Although the nature and magnitude of resource constraints were different between public and private hospitals, all the unit managers complained that this aspect made their jobs more challenging. In both the public and private hospitals, unit managers complained about the time taken, and difficulties, of finding beds. The bed shortages resulted at times in premature discharge of some patients, and admission of new, very ill patients.

I spent 2 hours with the CEO [chief executive officer] trying to sort out beds and equipment issues. (Respondent 4, public post-natal unit)

In the private sector, unit managers complained about the time spent in finding agency nurses.

Finding beds and finding agency staff takes a lot of time. (Respondent 31, private paediatric unit)

I didn't have time (for important issues because I was) checking agency staff – hours worked, salaries paid etc. (Respondent 28, private post-natal unit)

### Perceptions of unplanned activities and interruptions

All unit managers raised unplanned activities and interruptions as a major problem. Some of these were unforeseen, such as the suicide of a staff member or medical emergencies such as cardiac arrests, unplanned caesarean sections or transfer of patients to intensive care units. These were common to both public and private hospital unit managers, as can be seen from the comments below:

I spent lots of time with a patient who needed to be sent to ICU because she didn't have an [urine] output. (Respondent 16, public post-natal unit)

I had a resuscitation that I didn't expect – it took 2 hours of my time. (Respondent 29, private medical unit)

I had two critically ill babies and I struggled to get a doctor. (Respondent 15, public paediatric unit)

Medical emergencies had occurred in four of the units on the day prior to the fieldwork.

Unit managers also complained about the time taken for orientation of new staff members or community service nurses, record audits, and quality assurance activities. They also indicated that telephone enquiries from relatives or staff, in the absence of support staff such as clerks, take up a great deal of time and detract from patient care and management activities.

More than one third of nursing unit managers complained about administration and that meetings take up too much of their time. One said that there is so much administration that she feels like a clerk at time, while others said the following:

There is so much admin, and meetings. For the ward and hospital, I spend a lot of time travelling between hospitals and capturing data for the DHIS [district health information system]. We could do with some secretaries and supervisors for support staff. (Respondent 23, public paediatric unit)

We keep talking about the problem – but it is never solved - the meetings, meetings, meetings, take up a lot of time. (Respondent 12, public maternity unit)

The executive nurse manager of one of the hospitals was also in charge of another hospital, 50 km away. This meant that every time a meeting of the unit managers was called, at least one set of nursing managers had to travel, resulting in an entire day away from their units.

## Discussion

The time and motion study found that on average, nursing unit managers in the selected hospitals spent around one quarter (25.8%) of their time on patient care activities. This was similar to the proportion (26%) that the unit managers estimated to spend on patient care. When probed about the amount of time spent on patient care, they indicated that they had to care for the patients themselves as there was no alternative, either because of the complexity of the nursing task or because of staff shortages. We could not find similar studies focusing on nursing unit managers in other LMIC settings. Although not directly comparable, a 2008 study in a medical ward in Australia found that nurses spent 33.2% on direct patient care (33), while another Australian study in two wards in a teaching hospital found that nurses spent 37% of their time with patients (20). Similarly a Belgian study found that nurses spent 32.2% of their time on direct patient care (24), while a similar proportion of 32.8% was found in a Montreal hospital study among surgical nurses (17). The 1934 US study found that ‘head nurses’ or unit managers spent around 15.6% of their time on direct patient care (30), while another US study found that nurses spent 44% of their time on patient care (19).

There is no norm of the proportion of time that nursing unit managers should spend on patient care. Although it is encouraging that unit managers spent such a large proportion of their time on patient care, it could mean that they may not have enough time to carry out their primary management responsibilities. These duties include the strategic management of patient care activities in the unit (e.g. co-ordination of patient care, overseeing quality of care initiatives), teaching and mentoring of students and junior staff, and the management of human resources, finances, equipment, pharmaceuticals, and other resources.

Although unit managers spent 25.5% of their time on patient care, our study found that they engaged in 36 different tasks per hour, averaging less than 2 minutes per task. Although this was better than the average of 72.3 tasks per hour in an Australian study, our study finding implies that unit managers perform numerous, fragmented tasks of short length. This could mean that changes in a patient's condition may not be noticed. Nursing scholars have argued that fragmented patient time is not conducive to nursing surveillance (34, 35), which is considered essential for quality of care, especially the identification and prevention of medical errors and adverse events (35).

In our study, unit managers spent a sizeable proportion of their time on hospital (16%) or patient (14%) administration. The perceptions of nursing managers were that they spent around 30% of their time on hospital administration, with meetings being the most frustrating aspect mentioned. The discrepancy between actual (observed) and perceived proportion of time spent on hospital administration could be due to different perceptions of roles and responsibilities among nursing managers, lack of knowledge on these roles and responsibilities, and/or because they found administration to be less satisfying, than providing direct patient care. Other studies have found that the proportion of time spent on administration ranged from a low of 6% in an Australian study (33) to 33.1% in the 1934 study of ‘head nurses’ (30).

One of the most striking aspects of this study was the number of interruptions the unit managers experienced. An interruption is defined as a ‘a break in performance that occurs in response to a source that is external or internal to a person, e.g. daydreaming, suspension of the initial task, performance of another task, and resumption of the primary task’ (36, p. 2). While the interruptions and the unplanned activities were not unexpected, the nature of some of these gave insight into the practice environment of the unit managers. Other studies have demonstrated that task interruptions affect patient safety as they could increase medication errors or adverse events (21, 27, 28, 36). Interruptions also have a negative impact on work procedures, work flow, ability to concentrate, reflective processes, and interaction with patients (28, 37).

The study found that nursing unit managers spent 11.5% on staff management, but the self-assessment of the unit managers was that they spent only 5% on this aspect. The 1934 US study found that the head nurses spent 22.5% on ‘supervision’ (30), but the studies are not directly comparable. At the same time, the interviews revealed that unit managers seem to shy away from active performance management of staff, rather than deal with problems of absenteeism or complaints about staff performance directly.

We also found that 11.8% of time was spent on miscellaneous activities (Fig. 1), but this did not feature as a category mentioned by the unit managers. As indicated, half of the time is spent simply on walking and looking for stock, keys or other staff members. This suggests a rather centralised system of unit management, rather than a more distributed leadership model.

During the interviews, unit managers discussed the factors impacting on their time management and they spoke passionately about their frustrations with time-consuming activities, which they believe have a negative impact on the quality of patient care in their units. The four major themes were health workforce issues, resource constraints, sub-optimal communication, and interruptions and unplanned activities.

There are limitations of this study, which was undertaken among 36 nursing unit managers in nine hospitals. The study sample is small, and no analysis of variance was possible between the public and private hospitals, urban and rural hospitals, or among different hospital units. The findings may have limited generalisability to other hospitals, both in South Africa or other LMIC settings. The observation period was relatively short, and this may have influenced the results obtained. Although the fieldworkers tried to be discreet during the observation of nursing managers, their presence could have influenced the behaviour of the nursing managers. The data collection instruments were designed for our study aims, making cross-study comparability difficult. The in-depth interviews elicited self-reported information from nursing unit managers on their time management and the time spent on different types of activities. The results may reflect social desirability bias, with nursing unit managers over-estimating the time spent on hospital administration. We did not ask nursing managers about their perceptions of their roles and responsibilities, or what they considered to be the ideal time to spend on different types of activities. We are unable therefore to determine whether the results obtained could be explained by possible differences in unit managers’ perceptions of their roles and responsibilities. This is an area for further research.

Nonetheless, there are several study strengths. This was one of the first studies in South Africa and in a LMIC setting to examine the work activities of nursing unit managers, and to combine direct observation with in-depth interviews with these managers. The study findings could be used as a baseline for larger, national studies on clinical governance at hospital unit level, and the methods could be validated and/or enhanced in future research. The study findings were validated during a workshop with the nursing managers, thus giving voice to these individuals. The interaction with nursing managers also allowed the nursing managers to be reflective, and to learn from the experiences of other managers, both in the public and private health sectors.

The study findings provide a glimpse into the difficulties of achieving a quality of care revolution as envisaged by the establishment of the independent OHSC in South Africa. Nursing unit managers are the custodians of professional nursing practice, quality patient care, and safety in hospitals. Hence, the issues identified in this study must be addressed to ensure that they fulfil their clinical leadership role in hospitals.

In the short-term, support from the hospital executive management, particularly the executive nursing manager, is critical. At the very least, a forum that brings together the different unit managers in each hospital would be a step in the right direction. Such a forum would allow for sharing of good practices, peer-to-peer mentoring, and the development of solutions to common problems such as dealing with difficult staff members or mechanisms to improve communication among all members of the health team.

In those study hospitals that employed ward clerks or that used an electronic patient information system, the workload of unit managers was eased considerably. In the public hospitals, the idea of ward clerks to relieve the unit manager of routine administrative work has been revived, but implementation has been patchy. The employment of ward clerks would be an important intervention. Another approach, which appears valuable in one province and in the private health sector, was the requirement that medicines and supplies were managed by the relevant departments and not by the unit manager. Unit managers were involved in determining minimum and maximum stock levels, after which other categories of staff keep control of ordering and delivery of stock.

In the private sector, the employment of a ‘shift leader’ appears to assist with the problem of interruptions of the nursing unit manager. This individual is a registered nurse who takes responsibility for coordinating patient care, thus allowing the unit manager to concentrate on her management or leadership responsibility. Although this solution depends on the budget availability for an additional registered nurse, the additional cost of employing a shift leader may be off-set by an increase in patient satisfaction and a reduction in adverse events. However, this is an area for further research.

We recommend that continuing professional development programmes be developed for nursing unit managers to enhance their management skills and abilities. These should include, *inter alia*, training or coaching in delegation, conflict management (doctors, fellow nurses, patients, relatives of patients), human resource management, time management, organisational skills, leadership for quality assurance, as well as unit management. The roles and responsibilities associated with unit management should also be emphasised. These aspects should also be addressed in the pre-service education and training of registered nurses.

Although our study concentrated on the nursing unit manager, our study findings suggest that problems in the practice environment affect all categories of staff, including junior nurses. In the primary health care environment, a survey found that positive practice environments influence the job satisfaction of managers (38). Globally, there has been strong advocacy for positive practice environments that enable high quality of care to be delivered (5, 9, 39). In South Africa, there is an enabling policy environment for the delivery of quality of care (3, 40), but much needs to be done to ensure actual implementation of these policies that will create positive practice environments.

## Conclusion

The study examined whether the activities of nursing unit managers facilitate the provision of quality patient care in South African hospitals. The answer is not straightforward. On the positive side, the study found that 25.5% of the time of the nursing unit managers is spent on patient care. Although this proportion compares well with the findings of studies among nurses providing direct care in high-income countries (17, 19, 20, 24, 33), this proportion might not be appropriate in light of the core management responsibilities of nursing unit managers. The study also found that unit managers experienced numerous interruptions, performing many short, fragmented tasks. A significant proportion of time was spent on miscellaneous activities, which provides an opportunity for intervention. The practice environment with staff shortages and performance problems, resource constraints, sub-optimal communication, and unplanned activities exacerbate the difficulties of the unit manager to provide leadership for the delivery of high-quality patient care.

This study has highlighted the work activities of nursing unit managers, and has explored its relationship to the provision of quality care in selected hospitals. The creation of an enabling practice environment, supportive executive management, and continuing professional development are needed to enable nursing managers to lead and oversee the provision of consistent and high-quality patient care in these South African hospitals.

## References

[1] Varkey P, Reller MK, Resar RK (2007). Basics of quality improvement in health care. Mayo Clin Proc.

[2] Shaw C (2004). The external assessment of health services. World Hosp Health Serv.

[3] Republic of South Africa (2013). National Health Amendment Act No. 12 of 2013.

[4] Aiken L, Clarke S, Sloane D, Sochalski J, Silber J (2002). Hospital nurse staffing and patient mortality, nurse burnout, and job dissatisfaction. JAMA.

[5] Aiken LH, Sermeus W, Van den Heede K, Sloane DM, Busse R, McKee M (2012). Patient safety, satisfaction, and quality of hospital care: cross sectional surveys of nurses and patients in 12 countries in Europe and the United States. BMJ.

[6] You L-M, Aiken LH, Sloane DM, Liu K, He G-P, Hu Y (2013). Hospital nursing, care quality, and patient satisfaction: cross-sectional surveys of nurses and patients in hospitals in China and Europe. Int J Nurs Stud.

[7] Teng C-I, Shyu Y-IL, Chiou W-K, Fan H-C, Man Lam S (2010). Interactive effects of nurse-experienced time pressure and burnout on patient safety: a cross-sectional survey. Int J Nurs Stud.

[8] Kirwan M, Matthews A, Scott PA (2013). The impact of the work environment of nurses on patient safety outcomes: a multi-level modelling approach. Int J Nurs Stud.

[9] Ausserhofer D, Schubert M, Desmedt M, Blegen MA, De Geest S, Schwendimann R (2013). The impact of the work environment of nurses on patient safety outcomes: a multi-level modelling approach. Int J Nurs Stud.

[10] Cho S, Ketefian S, Barkauskas VH, Smith DG (2003). The effects of nurse staffing on adverse events, morbidity, mortality, and medical costs. Nurs Res.

[11] Mueller M, Lohmann S, Ralf Strobl R, Boldt C, Grill E (2010). Patients’ functioning as predictor of nursing workload in acute hospital units providing rehabilitation care: a multi-centre cohort study. BMC Health Serv Res.

[12] Unruh LY, Hassmiller SB, Reinhard SC (2008). The importance and challenge of paying for quality nursing care. Policy Polit Nurs Pract.

[13] Wunderlich GS, Sloan F, Davis CK (1996). Nursing staff in hospitals and nursing homes: is it adequate?.

[14] Kurtzman ET, Corrigan JM (2007). Measuring the contribution of nursing to quality, patient safety, and health care outcomes. Policy Polit Nurs Pract.

[15] Department of Public Service and Administration (1999). Code of remuneration (core) occupational category: nursing and support personnel. Core code: 00801.

[16] Braithwaite J, Westbrook MT (2011). Time spent by health managers in two cultures on work pursuits: real time, ideal time and activities’ importance. Int J Health Plann Manage.

[17] Desjardins F, Cardinal L, Belzile E, McCusker J (2008). Reorganizing nursing work on surgical units: a time-and-motion study. Nurs Leadersh.

[18] Hendrich A, Chow MO, Skierczynski BA, Lu Z (2008). A 36-hospital time and motion study: how do medical–surgical nurses spent their time?. Perm J.

[19] Storfjell JL, Omoike O, Ohlson S (2008). The balancing act: patient care time versus cost. J Nurs Adm.

[20] Westbrook JI, Duffield C, Li L, Creswick NJ (2011). How much time do nurses have for patients? A longitudinal study quantifying hospital nurses’ patterns of task time distribution and interactions with health professionals. BMC Health Serv Res.

[21] Westbrook JI, Woods A, Rob MI, Dunsmuir WTM, Day RO (2010). Association of interruptions with an increased risk and severity of medication administration errors. Arch Intern Med.

[22] Zheng K, Guo MH, Hanauer DA (2011). Using the time and motion method to study clinical work processes and workflow: methodological inconsistencies and a call for standardized research. J Am Med Inform Assoc.

[23] Munyisia EN, Yu P, Hailey D (2011). How nursing staff spend their time on activities in a nursing home: an observational study. J Adv Nurs.

[24] Myny D, Van Goubergen, Limere V, Gobert M, Verhaeghe S, Defloor T (2010). Determination of standard times of nursing activities based on a nursing minimum data set. J Adv Nurs.

[25] Fiedler KM, Weir PL, van Wyk P, Andrews DM (2012). Analyzing what nurses do during work in a hospital setting: a feasibility study using video. Work.

[26] Furåker C (2009). Nurses’ everyday activities in hospital care. J Nurs Manag.

[27] Kalisch BJ, Aebersold M (2010). Interruptions and multi-tasking in nursing care. Jt Comm J Qual Patient Saf.

[28] Sörensen EE, Brahe L (2014). Interruptions in clinical nursing practice. J Clin Nurs.

[29] Ballermann M, Shaw NT, Mayes DC, Gibney N, Westbrook JI (2011). Validation of the Work Observation Method By Activity Timing (WOMBAT) method of conducting time–motion observations in critical care settings: an observational study. BMC Med Inform Decis Mak.

[30] Gordon PA (1934). Time study of head nurse activities. Am J Nurs.

[31] Statistics South Africa (2012). Census 2011: statistical release (revised) P0301.4.

[32] Creswell JN (2003). Research design-qualitative and quantitative approaches.

[33] Chaboyer WM, Wallis M, Duffield C, Courtney M, Seaton P, Holzhauser K (2008). A comparison of activities by enrolled and registered nurses on medical wards in Australia: an observational study. Int J Nurs Stud.

[34] Kelly L, Vincent D (2011). The dimensions of nursing surveillance: a concept analysis. J Adv Nurs.

[35] Henneman EA, Gawlinski A, Giuliano KK (2012). Surveillance: a strategy for improving patient safety in acute and critical care units. Critical Care Nurse.

[36] Brixey J (2009). Interruptions in workflow for RN's in a level one trauma centre. Quality Health Care.

[37] Cellier J, Eyrolle H (1992). Interference between switched tasks. Ergonomics.

[38] Munyewende P, Rispel LC, Chirwa T (2014). Positive practice environments influence job satisfaction of primary health care clinic nursing managers in two South African provinces. Hum Resour Health.

[39] Global Health Workforce Alliance, WHO (2013). A universal truth: no health without a workforce.

[40] DOH (2013). Strategic Plan for Nursing Education, Training and Practice 2012/13–2016/17.
